# Sample size requirements to evaluate policies in addiction research using interrupted time series analysis (ITS): Tools and guidance

**DOI:** 10.1111/add.70220

**Published:** 2025-11-11

**Authors:** Emma Beard, Jamie Brown, Lion Shahab

**Affiliations:** ^1^ Department of Epidemiology and Public Health University College London London UK; ^2^ Research Department of Behavioural Science and Health University College London London UK; ^3^ SPECTRUM Consortium UK

**Keywords:** ARIMA, ITS, policy evaluation, power, sample size, time series

## Abstract

Formal power calculations are rarely presented in interrupted time‐series (ITS) studies due to their technical complexity, creating a significant gap in methodological rigor. This paper aimed to make power and sample size determination more accessible for researchers, particularly in the field of addiction, by providing a suite of practical and user‐friendly tools. A set of resources was developed using Monte Carlo simulation to allow researchers to estimate statistical power under a wide range of ITS design parameters. The approach allows for the explicit definition of the data‐generating process, including specific autocorrelation error structures (ARMA), the presence of covariates and trends and different intervention effect types (step, pulse and trend change). The study produced three key resources: (1) a flexible R code base for conducting custom power simulations, (2) an intuitive, interactive R Shiny App that enables code‐free power analysis through a web interface and (3) a series of pre‐calculated look‐up tables for quick sample size estimation during the initial stages of study design. Illustrative examples from addiction research demonstrate the tools' application. The provided tools bridge a critical gap by simplifying the process of conducting rigorous power calculations for ITS designs. Their adoption can enhance the planning, execution and interpretation of quasi‐experimental studies, helping to ensure that research is adequately powered to detect meaningful policy and intervention effects.

## INTRODUCTION

The gold standard to evaluate the causal effect of an intervention is through the conduction of randomised controlled trials. However, these have limitations in terms of cost and are also often unpractical when assessing interventions occurring in the real world. One solution is the use of interrupted time series (ITS) analysis, a quasi‐experimental design that aims to assess whether an event or shift in policy is associated with a change in a target variable using pre‐ and post‐intervention data, but without randomisation. For example, this has been used to evaluate the effect on smoking prevalence of the partial tobacco display ban at point of sale in large shops [1] and the suspension of tobacco mass media campaigns in England [2]. It has also been used to assess the impact of minimum unit pricing on alcohol sales in Scotland [3], and the effectiveness of overdose education and nasal naloxone distribution on opioid overdose in the USA [4].

Although ITS analysis can be a powerful tool for evaluating public health policies and behavioural interventions, there are no widely accepted standards for calculating the necessary sample sizes for ITS studies [5]. As a result, few published studies discuss the power of the planned study or analysis, and those that do often lack formal power calculations [6, 7]. This presents a significant issue because a non‐significant result in an underpowered study does not necessarily indicate the absence of an effect.

Many researchers turn to rules of thumb when conducting ITS analyses. For example, it has been suggested that a minimum of 50–100 observation periods are required [8, 9], with more observation periods than model parameters [10], and with equal proportions of data collection before and following an intervention [11]. The problem with rules of thumb such as these is that they do not consider variability in the data, the effect size of interest or the distribution of the data [10, 12, 13]. Attempts have also been made to devise analytical power methods for time series analysis [14, 15], which require one to specify the number of observed time series values overall, before and following the intervention, and the level of autocorrelation. Although this closed‐form power calculation may be suitable for most study designs, and has the advantage of being fast and easy to implement, it breaks down when one considers designs with complex data‐generating processes or complex statistical analyses [16, 17, 18, 19, 20].

One of the most common analyses for ITS is known as autoregressive integrated moving average (ARIMA) modelling, which captures various forms of temporal dependencies. The ARIMA acronym refers to its three core components, known as Autoregression (AR) autocorrelation, Integration and Moving Average (MA) autocorrelation:

- The AR component indicates the order of the AR autocorrelation, which occurs when current values of the time series are linearly dependent on their own past values. For example, an AR(1) model suggests that the current observation is influenced by the immediately preceding observation, an AR(2) model is influenced by the two preceding observations, and so on.
- The Integration component addresses a common issue in time series data: non‐stationarity. A time series is considered non‐stationary if its statistical properties, such as its mean, change over time. To handle this, differencing is applied. This is a transformation that, instead of modelling the absolute values of the data, models the difference between consecutive observations.
- The MA component indicates the order of the MA autoregression, which occurs when current values of the time series are linearly dependent on a combination of past error terms (also known as ‘shocks’ or ‘innovations’). These error terms represent the random fluctuations in the series that are not explained by the AR or integration components.

**FIGURE 1:**
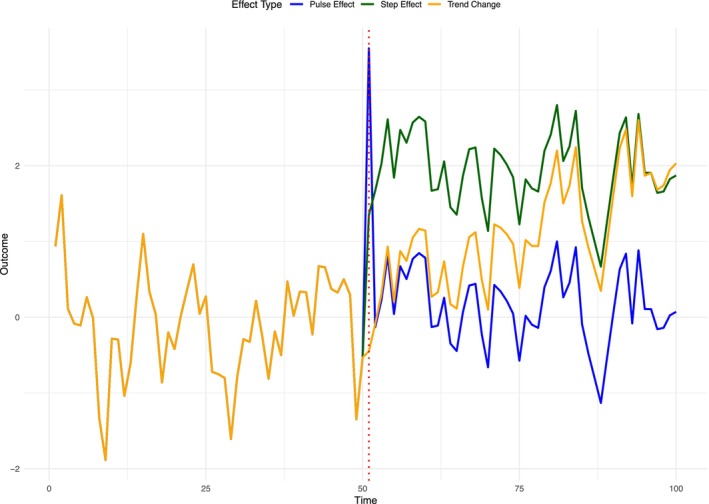
Decomposition of intervention effects on an outcome in interrupted time series (ITS) designs.

Standard analyses for ITS designs with ARIMA models generally include an exogenous regressor, indicating the presence of an intervention. This usually takes one of the three forms shown in Figure 1. The first form, indicated by the green line, is a level variable (or step change), which captures an immediate and sustained change in the outcome level following the intervention. The second form, indicated by the blue line, is a pulse variable that models a temporary change in the outcome level that occurs at the intervention point and then reverts to baseline after a specified number of timepoints. The third form, indicated by the orange line, is a change in trend, where the rate of change in the outcome (the underlying trend) is altered after the intervention.

Zhang *et al*. [21, 22] thus recommend that researchers use power simulation techniques that take these analytical methods into account. The general procedure for power simulations involves running many ITS models on randomly generated data with expected parameter estimates, and calculating the power from the proportion of observations that return results at a given level of statistical significance. This approach is inherently more flexible as researchers can explicitly define the full data‐generating process, including specific autocorrelation error structures (i.e. AR, MA and mixed ARMA orders). Simulations also enable researchers to explore a wide range of parameters (e.g. different covariate effects and intervention timings) that would be intractable with analytical formulas, thus providing a more comprehensive understanding of power across plausible real‐world conditions.

However, Zhang *et al*.’s work has not been widely used in the addictions field. This may be because of their more recent focus on three‐phased ITS analysis. The three‐phased design (with three step‐level changes) includes an initial pre‐intervention phase, an intervention phase and a post‐intervention phase. In fields such as addictions research, health policy and behavioural sciences, researchers often use single‐phase ITS designs (with one step‐level change) to evaluate the impact of a single intervention or policy change, rather than multiple sequential changes. Second, the calculations focused on a small range of effect sizes from pharmaceutical policy literature and only considered segmented AR error models in a generalised least squares framework, which may also restrict its broader use. Moreover, sample size was not examined beyond 108 timepoints and 80% power was not achieved within the tables. Achieving 80% power is often considered a standard in scientific research to ensure that studies can provide reliable and generalisable results [23]. Finally, Zhang *et al*.’s work is also limited by the need for users to possess specialised knowledge to run simulations themselves, reducing its accessibility.

This article addresses these gaps by detailing R code (R Foundation for Statistical Computing, Vienna, Austria) and an accompanying Shiny app [24] to estimate statistical power using simulations for various ITS models with step‐level changes and pulse effects, including those with AR, MA and combined ARMA terms. The code also allows researchers to simulate a change in trend following the intervention. These resources are valuable tools for both prospective study design and retrospective analysis. For prospective planning, researchers can estimate statistical power and determine the required sample size before data collection, which informs the selection of suitable data sets and the optimal timing for the analysis. They can also be used retrospectively with secondary data: by inputting the observed characteristics of an existing data set (such as series length, autocorrelation and noise levels) into the R code or Shiny app, researchers can precisely calculate the minimum effect size their study is powerful enough to detect at a given power level.

To illustrate how researchers can utilise our approach to estimate the power and sample size requirements for their own study, we will use the implementation of policies in addiction research as examples. This article also presents lookup tables, providing a quick reference for researchers needing to estimate sample sizes to detect step‐level changes, pulse effects and changes in underlying trends. This article can be used in conjunction with a primer on time series analysis [20].

## OVERVIEW TO THE POWER CALCULATION TOOLS

Simulation code (available on GitHub) was developed in RStudio 2024.09.0 to allow researchers to thoroughly explore how various factors influence their ability to detect a real intervention effect in ITS studies. At its core, the simulator works by generating synthetic time series data under controlled conditions, then fitting an ARIMA model to these data and, finally, assessing whether the simulated intervention effect is statistically significant. This iterative process, known as Monte Carlo simulation, is repeated many times (as defined by the number of simulations) to provide an estimate of statistical power, which is simply the proportion of simulations where the impact of the intervention is successfully detected (i.e. *P* < 0.05). The entire methodology, from data generation to analysis, is detailed schematically in Appendix S1.

The Shiny app (https://emmabeard.shinyapps.io/my_shiny_app) was designed to significantly enhance the accessibility and ease of use of the power simulation R code. It was created using the ‘Shiny’ R Package developed by Chang *et al*. [24]. Its primary purpose is to enable researchers without direct R programming knowledge to perform complex power calculations through an intuitive, interactive web‐based interface. Although R is the engine behind the app, users do not have to install it (or any other software) and nor do they need to specify any R code. All computations are undertaken in the cloud, accessed by the user through a web browser, and the complex simulations are automatically performed behind the scenes. The application outputs the statistical power to detect the intervention effect, along with informative plots of the simulated data, dynamically generated power curves, and detailed error and warning messages that assist users in troubleshooting problematic parameter combinations. The application comprises a user interface (UI), which specifies the layout and interactive elements for user input, and server logic, which handles these inputs, performs the necessary simulations, calculates the power and renders the outputs.

Both the R code and the Shiny app are designed to be used with aggregated continuous time series data. The first step, is to clearly define the form of your outcome:

- Counts: absolute numbers of events or individuals within a defined period. Examples include daily emergency department visits, weekly confirmed disease cases (e.g. flu, COVID‐19), monthly hospital admissions or annual births.
- Rates: the number of events or cases per specified population size over a defined period, used to standardise counts. Examples include incidence rates (e.g. per 100 000 population), mortality rates or accident and emergency (A&E) attendance per 1000 population.
- Proportions/percentages: the fraction or percentage of a population or subgroup exhibiting a certain characteristic or experiencing an event. Examples include the prevalence of a condition (e.g. smoking prevalence, obesity prevalence) or vaccination uptake percentages.
- Scores/continuous measures: aggregated continuous measurements, typically averages or sums over a time period. Examples include average self‐report craving scores (e.g. on a Likert scale), mean quality‐of‐life scores or average daily air pollution levels.

You must then provide estimates for several key parameters detailed below. These estimates should ideally be derived from existing empirical literature, robust pilot data or informed expert opinion. This approach ensures that the simulations reflect plausible real‐world conditions relevant to the study being planned. Sensitivity analyses are highly recommended to understand how varying these assumptions impacts the final power results.

## STUDY DESIGN PARAMETERS

- Total timepoints (*t*): the total number of observations in your time series, both before and after the intervention. A longer series generally provides greater statistical power. For example, if your data are collected monthly over 3 years, this value would be 36. This value is typically determined by the practical constraints of data availability and the planned duration of data collection for your study.
- Pre‐intervention timepoints (*k*): the number of data points observed before the intervention takes place. This determines the point at which the intervention occurs, being *k* + 1. This value is determined by the specific study design and the timing of the intervention within the overall observation period.
- Time series periodicity: you need to define the ‘frequency’ of your time series. For annual data, use 1; for monthly data, use 12; and for daily data, use 7. This is determined by the temporal granularity of your data collection.
- Baseline intercept: the intercept represents the average baseline value of the outcome variable in the pre‐intervention period, when all other variables are 0. This sets the overall magnitude of your series: if smoking prevalence was 13% at the initial timepoint in the pre‐intervention period, the intercept would be set to 13; similarly, if the average AUDIT score was 8.5 at baseline, the intercept would be 8.5; and if A&E attendance was 15 attendees per 1000 population, the intercept would be 15. The baseline intercept can be directly estimated from historical data or pilot studies.
- Intervention type: choose whether the intervention has a sustained effect (step change), temporary effect (pulse change) or change in slope (change in trend). This selection is based on your hypothesis about the nature of the impact of the intervention.
- Pulse duration (only applicable for pulse change): the number of time points the pulse effect lasts after the intervention point (*k* + 1). For example, a duration of 1 means the effect occurs only at *k* + 1, while a duration of 3 means it affects *k* + 1, *k* + 2 and *k* + 3. This parameter is chosen based on the hypothesised or observed duration of the temporary intervention effect.
- Intervention effect size: this is the effect you are trying to detect and for which the power is calculated. It should reflect a clinically, practically or policy‐relevant change based on prior research, pilot studies or expert consensus.
  - For a step change, it is the magnitude of the immediate, abrupt and sustained change in the level of the dependent variable: if the outcome is smoking prevalence measured as a percentage, a coefficient of 3 signifies a 3‐percentage‐point step increase; for average Alcohol Use Disorders Identification Test (AUDIT) scores, a coefficient of 2.5 indicates a 2.5‐point increase in the average score; and for a rate like A&E attendance per 1000 individuals, a coefficient of −5 denotes an absolute decrease of five attendees per 1000 population.
  - For a pulse change, it is the magnitude of the temporary, abrupt change that occurs starting at the intervention point for the specified ‘pulse duration’: if the outcome is daily calls to a helpline, a coefficient of 10 means an additional 10 calls per day for the duration of the pulse; and if the outcome is hospital admissions for a specific condition, a coefficient of −2 means a temporary reduction of two admissions for the duration of the pulse.
  - For a change in trend, it is the true magnitude of the change in slope after the intervention. For example, if the pre‐intervention trend was an increase of 0.5 units per timepoint, and the intervention caused an effect size of −0.2, the post‐intervention trend would increase by 0.3 units per timepoint. If, instead, the pre‐intervention trend was a decrease of 0.1 units per timepoint and the intervention caused an effect size of 0.3, the post‐intervention trend would become an increase of 0.2 units per timepoint.

## ERROR MODEL (ARIMA) PARAMETERS

The (*p*, *d*, *q*) order specifies the ARIMA model for the residuals (the ‘error’ term after accounting for the intervention, trend and covariates). Estimates are typically derived from preliminary time series analysis of your outcome data, with AR(1) often serving as a common default.

- ARIMA *p*: you can specify the order of the AR component that indicates how many prior observations in the series influence the current observation. You can also specify the numerical value(s) of the AR parameters. These define the strength and direction of the relationship between current errors and past errors. For example, an AR(1) of 0.5 means the current error is influenced by 0.5 times the previous error plus new random noise. This is commonly used as a default if you are unsure. For models to be stationary (i.e. for an assumption to be met), coefficients must lie between −1 and 1 (exclusive).
- ARIMA *d*: the order of differencing (integrated). This specifies the number of times the raw observations are differenced to make the series stationary (i.e. to remove trends or seasonality). If you specify a differencing order (*d* > 0) here, the ARIMA model will automatically difference the outcome variable. A value of 1 can be used when a deterministic trend is specified but note that this would override the choice for modeling a baseline underlying trend. Where no deterministic trend is present, or if you wish to model the baseline underlying trend, this should be set to 0.
- ARIMA *q*: you can specify the order of the MA component. This indicates how many prior forecast errors (residuals) influence the current observation. You can also specify the numerical values(s) of the MA parameters. These define the strength and direction of the relationship between current errors and past forecast errors. For models to be invertible (i.e. for an assumption to be met), coefficients must lie between −1 and 1 (exclusive).
- Noise standard deviation (innovations): you can also specify the standard deviation of the random, uncorrelated noise (innovations) that drives the ARIMA process. A higher value means more random variability, making it harder to detect effects. If you are unsure about the standard deviation for white noise, a common starting point is 1. However, this will be much higher for rate and event data. This should be derived from existing empirical literature on similar outcomes or interventions. If literature is scarce, pilot data are an invaluable resource. This can involve calculating the standard deviation of residuals from preliminary time series models fitted to historical data or a small pre‐intervention sample. Alternatively, using time series decomposition can help isolate the random, uncorrelated noise component from systematic patterns like trend and seasonality.

## OPTIONAL COMPONENTS

### Log transform outcome

You should select this option if effect sizes are on a logarithmic scale. Modelling the logarithm of an outcome variable is a standard approach when dealing with rates (e.g. mortality rates, hospital admissions per 1000 population) or proportions (e.g. smoking prevalence). This is because the effect of an intervention on these outcomes is often better understood as a proportional shift (e.g. a 10% reduction) rather than an absolute shift.

When modelling the logarithm of an outcome variable, it is important to specify all coefficients (intercept, intervention effect, covariate and trend) on the logarithmic scale. For example, if your outcome has a baseline average of 100 on the original scale (e.g. 100 cases per 100 000 population), you would input log(100) (approximately 4.605) for the ‘Baseline Intercept’. For a step‐change intervention, to simulate a 10% increase in the original outcome after the intervention, you would input log(1.10), which is approximately 0.095, for the ‘Intervention Effect Size’. Conversely, to simulate a 5% decrease, you would input log(0.95), which is approximately −0.051.

In the case of a pulse‐change intervention, if a temporary 15% increase in the outcome (e.g. daily calls to a helpline) is expected, you would input log(1.15), or approximately 0.140, for the ‘Intervention Effect Size’. If an 8% temporary reduction in the outcome (e.g. hospital admissions for a specific condition) is anticipated, you would input log(0.92), approximately −0.083. Finally, for a change‐in‐trend intervention, consider an outcome previously decreasing by 2% per timepoint. This would have an existing trend slope of log(0.98). If the intervention is expected to flatten this to no change (0% growth or decay), you would input approximately 0.0202 for the ‘Intervention Effect Size’ [calculated as log(1.00) − log(0.98) = 0 − (−0.0202) = 0.0202]. It is important to remember that when you model log(outcome), a coefficient *β* on the log scale signifies a multiplicative effect on the original scale equivalent to exp(*β*). Therefore, a *β* of 0.095 translates to exp(0.095) ≈ 1.10, indicating a 10% increase.

### Data‐generating process (DGP) and model specification

These settings allow you to simulate data with or without a covariate/time trend to understand the impact of model misspecification.

- Covariate: in the real world, your outcome is often affected by more than just your intervention. A time‐varying covariate (or confounder) is an external variable that also changes over time and could affect your outcome. By adding a covariate to your simulation, you can realistically estimate your statistical power to detect the true effect of the intervention, even in the presence of this competing influence. The simulator generates this covariate from a normal distribution and models it as a standard time series [specifically, an AR(1) process with *ρ* = 0.5] to mimic typical real‐world data. You can also simulate a scenario where the intervention itself causes a shift in the baseline level of the covariate. This is crucial for exploring complex situations, such as when the intervention itself affects the confounder, or when a separate external event causes a change at the same time. Crucially, you can choose whether to include this simulated covariate in the final statistical model used for the power analysis. This feature lets you directly compare two critical scenarios:
  ○ Correctly specified model: you measure the confounder and include it in your analysis.
  ○ Omitted variable bias: the confounder exists and affects your outcome, but you exclude it from your model.
    By comparing the statistical power between these scenarios, you can understand the risk of drawing incorrect conclusions if a key confounder is ignored.
- Time trend: correctly handling the underlying trend in your time series is essential for an accurate power analysis. Our tool helps you simulate a trend in your data and then test how well your chosen statistical model can account for it. First, you need to specify the nature of the trend you believe exists in your data. There are two main types:
  ○ Deterministic trend: this is a trend that can be expressed as a predictable, non‐random function of time (e.g. a straight line). If a deterministic trend exists in the DGP, it means the series has a constant average rate of increase or decrease over time. For example, monthly rates of youth smoking might show a consistent, long‐term decrease of 0.1 percentage points each month owing to ongoing, universal school prevention programmes and media campaigns.
  ○ Stochastic trend: a trend that is not predictable and changes randomly over time. It is often represented by a random walk or an integrated process, meaning that the current value is the previous value plus a random shock. If a stochastic trend exists in the DGP, it means that past random shocks have a permanent impact on the series level. For example, the weekly number of overdose emergency calls could exhibit a stochastic trend, where random events like a batch of highly potent illicit drugs or a local community intervention have a lasting, unpredictable impact that permanently shifts the baseline number of calls up or down.

Next, you will need to specify the strength of the trend that you have just defined. For deterministic trends, this factor functions as the slope, quantifying the consistent change in the series per unit of time. For stochastic trends, it acts as a scaling factor applied to the cumulative sum of random innovations, thereby influencing the overall volatility and magnitude of the random walk. While a value of 1 means the raw stochastic trend is added directly (and should be used as the default), a value of 0 effectively removes its contribution; other positive values can amplify (e.g. 2.0) or dampen (e.g. 0.5) its effect.

Finally, you must decide how the statistical model in your power analysis will attempt to account for the trend that you have simulated in the data. This is a crucial step for understanding potential biases.

- Do not model: the trend component is entirely excluded from the regression model. This option allows for investigating scenarios where a trend is ignored during analysis.
- Model as exogenous regressor: a linear time trend variable (e.g. *t* = 1, 2, …, *T*) is explicitly added as a predictor in the ARIMA model. This approach is generally appropriate for accounting for deterministic trends.
- Model through differencing (*d* = 1): the dependent variable is differenced once. This operation effectively removes linear trends (both deterministic and stochastic trends that are integrated processes). When this option is selected, the ‘*d*’ parameter in the ARIMA order is set to 1. It is important to note that when differencing is applied, a direct coefficient estimate for ‘time’ is not produced in the model output, as the trend is implicitly removed by the differencing process.

## SIMULATION SETTINGS

These control the simulation process itself.

- Number of simulations: the total number of individual time series data sets that will be generated and analysed. More simulations provide a more accurate estimate of power but take longer to run.
- Random seed: a number used to initialise the random number generator. Setting a seed ensures that your simulations are reproducible; running with the same seed will yield the exact same results.

## EXAMPLES

Below are three examples of how the R code and Shiny app can be used to detect: a step change (Example 1); a change in trend (Example 2); and a pulse effect (Example 3). These examples come from areas within addiction research.

### Example 1, plain packaging for tobacco products: step‐level change

This example forms the default settings for the Shiny app. In May 2016, England introduced standardised packaging legislation for cigarettes. After a 1‐year transition period to May 2017, tobacco products could be sold in England only if packaged in generic dark‐green packs with brand names and a single descriptor presented in a standard font. These requirements were implemented alongside the 2014 European Tobacco Products Directive, which among other measures, mandated minimum pack sizes and larger pictorial health warnings [25]. For this scenario, we are interested in the impact on the prevalence of quit attempts and assume a step‐level permanent change.

The default parameter values for our simulation were derived from the Smoking Toolkit Study (STS) [26, 27, 28]. The STS is a monthly household survey of adults aged 18 years and over, utilising a combination of face‐to‐face interviews and online questionnaires to collect comprehensive data on smoking prevalence, cessation attempts, the use of smoking cessation aids, and related attitudes and behaviours. The total series length was set to 200 waves (e.g. months), with the intervention positioned at wave 100. The baseline quit rate, serving as the intercept value, was set to 36%. The inherent variability or ‘noise’ in the outcome, representing unexplained variance captured by the standard deviation of the error term, was set to 1, with an AR(1) = 0.5 error model. An underlying deterministic linear trend of −0.13 was introduced and a time‐varying confounder, with its beta coefficient representing the true effect on the outcome, was set to 0.098 [28]. The baseline mean value for the confounder was 10% with a standard deviation of 3%. To establish its confounding relationship with the intervention, a shift of one percentage point was applied to the confounder level immediately after the intervention occurred. Both the trend and the covariate were modelled in the simulations.

**FIGURE 2:**
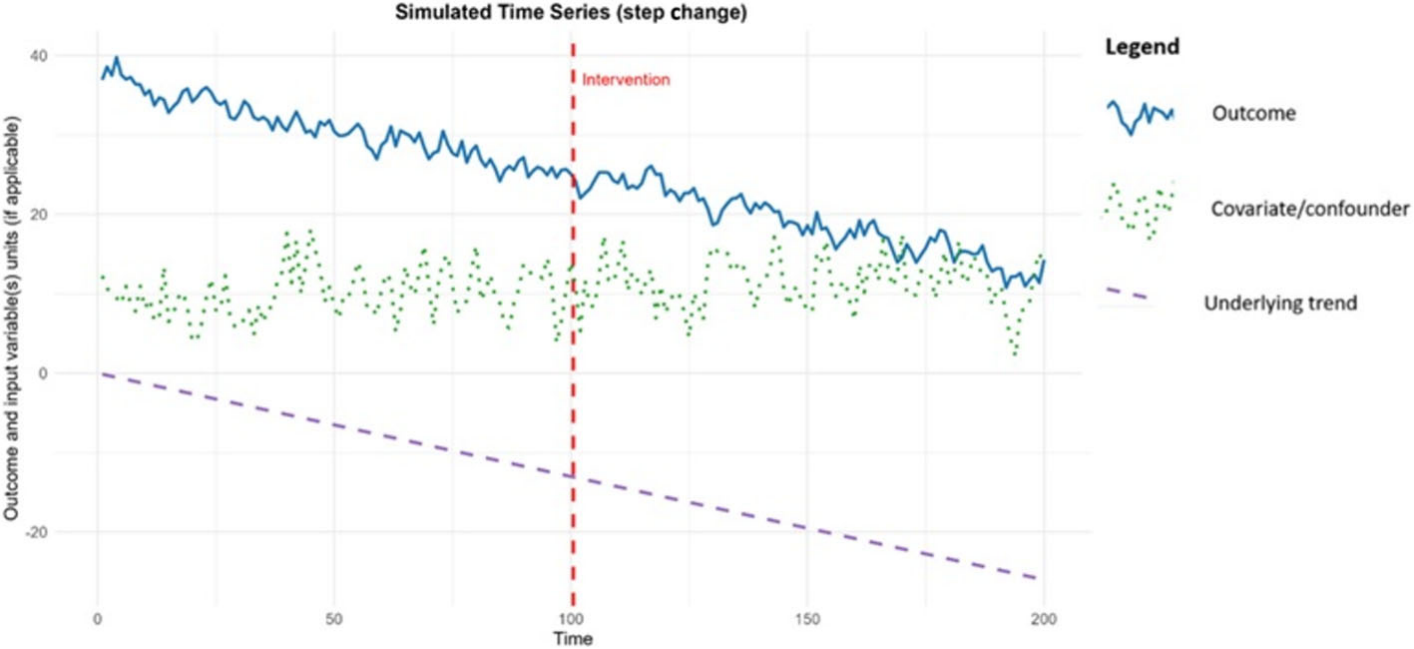
Example simulated data for the illustrative example of the introduction of plain packaging for tobacco products in England.

**FIGURE 3:**
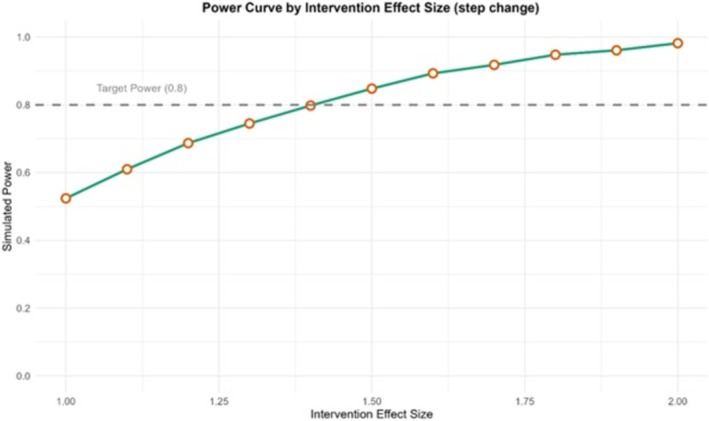
Example power curve for the illustrative example of the introduction of plain packaging of tobacco products in England.

It was found that a step change of 1.40 percentage points could be identified with 80% power. This is within the range of meaningful impacts that the plain packaging legislation is hypothesised and observed to have on tobacco use outcomes [16, 29]. An example of the simulated data is given in Figure 2, showing the outcome and any input variables. Figure 3 is a power curve for this scenario showing how the statistical power changes as the intervention effect size varies, holding all other parameters constant. These graphs are produced within the Shiny app and can be downloaded.

The function for the R code is shown below and the configuration of the Shiny app is presented in Figure S1 of Appendix S5.


its_power_simulator(t=200, errormodel = list (order = c(1, 0, 0), ar = 0.5, ma = NULL), period = 12, k=100, b0 = 36, b1_effect = 1.40, intervention_type = "step", pulse_duration = 0, b2_covariate = 0.098, b2_mean = 10, b2_sd = 3, b2_shift_after_k = 1, noise_sd = 1, b3_trend = â 0.13, include_covariate = TRUE, model_covariate = TRUE, include_trend = TRUE, trend_model_method = "xreg", trend_type = "deterministic", stochastic_trend_sd = 0, log_transform_outcome = FALSE, seed = 123, n.sims=1000)




### Example 2, minimum unit pricing for alcohol: change in trend

In May 2018, minimum unit pricing (MUP) was established for alcohol in Scotland. MUP stipulates a floor price below which alcohol cannot be sold, with the primary aim of reducing alcohol consumption, especially of cheaper, higher‐strength products, and subsequently diminishing alcohol‐related harm. For this scenario we will focus on its expected impact on wholly alcohol‐attributable hospital admissions [3].

The default settings mirror the conditions for the evaluation of MUP, using publicly available monthly hospital admissions data from Scotland [3, 30, 31]. The total series length is set to 180 months, spanning 15 years. As the MUP intervention was introduced in May 2018, this gives 160 pre‐intervention timepoints. As the data is collected monthly, the time series periodicity is 12. The baseline intercept, representing the average pre‐intervention alcohol‐specific hospital admission rate, is set to 650 per 100 000 population, aligning with the observed rates before MUP. It is hypothesised that subsequent behavioural changes (e.g. shifts in purchasing habits, reduced consumption) and the resulting health improvements (like a decrease in alcohol‐attributable hospital admissions or deaths) may not occur instantaneously. Instead, these changes often unfold gradually over time as individuals and the broader population adjust to the new pricing structure. This gradual, ongoing shift in the rate of change is characteristic of a trend alteration. The inherent variability or noise in the outcome, representing unexplained variance captured by the standard deviation of the error term, was set to 20, with an AR(1) = 0.5 error model. We incorporate a time‐varying confounder: unemployment rate. The covariate coefficient is 5.0, suggesting that a single percentage point increase in unemployment leads to five more admissions per 100 000. The unemployment rate itself is simulated with a mean of 5% and a standard deviation of 1.0, and we assume no immediate shift after the intervention. Furthermore, we account for underlying baseline deterministic trends in hospital admissions. The baseline trend magnitude factor is −0.5, representing a slight, gradual decrease of 0.5 admissions per month. Both the trend and covariate were modelled in the simulations.

**FIGURE 4:**
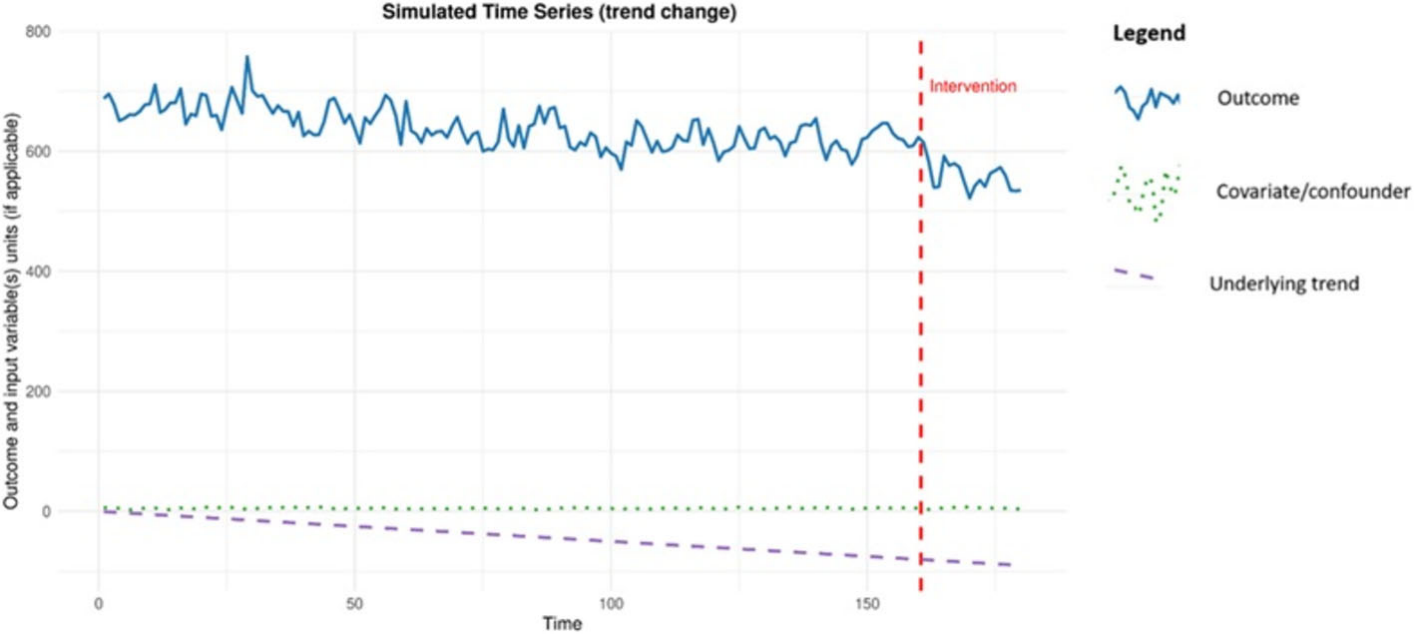
Example simulated data for the illustrative example of the introduction of minimum unit pricing (MUP) in Scotland.

**FIGURE 5:**
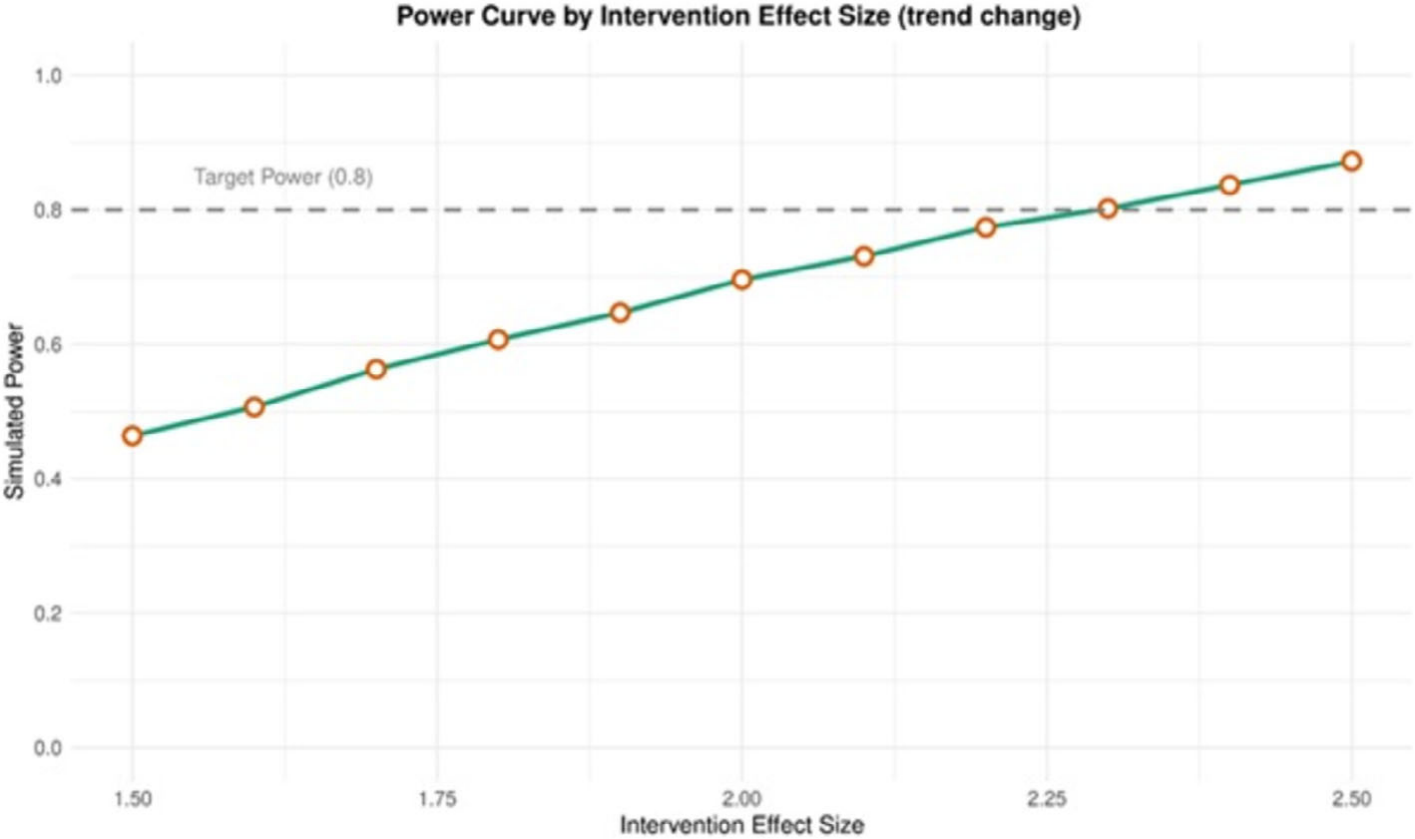
Example power curve for the illustrative example of minimum unit pricing (MUP) in Scotland.

It was found that a reduction of 2.3 alcohol‐attributable hospital admissions (per 100 000 population) each month could be identified with 80% power. This magnitude of reduction is consistent with the observed and hypothesised impacts of MPU legislation on alcohol‐related harm outcomes. A 4.1% reduction in hospital admissions has been attributed to alcohol consumption following MUP implementation, which translates to approximately −1.3 admissions per 100 000 population per month, assuming a baseline of 650 alcohol‐attributable hospital admissions (per 100 000 population) [30]. An example of the simulated data is given in Figure 4, showing the outcome and any input variables. Figure 5 is a power curve for this scenario showing how the statistical power changes as the intervention effect size varies, holding all other parameters constant. These graphs are produced within the Shiny app and can be downloaded.

The function for the R code is shown below and the configuration of the Shiny app is presented in Figure S2 of Appendix S5.


its_power_simulator(t=180, errormodel = list (order = c(1, 0, 0), ar = 0.5, ma = NULL), period = 12, k=160, b0 = 650, b1_effect = â 2.3, intervention_type = "trend_change", pulse_duration = 0, b2_covariate = 5, b2_mean = 5, b2_sd = 1, b2_shift_after_k = 0, noise_sd = 20, b3_trend = â 0.5, include_covariate = TRUE, model_covariate = TRUE, include_trend = TRUE, trend_model_method = "xreg", trend_type = "deterministic", stochastic_trend_sd = 0, log_transform_outcome = FALSE, seed = 123, n.sims=1000)




### Example 3, National Prescription Drug Take‐Back Day: pulse effect

In October 2019, the US Drug Enforcement Administration (DEA) held its biannual ‘National Prescription Drug Take‐Back Day’, a 1‐day event held across the country [32, 33, 34]. This initiative provides a safe, convenient, and responsible means of disposing of unneeded and expired prescription drugs, particularly opioids, from household medicine cabinets. The event was heavily advertised through local law enforcement, community centres and media outlets for a few days leading up to the designated Saturday. After this single collection day, the special take‐back sites were closed, although some permanent disposal options remained available. For this scenario, we will focus on its expected impact on the daily volume of prescription opioids collected via designated take‐back sites in a specific state.

The total series length is set to 200 days, encompassing a period of roughly 6 months. As the intervention, the take‐back day, was a single‐day event, we will consider the pre‐intervention timepoints as 100 days leading up to the event. As the data are collected daily, the time series periodicity is 7 (for a weekly pattern, although the event itself is singular). The baseline intercept, representing the average daily collection volume at routine, permanent sites in the pre‐intervention period, is set to 5 kg of opioids, reflecting a typical ongoing collection rate.

It is hypothesised that this highly publicised, single ‘National Prescription Drug Take‐Back Day’ would lead to an immediate and significant spike in the volume of prescription opioids collected only on that specific day. As the special event concludes, the collection volume would then be expected to rapidly return to its pre‐intervention daily baseline levels from the more routine disposal methods. The inherent variability or noise in the outcome was set to 2, with an AR(1) = 0.3 error model. We assume no underlying deterministic trends or time‐varying confounders are included.

**FIGURE 6:**
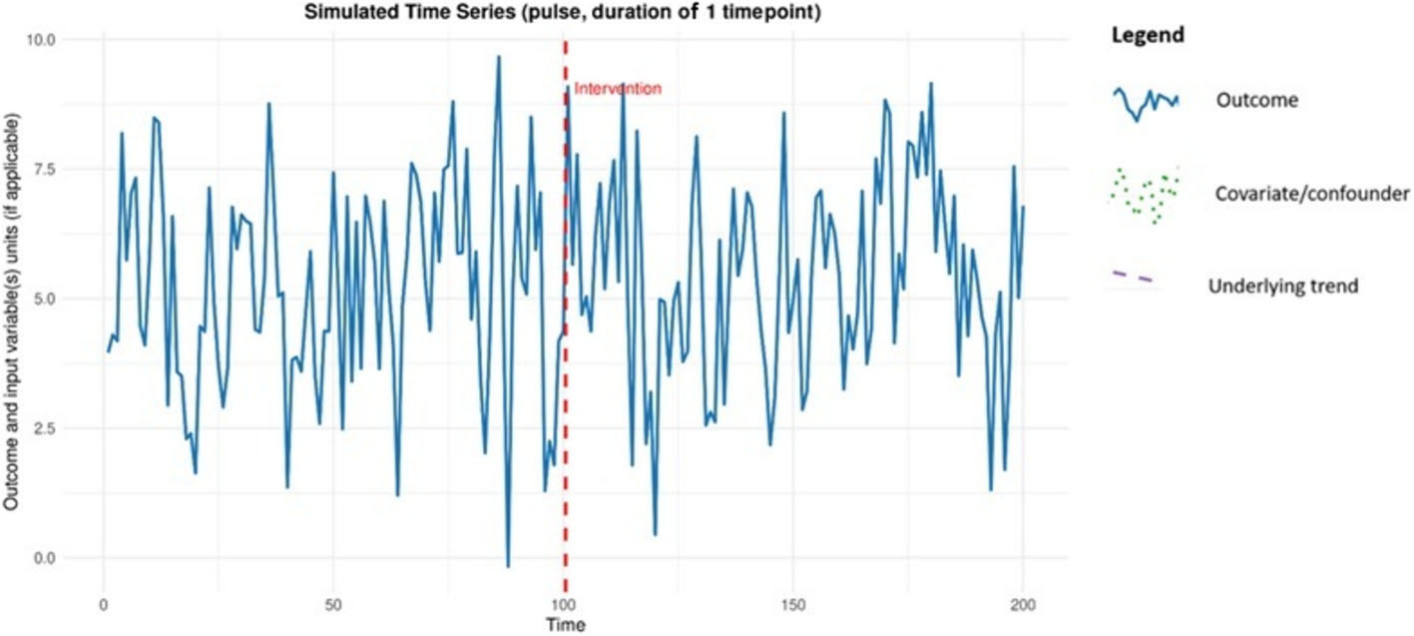
Example simulated data for the illustrative example of the National Prescription Drug Take‐Back Day.

**FIGURE 7:**
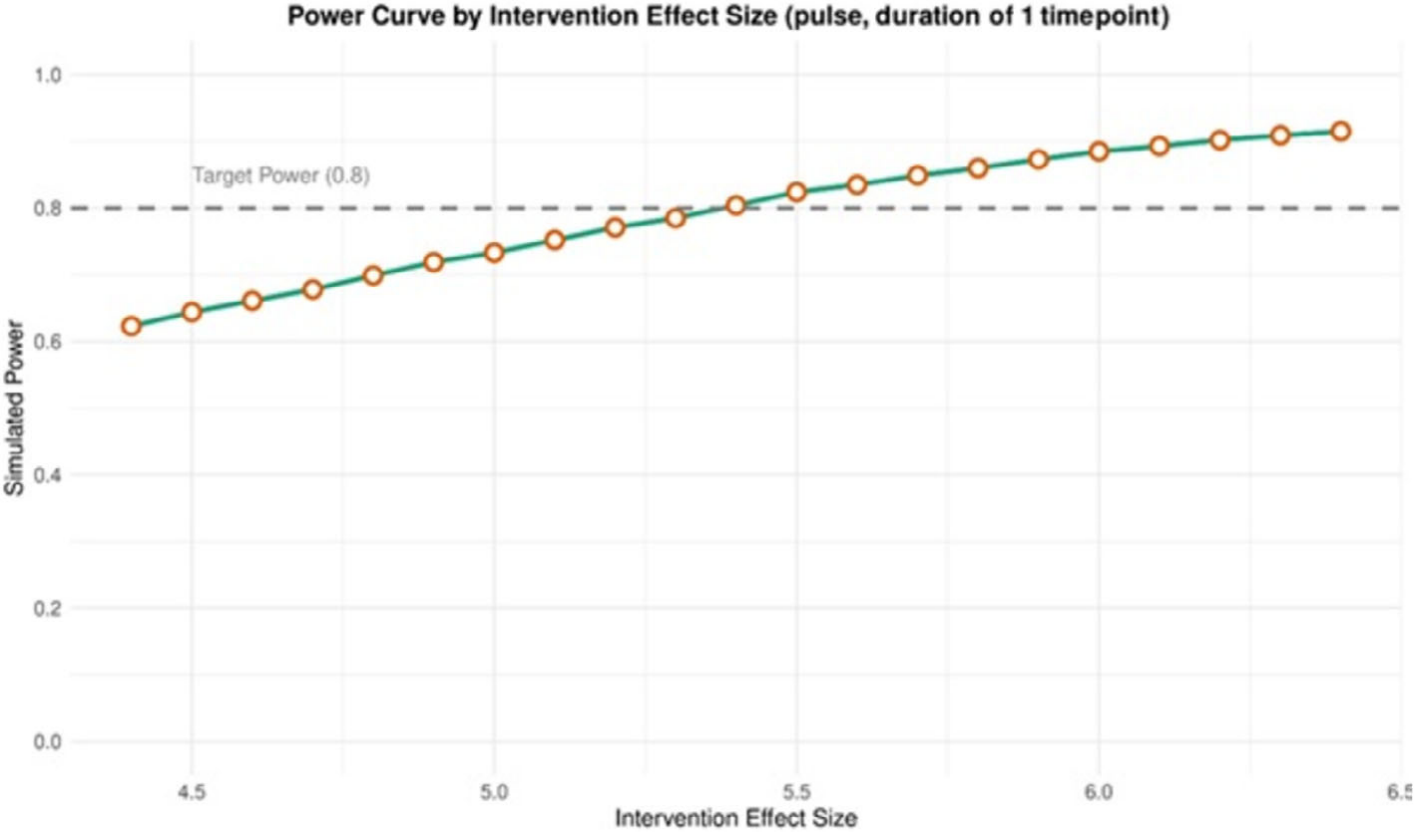
Example power curve for the illustrative example of the National Prescription Drug Take‐Back Day.

It was found that an immediate increase of 5.4 kg in opioids collected on the day of the event could be identified with 80% power. An example of the simulated data is given in Figure 6, showing the outcome. Figure 7 is a power curve for this scenario showing how the statistical power changes as the intervention effect size varies, holding all other parameters constant. These graphs are produced within the Shiny app and can be downloaded.

The function for the R code is shown below and the configuration of the Shiny app is presented in Figure S3 of Appendix S5.


its_power_simulator(t=200, errormodel = list (order = c(1, 0, 0), ar = 0.3, ma = NULL), period = 7, k=100, b0 = 5, b1_effect = 5.4, intervention_type = "pulse", pulse_duration = 1, b2_covariate = 0, b2_mean = 0, b2_sd = 0, b2_shift_after_k = 0, noise_sd = 2, b3_trend = 0, include_covariate = FALSE, model_covariate = FALSE, include_trend = FALSE, trend_model_method = "xreg", trend_type = "deterministic", stochastic_trend_sd = 0, log_transform_outcome = FALSE, seed = 123, n.sims=1000)




### LOOKUP TABLES

To provide researchers with a readily accessible and generalisable resource, we generated a series of power lookup tables using the simulation code. These tables serve as a quick reference, enabling researchers to estimate the statistical power of their ITS designs under various common conditions. For consistency and direct comparability with prior foundational work in ITS power analysis, particularly the simulations conducted by Zhang *et al*. (2018), we standardised several key parameters. The baseline probability of the outcome (intercept) was consistently set at 0 and the intervention was uniformly positioned at the midpoint of the simulated time series. This study systematically varied the following critical parameters to construct the lookup tables:

- Duration of data collection (total timepoints): the length of the time series was systematically varied from 20 to 400 timepoints. A minimum of 20 timepoints was established as a lower bound, as simulations with fewer observations consistently led to issues with non‐stationary autocorrelation coefficients.
- Intervention effect: we considered: (i) step effects, which represent an immediate and sustained change in the outcome level following the intervention; (ii) pulse effects, which represent a temporary change that occurs at the intervention point and then reverts to baseline; and (iii) slope changes, which are modifications to the outcome rate of change.
- Intervention effect size: the magnitude of the immediate impact of the intervention was varied across a range. For step changes from 0.1 to 3.0, for pulse effects from 1.1 to 4.0 and for changes in trend from 0.01 to 0.3. This spectrum was deliberately chosen to encompass a broad array of potential intervention effects in real‐world settings. This includes small, subtle changes that might signify early, incremental policy effectiveness or interventions with limited initial reach, as well as large, clinically or practically significant shifts that would indicate a substantial and immediate alteration in the outcome. For a step effect, this represents the immediate and sustained impact. For a pulse effect, this represents the magnitude of the temporary change in the outcome that occurs at the intervention point and lasts for a specified duration. For a change in slope, this represents the magnitude of the alteration to the trend occurring at the intervention point. For the purpose of these lookup tables, pulse effects were consistently simulated as lasting for a single timepoint (i.e. pulse duration = 1). For a change in trend, an underlying deterministic trend was also modelled assuming an associated coefficient of 0 during the pre‐intervention period.
- Standard deviation of white noise: this parameter, representing the inherent variability and randomness in the data not accounted for by systematic components, was explored across values ranging from 0.5 to 5.0. A higher standard deviation signifies increased variability and ‘noise’ in the series. Investigating this range is critical as it directly mimics real‐world scenarios characterised by high data variability and unpredictability [26, 28], which can significantly obscure underlying patterns or signals of an intervention effect.
- Level of AR(1) autocorrelation: the degree to which each data point is correlated with its immediate predecessor was systematically varied between 0.1 and 0.9. AR(1) is a common feature of time series data, and its magnitude significantly influences the precision of parameter estimates in ITS analysis. By varying this parameter, we investigated how different levels of serial dependency impact the power to detect an intervention effect. We focused solely on AR(1) models because of their prevalence in public health and behavioural sciences research. In these fields, data often exhibit a direct dependency on the immediately preceding observation, and an AR(1) model frequently provides an adequate and parsimonious representation of the observed temporal dependency, allowing for a balance between model complexity and explanatory power [35].

It is crucial to note that while versatile, these lookup tables are generated with a fixed standard deviation of white noise (ranging from 0.5 to 5.0). Consequently, they may be most directly applicable to outcomes expressed as percentages/proportions or scores, such as a 3‐percentage‐point step increase in alcohol consumption prevalence or a 2.5‐point increase in self‐reported cravings measured on a 5‐point Likert scale. They might not accurately reflect the power for rates or event data that inherently exhibit much higher noise levels, such as A&E attendance per 1000 population or hospital admissions. The diverse and often high inherent variability in noise across different types and magnitudes of rates and event data makes comprehensive lookup tables unfeasible for this broad range. For outcomes with variable or high noise, researchers are strongly recommended to utilise the R code or the Shiny app, which offer greater flexibility in specifying noise levels to match their specific data characteristics.

Tables 1, 2 and 3 detail the statistical power to detect a step‐level change, pulse effect and change in trend under varying conditions, for an AR(1) = 0.5 error model with standard deviation of white noise equal to 1. Specifically, they examine the impact of time series length and effect size. The simulations assumed no underlying trend or covariates needing adjustment, and the intervention occurred at the midpoint of the time series. In general, for very small effect sizes, the power to detect a change is consistently low and exhibits some instability. This highlights a fundamental limitation, that even with extended time series, a truly minute effect might be difficult to reliably detect if it is overshadowed by inherent data variability.

**TABLE 1:** Power to detect a step‐level change as a function of time and size of the step‐level change, with AR = 0.5 autocorrelation and a standard deviation of 1 for white noise.

Time	Effect size for the step level																													
	0.1	0.2	0.3	0.4	0.5	0.6	0.7	0.8	0.9	1.0	1.1	1.2	1.3	1.4	1.5	1.6	1.7	1.8	1.9	2.0	2.1	2.2	2.3	2.4	2.5	2.6	2.7	2.8	2.9	3.0
40	0.17	0.18	0.20	0.22	0.24	0.27	0.30	0.35	0.40	0.45	0.49	0.53	0.59	0.63	0.68	0.71	0.76	0.80	0.82	0.85	0.89	0.90	0.92	0.93	0.95	0.96	0.96	0.97	0.98	0.98
60	0.14	0.15	0.18	0.21	0.25	0.29	0.33	0.37	0.44	0.50	0.58	0.64	0.69	0.75	0.80	0.84	0.88	0.91	0.94	0.95	0.97	0.98	0.98	0.99	0.99	0.99	0.99	1.00	1.00	1.00
80	0.09	0.11	0.15	0.20	0.27	0.34	0.41	0.49	0.57	0.64	0.72	0.78	0.82	0.87	0.89	0.93	0.96	0.97	0.98	0.98	0.99	0.99	1.00	1.00	1.00	1.00	1.00	1.00	1.00	1.00
100	0.08	0.10	0.15	0.21	0.27	0.35	0.43	0.51	0.61	0.70	0.78	0.83	0.89	0.93	0.95	0.98	0.98	0.99	0.99	1.00	1.00	1.00	1.00	1.00	1.00	1.00	1.00	1.00	1.00	1.00
120	0.08	0.10	0.15	0.21	0.29	0.41	0.51	0.61	0.71	0.79	0.85	0.90	0.94	0.97	0.98	0.99	0.99	1.00	1.00	1.00	1.00	1.00	1.00	1.00	1.00	1.00	1.00	1.00	1.00	1.00
140	0.08	0.11	0.17	0.25	0.34	0.44	0.56	0.67	0.77	0.84	0.90	0.94	0.96	0.98	0.99	1.00	1.00	1.00	1.00	1.00	1.00	1.00	1.00	1.00	1.00	1.00	1.00	1.00	1.00	1.00
160	0.10	0.15	0.21	0.29	0.39	0.50	0.62	0.72	0.81	0.88	0.93	0.96	0.98	0.99	0.99	1.00	1.00	1.00	1.00	1.00	1.00	1.00	1.00	1.00	1.00	1.00	1.00	1.00	1.00	1.00
180	0.08	0.14	0.21	0.30	0.42	0.53	0.66	0.76	0.86	0.91	0.96	0.98	0.99	0.99	1.00	1.00	1.00	1.00	1.00	1.00	1.00	1.00	1.00	1.00	1.00	1.00	1.00	1.00	1.00	1.00
200	0.06	0.12	0.20	0.31	0.45	0.57	0.70	0.80	0.89	0.94	0.97	0.99	1.00	1.00	1.00	1.00	1.00	1.00	1.00	1.00	1.00	1.00	1.00	1.00	1.00	1.00	1.00	1.00	1.00	1.00
220	0.09	0.14	0.20	0.33	0.47	0.61	0.74	0.83	0.90	0.95	0.98	0.99	1.00	1.00	1.00	1.00	1.00	1.00	1.00	1.00	1.00	1.00	1.00	1.00	1.00	1.00	1.00	1.00	1.00	1.00
240	0.08	0.15	0.23	0.36	0.49	0.64	0.78	0.87	0.93	0.97	0.99	0.99	1.00	1.00	1.00	1.00	1.00	1.00	1.00	1.00	1.00	1.00	1.00	1.00	1.00	1.00	1.00	1.00	1.00	1.00
260	0.08	0.13	0.23	0.36	0.53	0.69	0.79	0.88	0.94	0.97	0.99	1.00	1.00	1.00	1.00	1.00	1.00	1.00	1.00	1.00	1.00	1.00	1.00	1.00	1.00	1.00	1.00	1.00	1.00	1.00
280	0.09	0.16	0.26	0.41	0.56	0.69	0.83	0.91	0.96	0.98	0.99	1.00	1.00	1.00	1.00	1.00	1.00	1.00	1.00	1.00	1.00	1.00	1.00	1.00	1.00	1.00	1.00	1.00	1.00	1.00
300	0.09	0.16	0.27	0.41	0.59	0.74	0.86	0.93	0.97	0.99	1.00	1.00	1.00	1.00	1.00	1.00	1.00	1.00	1.00	1.00	1.00	1.00	1.00	1.00	1.00	1.00	1.00	1.00	1.00	1.00
320	0.08	0.17	0.30	0.45	0.60	0.77	0.88	0.94	0.98	0.99	1.00	1.00	1.00	1.00	1.00	1.00	1.00	1.00	1.00	1.00	1.00	1.00	1.00	1.00	1.00	1.00	1.00	1.00	1.00	1.00
340	0.08	0.17	0.31	0.47	0.62	0.81	0.91	0.96	0.99	1.00	1.00	1.00	1.00	1.00	1.00	1.00	1.00	1.00	1.00	1.00	1.00	1.00	1.00	1.00	1.00	1.00	1.00	1.00	1.00	1.00
360	0.08	0.16	0.29	0.48	0.64	0.80	0.91	0.97	0.99	1.00	1.00	1.00	1.00	1.00	1.00	1.00	1.00	1.00	1.00	1.00	1.00	1.00	1.00	1.00	1.00	1.00	1.00	1.00	1.00	1.00
380	0.07	0.18	0.31	0.50	0.67	0.81	0.92	0.97	0.99	1.00	1.00	1.00	1.00	1.00	1.00	1.00	1.00	1.00	1.00	1.00	1.00	1.00	1.00	1.00	1.00	1.00	1.00	1.00	1.00	1.00
400	0.09	0.20	0.33	0.51	0.71	0.83	0.92	0.97	0.99	1.00	1.00	1.00	1.00	1.00	1.00	1.00	1.00	1.00	1.00	1.00	1.00	1.00	1.00	1.00	1.00	1.00	1.00	1.00	1.00	1.00

*Note*: In the simulations it was assumed that there was no underlying trend or covariates that needed to be adjusted for. The intervention was assumed to have occurred at the midpoint of the time series.

**TABLE 2:** Power to detect a pulse effect as a function of time and size of the pulse effect, with AR = 0.5 autocorrelation and a standard deviation of 1 for white noise.

Time	Effect size pulse effect																													
	1.1	1.2	1.3	1.4	1.5	1.6	1.7	1.8	1.9	2.0	2.1	2.2	2.3	2.4	2.5	2.6	2.7	2.8	2.9	3.0	3.1	3.2	3.3	3.4	3.5	3.6	3.7	3.8	3.9	4.0
40	0.26	0.28	0.31	0.34	0.39	0.44	0.49	0.53	0.58	0.62	0.67	0.72	0.74	0.77	0.93	0.94	0.95	0.96	0.97	0.97	0.98	0.98	0.98	0.99	0.93	0.94	0.95	0.96	0.97	0.97
60	0.26	0.30	0.33	0.37	0.41	0.45	0.49	0.53	0.58	0.63	0.67	0.71	0.75	0.77	0.94	0.94	0.96	0.96	0.97	0.98	0.98	0.99	0.99	0.99	0.94	0.94	0.96	0.96	0.97	0.98
80	0.23	0.27	0.30	0.34	0.38	0.43	0.48	0.52	0.56	0.61	0.65	0.68	0.71	0.75	0.93	0.94	0.95	0.96	0.97	0.97	0.98	0.99	0.99	1.00	0.93	0.94	0.95	0.96	0.97	0.97
100	0.23	0.26	0.32	0.35	0.38	0.42	0.47	0.51	0.56	0.61	0.64	0.69	0.72	0.76	0.94	0.95	0.96	0.96	0.97	0.98	0.99	0.99	0.99	0.99	0.94	0.95	0.96	0.96	0.97	0.98
120	0.24	0.28	0.32	0.35	0.38	0.42	0.49	0.53	0.58	0.62	0.66	0.71	0.75	0.79	0.95	0.96	0.96	0.97	0.98	0.99	0.99	0.99	0.99	1.00	0.95	0.96	0.96	0.97	0.98	0.99
140	0.26	0.29	0.32	0.36	0.39	0.43	0.49	0.52	0.56	0.61	0.65	0.69	0.74	0.77	0.93	0.94	0.95	0.96	0.97	0.97	0.98	0.98	0.99	0.99	0.93	0.94	0.95	0.96	0.97	0.97
160	0.23	0.27	0.30	0.34	0.38	0.43	0.49	0.52	0.58	0.62	0.66	0.70	0.73	0.78	0.94	0.96	0.97	0.98	0.98	0.99	0.99	0.99	0.99	0.99	0.94	0.96	0.97	0.98	0.98	0.99
180	0.24	0.29	0.34	0.38	0.42	0.45	0.50	0.53	0.57	0.62	0.66	0.70	0.73	0.77	0.94	0.95	0.96	0.97	0.97	0.98	0.98	0.99	0.99	0.99	0.94	0.95	0.96	0.97	0.97	0.98
200	0.23	0.26	0.31	0.36	0.41	0.45	0.51	0.55	0.59	0.64	0.68	0.71	0.75	0.79	0.95	0.96	0.96	0.97	0.98	0.98	0.99	0.99	0.99	0.99	0.95	0.96	0.96	0.97	0.98	0.98
220	0.21	0.25	0.29	0.34	0.38	0.43	0.47	0.52	0.56	0.60	0.64	0.68	0.72	0.76	0.93	0.95	0.96	0.97	0.98	0.99	0.99	0.99	1.00	1.00	0.93	0.95	0.96	0.97	0.98	0.99
240	0.24	0.27	0.31	0.34	0.38	0.43	0.47	0.51	0.55	0.62	0.66	0.70	0.73	0.77	0.93	0.94	0.95	0.97	0.98	0.98	0.99	0.99	0.99	0.99	0.93	0.94	0.95	0.97	0.98	0.98
260	0.26	0.29	0.33	0.36	0.40	0.44	0.49	0.53	0.57	0.61	0.66	0.69	0.72	0.75	0.93	0.94	0.95	0.96	0.97	0.98	0.98	0.99	0.99	0.99	0.93	0.94	0.95	0.96	0.97	0.98
280	0.24	0.29	0.31	0.35	0.39	0.43	0.46	0.51	0.56	0.61	0.64	0.68	0.71	0.74	0.94	0.95	0.96	0.96	0.97	0.98	0.98	0.99	0.99	0.99	0.94	0.95	0.96	0.96	0.97	0.98
300	0.25	0.28	0.32	0.36	0.41	0.45	0.51	0.55	0.59	0.63	0.67	0.71	0.75	0.79	0.94	0.96	0.97	0.97	0.98	0.99	0.99	0.99	1.00	1.00	0.94	0.96	0.97	0.97	0.98	0.99
320	0.23	0.27	0.30	0.34	0.38	0.43	0.49	0.53	0.58	0.63	0.67	0.71	0.75	0.78	0.93	0.95	0.96	0.97	0.98	0.98	0.99	0.99	0.99	0.99	0.93	0.95	0.96	0.97	0.98	0.98
340	0.25	0.28	0.31	0.36	0.40	0.44	0.49	0.53	0.57	0.62	0.67	0.71	0.75	0.78	0.94	0.95	0.96	0.97	0.98	0.98	0.99	0.99	0.99	0.99	0.94	0.95	0.96	0.97	0.98	0.98
360	0.24	0.28	0.32	0.37	0.41	0.46	0.50	0.55	0.59	0.64	0.68	0.71	0.74	0.77	0.94	0.95	0.96	0.97	0.97	0.98	0.99	0.99	0.99	1.00	0.94	0.95	0.96	0.97	0.97	0.98
380	0.22	0.24	0.28	0.31	0.35	0.39	0.44	0.50	0.54	0.58	0.63	0.67	0.71	0.75	0.92	0.94	0.96	0.96	0.97	0.98	0.98	0.98	0.99	0.99	0.92	0.94	0.96	0.96	0.97	0.98
400	0.24	0.27	0.32	0.37	0.42	0.45	0.49	0.53	0.58	0.62	0.66	0.71	0.74	0.77	0.94	0.95	0.96	0.97	0.98	0.98	0.99	0.99	0.99	1.00	0.94	0.95	0.96	0.97	0.98	0.98

*Note*: In the simulations it was assumed that there was no underlying trend or covariates that needed to be adjusted for. The intervention was assumed to have occurred at the midpoint of the time series.

**TABLE 3:** Power to detect a change in trend as a function of time and size of change in trend, with AR = 0.5 autocorrelation and a standard deviation of 1 for white noise.

Time	Effect size change in trend																													
	0.01	0.02	0.03	0.04	0.05	0.06	0.07	0.08	0.09	0.1	0.11	0.12	0.13	0.14	0.15	0.16	0.17	0.18	0.19	0.2	0.21	0.22	0.23	0.24	0.25	0.26	0.27	0.28	0.29	0.30
40	0.14	0.15	0.16	0.17	0.20	0.22	0.24	0.27	0.30	0.33	0.36	0.40	0.44	0.47	0.50	0.54	0.59	0.62	0.65	0.68	0.70	0.74	0.77	0.79	0.81	0.84	0.86	0.89	0.91	0.93
60	0.13	0.13	0.14	0.18	0.22	0.27	0.32	0.39	0.45	0.52	0.59	0.65	0.71	0.76	0.82	0.86	0.89	0.91	0.94	0.96	0.97	0.98	0.99	1.00	1.00	1.00	1.00	1.00	1.00	1.00
80	0.10	0.13	0.20	0.26	0.35	0.45	0.56	0.66	0.75	0.83	0.88	0.93	0.96	0.98	0.99	0.99	0.99	1.00	1.00	1.00	1.00	1.00	1.00	1.00	1.00	1.00	1.00	1.00	1.00	1.00
100	0.10	0.17	0.28	0.40	0.55	0.68	0.79	0.88	0.93	0.97	0.99	1.00	1.00	1.00	1.00	1.00	1.00	1.00	1.00	1.00	1.00	1.00	1.00	1.00	1.00	1.00	1.00	1.00	1.00	1.00
120	0.12	0.21	0.37	0.54	0.73	0.85	0.94	0.98	1.00	1.00	1.00	1.00	1.00	1.00	1.00	1.00	1.00	1.00	1.00	1.00	1.00	1.00	1.00	1.00	1.00	1.00	1.00	1.00	1.00	1.00
140	0.11	0.26	0.51	0.72	0.88	0.96	1.00	1.00	1.00	1.00	1.00	1.00	1.00	1.00	1.00	1.00	1.00	1.00	1.00	1.00	1.00	1.00	1.00	1.00	1.00	1.00	1.00	1.00	1.00	1.00
160	0.16	0.38	0.65	0.87	0.98	1.00	1.00	1.00	1.00	1.00	1.00	1.00	1.00	1.00	1.00	1.00	1.00	1.00	1.00	1.00	1.00	1.00	1.00	1.00	1.00	1.00	1.00	1.00	1.00	1.00
180	0.19	0.49	0.80	0.96	1.00	1.00	1.00	1.00	1.00	1.00	1.00	1.00	1.00	1.00	1.00	1.00	1.00	1.00	1.00	1.00	1.00	1.00	1.00	1.00	1.00	1.00	1.00	1.00	1.00	1.00
200	0.21	0.58	0.90	0.99	1.00	1.00	1.00	1.00	1.00	1.00	1.00	1.00	1.00	1.00	1.00	1.00	1.00	1.00	1.00	1.00	1.00	1.00	1.00	1.00	1.00	1.00	1.00	1.00	1.00	1.00
220	0.25	0.69	0.95	1.00	1.00	1.00	1.00	1.00	1.00	1.00	1.00	1.00	1.00	1.00	1.00	1.00	1.00	1.00	1.00	1.00	1.00	1.00	1.00	1.00	1.00	1.00	1.00	1.00	1.00	1.00
240	0.29	0.78	0.99	1.00	1.00	1.00	1.00	1.00	1.00	1.00	1.00	1.00	1.00	1.00	1.00	1.00	1.00	1.00	1.00	1.00	1.00	1.00	1.00	1.00	1.00	1.00	1.00	1.00	1.00	1.00
260	0.38	0.88	1.00	1.00	1.00	1.00	1.00	1.00	1.00	1.00	1.00	1.00	1.00	1.00	1.00	1.00	1.00	1.00	1.00	1.00	1.00	1.00	1.00	1.00	1.00	1.00	1.00	1.00	1.00	1.00
280	0.43	0.93	1.00	1.00	1.00	1.00	1.00	1.00	1.00	1.00	1.00	1.00	1.00	1.00	1.00	1.00	1.00	1.00	1.00	1.00	1.00	1.00	1.00	1.00	1.00	1.00	1.00	1.00	1.00	1.00
300	0.48	0.96	1.00	1.00	1.00	1.00	1.00	1.00	1.00	1.00	1.00	1.00	1.00	1.00	1.00	1.00	1.00	1.00	1.00	1.00	1.00	1.00	1.00	1.00	1.00	1.00	1.00	1.00	1.00	1.00
320	0.56	0.99	1.00	1.00	1.00	1.00	1.00	1.00	1.00	1.00	1.00	1.00	1.00	1.00	1.00	1.00	1.00	1.00	1.00	1.00	1.00	1.00	1.00	1.00	1.00	1.00	1.00	1.00	1.00	1.00
340	0.64	1.00	1.00	1.00	1.00	1.00	1.00	1.00	1.00	1.00	1.00	1.00	1.00	1.00	1.00	1.00	1.00	1.00	1.00	1.00	1.00	1.00	1.00	1.00	1.00	1.00	1.00	1.00	1.00	1.00
360	0.73	1.00	1.00	1.00	1.00	1.00	1.00	1.00	1.00	1.00	1.00	1.00	1.00	1.00	1.00	1.00	1.00	1.00	1.00	1.00	1.00	1.00	1.00	1.00	1.00	1.00	1.00	1.00	1.00	1.00
380	0.79	1.00	1.00	1.00	1.00	1.00	1.00	1.00	1.00	1.00	1.00	1.00	1.00	1.00	1.00	1.00	1.00	1.00	1.00	1.00	1.00	1.00	1.00	1.00	1.00	1.00	1.00	1.00	1.00	1.00
400	0.83	1.00	1.00	1.00	1.00	1.00	1.00	1.00	1.00	1.00	1.00	1.00	1.00	1.00	1.00	1.00	1.00	1.00	1.00	1.00	1.00	1.00	1.00	1.00	1.00	1.00	1.00	1.00	1.00	1.00

*Note*: In the simulations it was assumed that there was no underlying trend or covariates that needed to be adjusted for. The intervention was assumed to have occurred at the midpoint of the time series.

The relationship between effect size and power demonstrates a sigmoidal trend for a step change and change in trend. Initially, modest increases in effect size yield substantial gains in power, showing a steep, accelerating rise. Series length also demonstrates an increasing relationship with power, with decelerating returns as power approaches its maximum. In contrast, for a pulse effect, there is a sharp cut‐off between a lack of power (<80%) and power (>80%) at an effect size of 2.5, and no obvious gains in increasing the series length.

The supporting information provides further lookup tables for varying degrees of AR(1) autocorrelation (0.1, 0.5 and 0.9) and standard deviation of white noise (0.5, 1.0, 2.0, 3.0, 4.0 and 5.0), for step‐level changes (Appendix S2), pulse effects (Appendix S3) and change in trend (Appendix S4). These show that as the standard deviation of white noise increases, the power to detect an effect consistently drops across all levels of autocorrelation. This reduction is particularly pronounced for shorter series lengths when detecting a step effect, as the signal becomes increasingly lost in the heightened background variability.

Increases in autocorrelation do not appear to have similar effects across intervention effect types. The power to detect an effect consistently drops across varying levels of standard deviations in white noise with increasing autocorrelation for step effects and changes in trend, but increases for pulse effects.

## DISCUSSION

The main contribution of this article lies in providing accessible and practical tools for power and sample size determination in ITS designs. Our primary output includes systematically generated lookup tables, comprehensive R code for power simulations and the intuitive Shiny app. These resources are valuable for prospective study planning, enabling researchers to proactively estimate statistical power and determine the necessary sample size requirements before data collection begins, to aid the selection of suitable data sets or the timepoint at which it is sensible to conduct an analysis. This ensures that studies are adequately powered to detect meaningful intervention effects, thereby guiding more informed and robust study designs.

Beyond prospective planning, these tools are also beneficial for the retrospective analysis of secondary data. Rather than merely performing traditional post‐hoc power calculations, which can be misleading [36], researchers can utilise the R code or Shiny app with the observed characteristics of their existing data (such as actual series length, estimated autocorrelation and noise levels) to precisely determine the minimum effect size that was detectable at a given power level. This provides a crucial understanding of the sensitivity of their existing time series to detect a step change or pulse effect of a certain magnitude. This approach also helps inform the design and data requirements for future studies, and can assist in determining the appropriateness of an ARIMA model for their collected data, considering its inherent characteristics and the expected effect size.

### Comparison with previous studies

Many researchers rely on rules of thumb for ITS, such as requiring a minimum of 50–100 observation periods, more observation periods than model parameters, and equal proportions of data collection before and after an intervention [8, 9, 10, 11]. The findings here show that this may not be an adequate series length if variability in the data is high. In line with previous studies, we found that increasing the standard deviation of white noise significantly lowers the power to detect changes [25]. To mitigate this, researchers can consider aggregating data (e.g. from monthly to quarterly), which can help average out random fluctuations and produce a smoother time series. This is common practice in time series analysis to reduce noise and reveal underlying patterns but may not be suitable for pulse effects occurring over a limited period.

The observed decelerating returns in power as series length increases for a step level change and change in trend occurs due to the inherent statistical properties of time series, where initial increases in data dramatically reduce sampling variability, but these marginal gains diminish as the sample size becomes very large, and the estimate approaches its true value. Essentially, the signal of the intervention effect becomes sufficiently clear with a reasonable volume of data, and further increases in series length primarily contribute to refining an already robust estimate, rather than fundamentally changing its detectability [11, 15].

Furthermore, consistent with previous findings, lower autocorrelation appeared to facilitate better detection power across various sample sizes and effect sizes for step changes and a change in trend [11, 21, 22]. This occurs because the noise introduced by the strong relationship between consecutive data points drowns out the signal of sustained changes, making it difficult to distinguish them from the natural fluctuations of the autocorrelated series. In contrast, autocorrelation may enhance the power for detecting pulse effects, as short‐term deviations become more distinguishable against a smoother, more predictable background [14]. Therefore, reducing autocorrelation when assessing step changes and changes in trend could enhance the sensitivity of the analysis. Practical strategies include improving data quality through increased sampling frequency and incorporating additional control variables that might explain sequential dependence.

### Recommendations and limitations

While the described R code and Shiny app provide a robust framework for estimating statistical power in ITS designs it is important to acknowledge several limitations inherent in its current implementation.

First and foremost, the simulator exclusively models abrupt, step‐level changes and abrupt pulse (temporary) effects of a specified duration. It can also model an additional change in trend, where the rate of change in an outcome is altered after the intervention. This deliberate choice means it currently excludes more complex dynamic responses often seen in real‐world interventions, including gradual onsets where the effect builds over time. Simulating such effects would require incorporating transfer functions within the ARIMA framework, which are mathematical models used to describe how an input series affects an output series over time, representing delayed or cumulative effects, a feature not currently integrated. Consequently, researchers evaluating interventions expected to show these complex patterns should interpret the power estimates for step, trend changes and pulse changes cautiously or explore alternative power estimation methods.

The simulator also imposes specific constraints on error structures and variable modelling. The error structure is confined to non‐seasonal ARIMA models, meaning it cannot simulate or account for seasonality, a common pattern in many public health and policy time series. Additionally, the current version only supports a single continuous covariate, which is generated via a fixed AR(1) process. This restricts its ability to explore scenarios with multiple covariates, categorical predictors or alternative, more intricate covariate data‐generating processes. Similarly, the trend modelling options are limited to linear deterministic or stochastic (random walk) trends. It does not support non‐linear trends (like quadratic or exponential patterns) or the complex interplay of multiple trend types that might exist in real‐world data.

Furthermore, the current implementation does not account for potential non‐stationarity beyond simple differencing, nor does it explicitly model structural breaks in the time series that are unrelated to the intervention. It also assumes a fixed sampling interval and does not readily adapt to irregularly spaced data. Finally, the simulator operates under several key assumptions about the data it processes. It assumes complete data with Gaussian‐distributed errors, meaning it does not directly accommodate common real‐world complexities such as missing observations, the presence of outliers or non‐normal error distributions. Therefore, when deriving parameters for use in this power simulation app from data, such as the standard deviation of the noise and autocorrelation coefficients, it is crucial that it has been appropriately handled for missingness and extreme values. Additionally, applying transformations (e.g. a log transformation) to your data before estimating these parameters for the simulator can help stabilise the variance and make the error distribution more amenable to the underlying Gaussian assumptions of the simulator.

Nonetheless, given the inherent complexity of simulating highly varied ITS scenarios, this app and code offer a valuable and computationally efficient tool for power estimation under common and well‐defined ITS model specifications. It provides a strong foundation for researchers to understand power in many practical contexts, guiding more informed study design and interpretation.

## CONCLUSION

This study offers valuable guidance for researchers planning ITS analyses, particularly in the field of addiction. By providing lookup tables, R code and the Shiny app, along with clear recommendations, it hopes to ensure that studies are adequately powered to detect meaningful intervention effects.

## AUTHOR CONTRIBUTIONS

**Emma Beard:** Conceptualization (lead); formal analysis (lead); methodology (equal); writing—original draft (lead); writing—review and editing (equal). **Jamie Brown:** Methodology (equal); writing—review and editing (equal). **Lion Shahab:** Methodology (equal); writing—review and editing (equal).

## DECLARATION OF INTERESTS

None.

## Supporting information


**Appendix S1.** Detailed simulation process.

**Appendix S2.** Lookup tables for step‐level change.

**Appendix S3.** Lookup tables for pulse change.

**Appendix S4.** Lookup tables for change in trend.

**Appendix S5.** Examples of Shiny app configurations.

## Data Availability

The R code for the power simulations is given on GitHub https://github.com/EmmaBeardUCL/ARIMA_ITS_PowerSimulations/blob/main/its_power_simulator.
