# Supplementary material for: Sample size requirements to evaluate policies in addiction research using interrupted time series analysis (ITS): Tools and guidance
Source: Addiction. 2025 Nov 11;121(3):695–712. doi: 10.1111/add.70220 (PMC12887929; doi:10.1111/add.70220)
Supplement: Supplementary file 1 — Appendix S1. Detailed simulation process. [file ADD-121-695-s003.docx]

**Supplementary materials: detailed simulation process**

This supplementary document provides a detailed explanation of the its_power_simulator function an R-based tool designed to estimate statistical power for Interrupted Time Series (ITS) designs through Monte Carlo simulations. The final function is defined as follows:

its_power_simulator(t, errormodel = list(order = c(0, 0, 0), ar = NULL, ma = NULL), period = 1, k, b0 = 0, b1_effect = 0, intervention_type = "step", pulse_duration = 1, b2_covariate = 0, b2_mean = 0, b2_sd = 1, b2_shift_after_k = 0, noise_sd = 0, b3_trend = 0, include_covariate = FALSE, model_covariate = FALSE, include_trend = FALSE, trend_model_method = "none", trend_type = "deterministic", stochastic_trend_sd = 1, log_transform_outcome = FALSE, seed = NULL, n.sims)

**Defining baseline parameters and data generating process**

The its_power_simulator first constructs a synthetic dataset based on user-defined parameters.

I. Core time series parameters:

- *Time period of data collection (t):* An integer specifying the total number of time points in the series (e.g., t=100 for 100 months of data collection).
- *Intervention time point (k):* An integer specifying the number of pre-intervention time points. The intervention (step change) is modelled as occurring starting at time point $k+1$. This value must be an integer $< t-1$ (inclusive).
- *Period of data collection (period):* An integer indicating the frequency of observations per unit time (e.g., period = 12 for monthly data, period = 4 for quarterly data). This parameter is primarily used when creating R's time series (ts) objects for appropriate time series handling.

II. Main parameters for coefficients (deterministic components):

These parameters define the fixed, systematic parts of the data generating process.

- *Intercept (b0​):* The starting value or average level of your dependent variable before any intervention or trend effects. This sets the overall magnitude of your series.
- *Intervention type (intervention_type):* If ‘step’ the intervention is modelled as a binary variable (0 before the intervention, 1 after). If 'pulse' the intervention is modelled as a binary variable that is 1 for a specified pulse_duration starting at $k+1$, and 0 otherwise. If ‘trend_change’, the intervention is modelled as zero before the intervention ($k$) and increases linearly thereafter.
- *Intervention effect size (b1_effect):* The numeric value reflecting the true effect size for the immediate impact of the intervention. This parameter's interpretation depends on the intervention_type. For a step change represents the immediate and sustained change in the outcome after the intervention. For a pulse effect this represents the magnitude of the temporary change in the outcome during the pulse duration. For a change in trend, this represents the change in the slope of the outcome variable after the intervention. A positive value means the slope increases, a negative value means it decreases.
- *Pulse duration (pulse_duration):* If a pulse effect is specified this indicates how many time periods ($t$) the effect occurs for.
- Trend magnitude factor (b3_trend): This parameter defines the magnitude or scale of the trend component:
  - For deterministic trends (if include_trend = TRUE and trend_type = "deterministic"): This acts as the slope of the linear trend. It indicates a consistent average change in the outcome per unit of time. A value of 0 means no deterministic trend.
  - For stochastic trends (if include_trend = TRUE and trend_type = "stochastic"): This acts as a scaling factor applied to the cumulative sum of the random innovations (which includes variability controlled by stochastic_trend_sd). It influences the overall magnitude and volatility of the stochastic trend's impact on the outcome. While a value of 1 means the raw stochastic trend is added directly (and should be used as the default), a value of 0 effectively removes its contribution; other positive values can amplify (e.g., 2.0) or dampen (e.g., 0.5) its effect.

III. Continuous covariate parameters (if include_covariate = TRUE):

The function allows for the inclusion of a simulated continuous covariate ($X_{2}$​) in the data generating process, offering flexibility to model potential confounders or predictors.

- *Covariate effect size (b2_covariate​):* The numeric value representing the true effect size (beta coefficient) for the continuous numeric covariate. Set this to 0 if no numeric covariate is being modelled in your simulation.
- *Covariate mean (b2_mean​):* The mean value for the simulated continuous covariate, which is generated from a normal distribution.
- *Covariate standard deviation (b2_sd​):* The standard deviation for the simulated continuous covariate.
- *Covariate shift after intervention (b2_shift_after_k​):* Represents a deterministic change in the mean of the covariate after the intervention time point (k). This allows for the modelling of a time-varying confounder whose trajectory shifts at the intervention. This shift is added to the covariate's baseline and stochastic component from time $k+1$ onwards.

IV. Main parameters for the error component and trend component (stochastic components):

These parameters define the random, unpredictable parts of the data generating process.

- *Standard deviation of white noise (noise_sd):* This is the standard deviation of the innovations (white noise) that drive the ARIMA error process. It represents the variability or dispersion of the random errors around their mean (usually zero). A greater noise_sd makes it harder to detect an underlying association.
- *Autocorrelation model (errormodel):* This list defines the ARIMA error structure for the simulated data. It must contain order = c(p, d, q), where:
  - p is the number of autoregressive (AR) terms. AR terms represent the dependency of the current value on its previous values (e.g., an AR(1) model means the current value depends on the immediately preceding value).
  - d is the differencing order (typically 0 for stationary series).
  - q is the number of moving average (MA) terms. MA terms represent the dependency on past error terms (e.g., an MA(1) model implies the current value depends on the most recent error term).

It optionally contains ar (a numeric vector of AR coefficients) and ma (a numeric vector of MA coefficients). These values indicate the strength of the dependency. For example, an ar coefficient of 0.8 for an AR(1) suggests that the current value is influenced by 80% of the previous value. If more than one AR or MA term is used (e.g., AR(2)), then the (c) command (which combines values into a vector) should be used for the coefficients (e.g., ar = c(0.5, 0.2)). If ar or ma are not provided, arima.sim will generate coefficients internally based on the order.

- *Stochastic Trend Standard Deviation (stochastic_trend_sd) (if include_trend = TRUE and trend_type = "stochastic"):* This determines the standard deviation of the white noise innovations (random shocks) that form the basis of the stochastic trend (random walk). A larger value means greater volatility and more pronounced random fluctuations in the underlying increments of the stochastic trend.

V. Simulation control and output options:

- *Log transform outcome (log_transform_outcome):* A logical value (TRUE or FALSE). If TRUE, the outcome variable will be log-transformed before fitting the ARIMA model. If FALSE, the original scale of the outcome variable will be used.
- *Include covariate (include_covariate):* A logical value (TRUE or FALSE). If TRUE, a stochastic, time-varying covariate (X2​) is simulated and included in the true data-generating process.
- *Model covariate (model_covariate):* A logical value (TRUE or FALSE). If TRUE, the simulated covariate is included as an exogenous regressor in the fitted ARIMA model.
- *Include trend (include_trend):* A logical value (TRUE or FALSE). If TRUE, a trend will be included depending on the trend type.
- *Trend Type (trend_type):* A character string specifying the type of trend in the DGP. Options are:
  - ‘deterministic’: A trend that can be expressed as a predictable, non-random function of time (e.g., a straight line). This is trend-stationary.
  - ‘stochastic’: A trend that is not predictable and changes randomly over time (represented by a random walk). This is difference-stationary.
- *Trend model method (trend_model_method):* A character string specifying how the time trend is modelled in the regression. Options are:
  - ‘none’: The time trend is not modelled in the regression.
  - ‘xreg’: The time trend is included as an exogenous regressor in the arima() model.
  - ‘differencing’: The ARIMA model's differencing order (d) is set to 1, effectively modelling a linear trend (if a constant mean is assumed in the differenced series).
- *Random seed (seed):* An optional integer argument that allows you to set the initial state of the random number generator. Using a specific seed ensures that the sequence of random numbers generated will be the same each time the function is run, making your simulations reproducible. For the look-up tables provided, this was set to 123.
- *Number of simulations (n.sims):* The number of Monte Carlo simulations to run. A higher number of simulations provides a more stable estimate of power.

Data Generating Mechanism Details

The its_power_simulator function constructs the outcome variable based on the specified parameters. The underlying data generating process (DGP) for the outcome is a linear predictor combined with autocorrelated errors.

$$Y_{t}=b_{0}+b_{1}Intervention effect\times X_{1t}+b_{2}Covariate\times X_{2t}+b_{3}trend\times{Time}_{t}+{Error}_{t}$$

Where:

- $Y_{t}$​ is the outcome at time t.
- $b_{0}$​ is the intercept.
- $X_{1t}$​ is the intervention indicator variable (representing a pulse, step or change in trend)
- $b_{1}Intervention effect$ ​represents the true effect size of the intervention. For a step change, this is the immediate and sustained change. For a pulse change, this is the temporary change lasting for pulse_duration time points. For a change in trend, this represents the magnitude of the alteration to the trend occurring at the intervention point.
- $X_{2t}$is the simulated continuous covariate. This covariate is constructed with both stochastic and deterministic components:
  - *Stochastic component:* It is built from an AR(1) process with an AR coefficient of 0.5, capturing its natural autocorrelation and random fluctuations
  - *Deterministic component:* An optional linear shift, b2_shift_after_k​, is added to $X_{2t}$​ for $t>k$. This allows $X_{2t}$​ to act as a time-varying confounder whose own trajectory changes with the intervention.
- $b_{2}Covariate$ ​represents the true effect size of the covariate.
- ${Time}_{t}$is the linear time trend.
  - If trend_type = "deterministic", ${Time}_{t}$ is coded as $1,2,\ldots,t$. $b_{3}$ (b3_trend) is the effect size of this trend on the outcome.
  - If trend_type = "stochastic", ${Time}_{t}$ is generated as a cumulative sum of random innovations with a mean defined by b3_stochastic_drift and a standard deviation of stochastic_trend_sd (i.e., a random walk, potentially with drift).
- $b_{3}Trend$ ​ is the effect size of the deterministic trend on the outcome. For a stochastic trend, it scales ${Time}_{t}$ to controls the overall magnitude of the stochastic trend's influence on the outcome.
- ${Error}_{t}$represents the random error component, generated using the arima.sim function to ensure proper autocorrelation structures based on the errormodel (order, AR, MA coefficients) and noise_sd. A burn-in period (set to 80% of t in the innovation generation) is used. This initial set of observations is discarded to allow the model to stabilize and reduce the influence of arbitrary starting conditions, thus producing more reliable results.

**Simulation process**

The its_power_simulator function performs a Monte Carlo simulation by repeating the following steps n.sims times:

I. Data Generation:

- A dependent variable is constructed based on the specified baseline parameters (b0​, b1_effect​, b2_covariate​, b3_trend​) and simulated independent variables ($X_{1}$, $X_{2}$​, Time).
- The intervention effect ($X_{1}$) is modelled according to the specified intervention_type. For a step change, it is a binary variable (0 before time $k+1$, 1 from time $k+1$ onwards). For a pulse' change, it is a binary variable that is 1 for pulse_duration time points starting from $k+1$, and 0 otherwise. For a **change in** trend, the intervention effect is 0 before the intervention time (k). After time which, it changes proportionally to the time elapsed since the intervention.
- A continuous covariate ($X_{2}$​) is built from a stochastic AR(1) process that captures its natural autocorrelation and random fluctuations. There is also an optional deterministic linear trend change that begins at the intervention point, allowing it to act as a time-varying confounder whose own trajectory changes with the intervention.
- A time trend variable is generated according to the specified trend_type (deterministic linear trend or stochastic random walk).
- Random error simulation: Autocorrelated errors are generated using the arima.sim function, which is computationally efficient and ensures proper autocorrelation structures. The burn-in period for generating autocorrelated errors is calculated as $5 * max(p, q)$ (where p and q are the AR and MA orders, respectively).This initial set of observations is discarded to allow the model to stabilise and reduce the influence of arbitrary starting conditions, thus producing more reliable results.
- The generated variables (Y, $X_{1}$​, $X_{2}$​, Time) are combined into a data frame suitable for the ARIMA model.
- If log_transform_outcome is TRUE, the simulated Y is then log-transformed for the subsequent ARIMA model fitting.

II. ARIMA Model Analysis:

- The simulated dataset is analysed using an ARIMAX model via the arima() function. The exogenous regressors always include the intervention variable ($X_{1}$​) and conditionally include the covariate ($X_{2}$) and the time trend (depending on model_covariate and trend_model_method). This approach ensures that the model estimates their effects regardless of whether their true effect sizes were set to zero in the data-generating process. This mirrors common analytical practices where researchers include control variables to enhance precision and account for underlying patterns, even if some adjustments might ultimately be for non-confounding factors in real-world scenarios.
- The optim.control = list(maxit = 1000) parameter is used in the arima() function to increase the maximum number of iterations for the optimization algorithm, aiming to improve convergence and the reliability of coefficient estimates.
- The coefficient for the intervention effect (b1_effect​) is extracted, and its statistical significance (p-value < 0.05) is recorded.

While the TSA package in R offers advanced capabilities for modelling dynamic relationships between variables through explicit transfer functions (e.g., using arimax()), our simulation utilises the more direct arima() function with xreg. This choice is grounded in its common application for many standard ITS designs, particularly those primarily focused on assessing a simple, immediate, and sustained step change or a temporary pulse change in the outcome.

III. Power calculation:

After all simulations are completed, the proportion of times the intervention coefficient was found to be statistically significant (p-value < 0.05) is calculated. This proportion represents the estimated power of the ITS design under the specified parameters.

**Function output**

The its_power_simulator function returns and prints a list containing the following key results:

- $power: The proportion of simulations where the intervention effect was statistically significant (p < 0.05). This is the primary measure of the statistical power.
- $pvalue: The average p-value for the intervention coefficient across all simulations.
- $beta_iv: The average estimated coefficient value for the intervention effect across all simulations.
- $beta_covariate: The average estimated coefficient value for the covariate across all simulations.
- $beta_time: The average estimated coefficient value for the time trend across all simulations. Note that if the trend was modelled using differencing, this will be NA as differencing accounts for the trend implicitly, rather than estimating a coefficient for it.
- $mean_simulated_stochastic_trend_magnitude: The average value of the simulated stochastic trend component at the last time point (t) across all simulations. This provides an idea of the typical endpoint value of the random walk process.
- $last_sim_data: The data frame from the last simulation run, useful for inspecting the generated data and fitting visually.
- $errors: A vector containing any error messages encountered during the simulation process.
- $warnings: A vector containing any warning messages encountered during the simulation process.

**Internal Safeguards**

There is an internal safeguard within its_power_simulator, ensures that the specified ARIMA model orders (p,d,q) are non-negative. This helps to prevent issues like excessively large models that might lead to computational difficulties or non-interpretable results. While this check does not rigorously verify the mathematical conditions for stationarity or invertibility of the coefficients, the arima.sim function itself handles these properties when generating the errors. Additionally, b2_sd (Covariate SD) and stochastic_trend_sd (Stochastic Trend SD) are checked to ensure they are non-negative when their respective components are included in the DGP. For 'pulse' interventions, pulse_duration is validated to be a positive integer and not to exceed the number of post-intervention time points ($t - k$)
