# Supplementary material for: Sample size requirements to evaluate policies in addiction research using interrupted time series analysis (ITS): Tools and guidance
Source: Addiction. 2025 Nov 11;121(3):695–712. doi: 10.1111/add.70220 (PMC12887929; doi:10.1111/add.70220)
Supplement: Supplementary file 4 — Appendix S4. Lookup tables for change in trend. [file ADD-121-695-s005.docx]

Table 1: Power to detect a change in trend as a function of time and size of the change in trend, with the presence of AR= 0.1 autocorrelation and standard deviation of the white noise of 0.5.

|  | Effect size for the change in trend | | | | | | | | | | | | | | | | | | | | | | | | | | | | | |
| --- | --- | --- | --- | --- | --- | --- | --- | --- | --- | --- | --- | --- | --- | --- | --- | --- | --- | --- | --- | --- | --- | --- | --- | --- | --- | --- | --- | --- | --- | --- |
| Time | 0.01 | 0.02 | 0.03 | 0.04 | 0.05 | 0.06 | 0.07 | 0.08 | 0.09 | 0.1 | 0.11 | 0.12 | 0.13 | 0.14 | 0.15 | 0.16 | 0.17 | 0.18 | 0.19 | 0.2 | 0.21 | 0.22 | 0.23 | 0.24 | 0.25 | 0.26 | 0.27 | 0.28 | 0.29 | 0.3 |
| 40 | 0.11 | 0.16 | 0.25 | 0.33 | 0.45 | 0.57 | 0.70 | 0.82 | 0.88 | 0.94 | 0.97 | 0.99 | 0.99 | 1.00 | 1.00 | 1.00 | 1.00 | 1.00 | 1.00 | 1.00 | 1.00 | 1.00 | 1.00 | 1.00 | 1.00 | 1.00 | 1.00 | 1.00 | 1.00 | 1.00 |
| 60 | 0.13 | 0.28 | 0.51 | 0.74 | 0.90 | 0.96 | 0.99 | 1.00 | 1.00 | 1.00 | 1.00 | 1.00 | 1.00 | 1.00 | 1.00 | 1.00 | 1.00 | 1.00 | 1.00 | 1.00 | 1.00 | 1.00 | 1.00 | 1.00 | 1.00 | 1.00 | 1.00 | 1.00 | 1.00 | 1.00 |
| 80 | 0.20 | 0.53 | 0.83 | 0.97 | 1.00 | 1.00 | 1.00 | 1.00 | 1.00 | 1.00 | 1.00 | 1.00 | 1.00 | 1.00 | 1.00 | 1.00 | 1.00 | 1.00 | 1.00 | 1.00 | 1.00 | 1.00 | 1.00 | 1.00 | 1.00 | 1.00 | 1.00 | 1.00 | 1.00 | 1.00 |
| 100 | 0.31 | 0.76 | 0.98 | 1.00 | 1.00 | 1.00 | 1.00 | 1.00 | 1.00 | 1.00 | 1.00 | 1.00 | 1.00 | 1.00 | 1.00 | 1.00 | 1.00 | 1.00 | 1.00 | 1.00 | 1.00 | 1.00 | 1.00 | 1.00 | 1.00 | 1.00 | 1.00 | 1.00 | 1.00 | 1.00 |
| 120 | 0.43 | 0.94 | 1.00 | 1.00 | 1.00 | 1.00 | 1.00 | 1.00 | 1.00 | 1.00 | 1.00 | 1.00 | 1.00 | 1.00 | 1.00 | 1.00 | 1.00 | 1.00 | 1.00 | 1.00 | 1.00 | 1.00 | 1.00 | 1.00 | 1.00 | 1.00 | 1.00 | 1.00 | 1.00 | 1.00 |
| 140 | 0.63 | 0.99 | 1.00 | 1.00 | 1.00 | 1.00 | 1.00 | 1.00 | 1.00 | 1.00 | 1.00 | 1.00 | 1.00 | 1.00 | 1.00 | 1.00 | 1.00 | 1.00 | 1.00 | 1.00 | 1.00 | 1.00 | 1.00 | 1.00 | 1.00 | 1.00 | 1.00 | 1.00 | 1.00 | 1.00 |
| 160 | 0.76 | 1.00 | 1.00 | 1.00 | 1.00 | 1.00 | 1.00 | 1.00 | 1.00 | 1.00 | 1.00 | 1.00 | 1.00 | 1.00 | 1.00 | 1.00 | 1.00 | 1.00 | 1.00 | 1.00 | 1.00 | 1.00 | 1.00 | 1.00 | 1.00 | 1.00 | 1.00 | 1.00 | 1.00 | 1.00 |
| 180 | 0.89 | 1.00 | 1.00 | 1.00 | 1.00 | 1.00 | 1.00 | 1.00 | 1.00 | 1.00 | 1.00 | 1.00 | 1.00 | 1.00 | 1.00 | 1.00 | 1.00 | 1.00 | 1.00 | 1.00 | 1.00 | 1.00 | 1.00 | 1.00 | 1.00 | 1.00 | 1.00 | 1.00 | 1.00 | 1.00 |
| 200 | 0.95 | 1.00 | 1.00 | 1.00 | 1.00 | 1.00 | 1.00 | 1.00 | 1.00 | 1.00 | 1.00 | 1.00 | 1.00 | 1.00 | 1.00 | 1.00 | 1.00 | 1.00 | 1.00 | 1.00 | 1.00 | 1.00 | 1.00 | 1.00 | 1.00 | 1.00 | 1.00 | 1.00 | 1.00 | 1.00 |
| 220 | 0.99 | 1.00 | 1.00 | 1.00 | 1.00 | 1.00 | 1.00 | 1.00 | 1.00 | 1.00 | 1.00 | 1.00 | 1.00 | 1.00 | 1.00 | 1.00 | 1.00 | 1.00 | 1.00 | 1.00 | 1.00 | 1.00 | 1.00 | 1.00 | 1.00 | 1.00 | 1.00 | 1.00 | 1.00 | 1.00 |
| 240 | 1.00 | 1.00 | 1.00 | 1.00 | 1.00 | 1.00 | 1.00 | 1.00 | 1.00 | 1.00 | 1.00 | 1.00 | 1.00 | 1.00 | 1.00 | 1.00 | 1.00 | 1.00 | 1.00 | 1.00 | 1.00 | 1.00 | 1.00 | 1.00 | 1.00 | 1.00 | 1.00 | 1.00 | 1.00 | 1.00 |
| 260 | 1.00 | 1.00 | 1.00 | 1.00 | 1.00 | 1.00 | 1.00 | 1.00 | 1.00 | 1.00 | 1.00 | 1.00 | 1.00 | 1.00 | 1.00 | 1.00 | 1.00 | 1.00 | 1.00 | 1.00 | 1.00 | 1.00 | 1.00 | 1.00 | 1.00 | 1.00 | 1.00 | 1.00 | 1.00 | 1.00 |
| 280 | 1.00 | 1.00 | 1.00 | 1.00 | 1.00 | 1.00 | 1.00 | 1.00 | 1.00 | 1.00 | 1.00 | 1.00 | 1.00 | 1.00 | 1.00 | 1.00 | 1.00 | 1.00 | 1.00 | 1.00 | 1.00 | 1.00 | 1.00 | 1.00 | 1.00 | 1.00 | 1.00 | 1.00 | 1.00 | 1.00 |
| 300 | 1.00 | 1.00 | 1.00 | 1.00 | 1.00 | 1.00 | 1.00 | 1.00 | 1.00 | 1.00 | 1.00 | 1.00 | 1.00 | 1.00 | 1.00 | 1.00 | 1.00 | 1.00 | 1.00 | 1.00 | 1.00 | 1.00 | 1.00 | 1.00 | 1.00 | 1.00 | 1.00 | 1.00 | 1.00 | 1.00 |
| 320 | 1.00 | 1.00 | 1.00 | 1.00 | 1.00 | 1.00 | 1.00 | 1.00 | 1.00 | 1.00 | 1.00 | 1.00 | 1.00 | 1.00 | 1.00 | 1.00 | 1.00 | 1.00 | 1.00 | 1.00 | 1.00 | 1.00 | 1.00 | 1.00 | 1.00 | 1.00 | 1.00 | 1.00 | 1.00 | 1.00 |
| 340 | 1.00 | 1.00 | 1.00 | 1.00 | 1.00 | 1.00 | 1.00 | 1.00 | 1.00 | 1.00 | 1.00 | 1.00 | 1.00 | 1.00 | 1.00 | 1.00 | 1.00 | 1.00 | 1.00 | 1.00 | 1.00 | 1.00 | 1.00 | 1.00 | 1.00 | 1.00 | 1.00 | 1.00 | 1.00 | 1.00 |
| 360 | 1.00 | 1.00 | 1.00 | 1.00 | 1.00 | 1.00 | 1.00 | 1.00 | 1.00 | 1.00 | 1.00 | 1.00 | 1.00 | 1.00 | 1.00 | 1.00 | 1.00 | 1.00 | 1.00 | 1.00 | 1.00 | 1.00 | 1.00 | 1.00 | 1.00 | 1.00 | 1.00 | 1.00 | 1.00 | 1.00 |
| 380 | 1.00 | 1.00 | 1.00 | 1.00 | 1.00 | 1.00 | 1.00 | 1.00 | 1.00 | 1.00 | 1.00 | 1.00 | 1.00 | 1.00 | 1.00 | 1.00 | 1.00 | 1.00 | 1.00 | 1.00 | 1.00 | 1.00 | 1.00 | 1.00 | 1.00 | 1.00 | 1.00 | 1.00 | 1.00 | 1.00 |
| 400 | 1.00 | 1.00 | 1.00 | 1.00 | 1.00 | 1.00 | 1.00 | 1.00 | 1.00 | 1.00 | 1.00 | 1.00 | 1.00 | 1.00 | 1.00 | 1.00 | 1.00 | 1.00 | 1.00 | 1.00 | 1.00 | 1.00 | 1.00 | 1.00 | 1.00 | 1.00 | 1.00 | 1.00 | 1.00 | 1.00 |

Note: In the simulations it was assumed that there was no underlying trend or covariates which needed to be adjusted for. The intervention was assumed to have occurred at the midpoint of the time series.

Table 2: Power to detect a change in trend as a function of time and size of the change in trend, with the presence of AR= 0.5 autocorrelation and standard deviation of the white noise of 0.5.

|  | Effect size for the change in trend | | | | | | | | | | | | | | | | | | | | | | | | | | | | | |
| --- | --- | --- | --- | --- | --- | --- | --- | --- | --- | --- | --- | --- | --- | --- | --- | --- | --- | --- | --- | --- | --- | --- | --- | --- | --- | --- | --- | --- | --- | --- |
| Time | 0.01 | 0.02 | 0.03 | 0.04 | 0.05 | 0.06 | 0.07 | 0.08 | 0.09 | 0.1 | 0.11 | 0.12 | 0.13 | 0.14 | 0.15 | 0.16 | 0.17 | 0.18 | 0.19 | 0.2 | 0.21 | 0.22 | 0.23 | 0.24 | 0.25 | 0.26 | 0.27 | 0.28 | 0.29 | 0.3 |
| 40 | 0.15 | 0.17 | 0.22 | 0.27 | 0.33 | 0.40 | 0.47 | 0.54 | 0.62 | 0.67 | 0.74 | 0.79 | 0.84 | 0.89 | 0.93 | 0.94 | 0.96 | 0.97 | 0.98 | 0.99 | 1.00 | 1.00 | 1.00 | 1.00 | 1.00 | 1.00 | 1.00 | 1.00 | 1.00 | 1.00 |
| 60 | 0.13 | 0.18 | 0.27 | 0.39 | 0.52 | 0.65 | 0.76 | 0.86 | 0.91 | 0.96 | 0.98 | 1.00 | 1.00 | 1.00 | 1.00 | 1.00 | 1.00 | 1.00 | 1.00 | 1.00 | 1.00 | 1.00 | 1.00 | 1.00 | 1.00 | 1.00 | 1.00 | 1.00 | 1.00 | 1.00 |
| 80 | 0.13 | 0.26 | 0.45 | 0.66 | 0.83 | 0.93 | 0.98 | 0.99 | 1.00 | 1.00 | 1.00 | 1.00 | 1.00 | 1.00 | 1.00 | 1.00 | 1.00 | 1.00 | 1.00 | 1.00 | 1.00 | 1.00 | 1.00 | 1.00 | 1.00 | 1.00 | 1.00 | 1.00 | 1.00 | 1.00 |
| 100 | 0.17 | 0.40 | 0.68 | 0.88 | 0.97 | 1.00 | 1.00 | 1.00 | 1.00 | 1.00 | 1.00 | 1.00 | 1.00 | 1.00 | 1.00 | 1.00 | 1.00 | 1.00 | 1.00 | 1.00 | 1.00 | 1.00 | 1.00 | 1.00 | 1.00 | 1.00 | 1.00 | 1.00 | 1.00 | 1.00 |
| 120 | 0.21 | 0.54 | 0.85 | 0.98 | 1.00 | 1.00 | 1.00 | 1.00 | 1.00 | 1.00 | 1.00 | 1.00 | 1.00 | 1.00 | 1.00 | 1.00 | 1.00 | 1.00 | 1.00 | 1.00 | 1.00 | 1.00 | 1.00 | 1.00 | 1.00 | 1.00 | 1.00 | 1.00 | 1.00 | 1.00 |
| 140 | 0.26 | 0.72 | 0.96 | 1.00 | 1.00 | 1.00 | 1.00 | 1.00 | 1.00 | 1.00 | 1.00 | 1.00 | 1.00 | 1.00 | 1.00 | 1.00 | 1.00 | 1.00 | 1.00 | 1.00 | 1.00 | 1.00 | 1.00 | 1.00 | 1.00 | 1.00 | 1.00 | 1.00 | 1.00 | 1.00 |
| 160 | 0.38 | 0.87 | 1.00 | 1.00 | 1.00 | 1.00 | 1.00 | 1.00 | 1.00 | 1.00 | 1.00 | 1.00 | 1.00 | 1.00 | 1.00 | 1.00 | 1.00 | 1.00 | 1.00 | 1.00 | 1.00 | 1.00 | 1.00 | 1.00 | 1.00 | 1.00 | 1.00 | 1.00 | 1.00 | 1.00 |
| 180 | 0.49 | 0.96 | 1.00 | 1.00 | 1.00 | 1.00 | 1.00 | 1.00 | 1.00 | 1.00 | 1.00 | 1.00 | 1.00 | 1.00 | 1.00 | 1.00 | 1.00 | 1.00 | 1.00 | 1.00 | 1.00 | 1.00 | 1.00 | 1.00 | 1.00 | 1.00 | 1.00 | 1.00 | 1.00 | 1.00 |
| 200 | 0.58 | 0.99 | 1.00 | 1.00 | 1.00 | 1.00 | 1.00 | 1.00 | 1.00 | 1.00 | 1.00 | 1.00 | 1.00 | 1.00 | 1.00 | 1.00 | 1.00 | 1.00 | 1.00 | 1.00 | 1.00 | 1.00 | 1.00 | 1.00 | 1.00 | 1.00 | 1.00 | 1.00 | 1.00 | 1.00 |
| 220 | 0.69 | 1.00 | 1.00 | 1.00 | 1.00 | 1.00 | 1.00 | 1.00 | 1.00 | 1.00 | 1.00 | 1.00 | 1.00 | 1.00 | 1.00 | 1.00 | 1.00 | 1.00 | 1.00 | 1.00 | 1.00 | 1.00 | 1.00 | 1.00 | 1.00 | 1.00 | 1.00 | 1.00 | 1.00 | 1.00 |
| 240 | 0.78 | 1.00 | 1.00 | 1.00 | 1.00 | 1.00 | 1.00 | 1.00 | 1.00 | 1.00 | 1.00 | 1.00 | 1.00 | 1.00 | 1.00 | 1.00 | 1.00 | 1.00 | 1.00 | 1.00 | 1.00 | 1.00 | 1.00 | 1.00 | 1.00 | 1.00 | 1.00 | 1.00 | 1.00 | 1.00 |
| 260 | 0.88 | 1.00 | 1.00 | 1.00 | 1.00 | 1.00 | 1.00 | 1.00 | 1.00 | 1.00 | 1.00 | 1.00 | 1.00 | 1.00 | 1.00 | 1.00 | 1.00 | 1.00 | 1.00 | 1.00 | 1.00 | 1.00 | 1.00 | 1.00 | 1.00 | 1.00 | 1.00 | 1.00 | 1.00 | 1.00 |
| 280 | 0.93 | 1.00 | 1.00 | 1.00 | 1.00 | 1.00 | 1.00 | 1.00 | 1.00 | 1.00 | 1.00 | 1.00 | 1.00 | 1.00 | 1.00 | 1.00 | 1.00 | 1.00 | 1.00 | 1.00 | 1.00 | 1.00 | 1.00 | 1.00 | 1.00 | 1.00 | 1.00 | 1.00 | 1.00 | 1.00 |
| 300 | 0.96 | 1.00 | 1.00 | 1.00 | 1.00 | 1.00 | 1.00 | 1.00 | 1.00 | 1.00 | 1.00 | 1.00 | 1.00 | 1.00 | 1.00 | 1.00 | 1.00 | 1.00 | 1.00 | 1.00 | 1.00 | 1.00 | 1.00 | 1.00 | 1.00 | 1.00 | 1.00 | 1.00 | 1.00 | 1.00 |
| 320 | 0.99 | 1.00 | 1.00 | 1.00 | 1.00 | 1.00 | 1.00 | 1.00 | 1.00 | 1.00 | 1.00 | 1.00 | 1.00 | 1.00 | 1.00 | 1.00 | 1.00 | 1.00 | 1.00 | 1.00 | 1.00 | 1.00 | 1.00 | 1.00 | 1.00 | 1.00 | 1.00 | 1.00 | 1.00 | 1.00 |
| 340 | 1.00 | 1.00 | 1.00 | 1.00 | 1.00 | 1.00 | 1.00 | 1.00 | 1.00 | 1.00 | 1.00 | 1.00 | 1.00 | 1.00 | 1.00 | 1.00 | 1.00 | 1.00 | 1.00 | 1.00 | 1.00 | 1.00 | 1.00 | 1.00 | 1.00 | 1.00 | 1.00 | 1.00 | 1.00 | 1.00 |
| 360 | 1.00 | 1.00 | 1.00 | 1.00 | 1.00 | 1.00 | 1.00 | 1.00 | 1.00 | 1.00 | 1.00 | 1.00 | 1.00 | 1.00 | 1.00 | 1.00 | 1.00 | 1.00 | 1.00 | 1.00 | 1.00 | 1.00 | 1.00 | 1.00 | 1.00 | 1.00 | 1.00 | 1.00 | 1.00 | 1.00 |
| 380 | 1.00 | 1.00 | 1.00 | 1.00 | 1.00 | 1.00 | 1.00 | 1.00 | 1.00 | 1.00 | 1.00 | 1.00 | 1.00 | 1.00 | 1.00 | 1.00 | 1.00 | 1.00 | 1.00 | 1.00 | 1.00 | 1.00 | 1.00 | 1.00 | 1.00 | 1.00 | 1.00 | 1.00 | 1.00 | 1.00 |
| 400 | 1.00 | 1.00 | 1.00 | 1.00 | 1.00 | 1.00 | 1.00 | 1.00 | 1.00 | 1.00 | 1.00 | 1.00 | 1.00 | 1.00 | 1.00 | 1.00 | 1.00 | 1.00 | 1.00 | 1.00 | 1.00 | 1.00 | 1.00 | 1.00 | 1.00 | 1.00 | 1.00 | 1.00 | 1.00 | 1.00 |

Note: In the simulations it was assumed that there was no underlying trend or covariates which needed to be adjusted for. The intervention was assumed to have occurred at the midpoint of the time series

Table 3: Power to detect a change in trend as a function of time and size of the change in trend, with the presence of AR= 0.9 autocorrelation and standard deviation of the white noise of 0.5.

|  | Effect size for the change in trend | | | | | | | | | | | | | | | | | | | | | | | | | | | | | |
| --- | --- | --- | --- | --- | --- | --- | --- | --- | --- | --- | --- | --- | --- | --- | --- | --- | --- | --- | --- | --- | --- | --- | --- | --- | --- | --- | --- | --- | --- | --- |
| Time | 0.01 | 0.02 | 0.03 | 0.04 | 0.05 | 0.06 | 0.07 | 0.08 | 0.09 | 0.1 | 0.11 | 0.12 | 0.13 | 0.14 | 0.15 | 0.16 | 0.17 | 0.18 | 0.19 | 0.2 | 0.21 | 0.22 | 0.23 | 0.24 | 0.25 | 0.26 | 0.27 | 0.28 | 0.29 | 0.3 |
| 40 | 0.41 | 0.41 | 0.42 | 0.43 | 0.43 | 0.44 | 0.45 | 0.47 | 0.48 | 0.49 | 0.50 | 0.53 | 0.54 | 0.56 | 0.59 | 0.61 | 0.64 | 0.65 | 0.67 | 0.69 | 0.71 | 0.73 | 0.75 | 0.77 | 0.79 | 0.82 | 0.83 | 0.85 | 0.86 | 0.87 |
| 60 | 0.31 | 0.32 | 0.34 | 0.36 | 0.37 | 0.40 | 0.43 | 0.45 | 0.48 | 0.52 | 0.56 | 0.60 | 0.64 | 0.68 | 0.71 | 0.74 | 0.77 | 0.80 | 0.83 | 0.85 | 0.87 | 0.89 | 0.90 | 0.92 | 0.93 | 0.95 | 0.96 | 0.98 | 0.98 | 0.99 |
| 80 | 0.31 | 0.33 | 0.35 | 0.38 | 0.42 | 0.46 | 0.49 | 0.54 | 0.58 | 0.64 | 0.68 | 0.73 | 0.77 | 0.81 | 0.85 | 0.88 | 0.89 | 0.91 | 0.93 | 0.94 | 0.96 | 0.97 | 0.97 | 0.98 | 0.99 | 0.99 | 0.99 | 1.00 | 1.00 | 1.00 |
| 100 | 0.27 | 0.30 | 0.33 | 0.37 | 0.43 | 0.48 | 0.55 | 0.61 | 0.67 | 0.73 | 0.78 | 0.82 | 0.87 | 0.90 | 0.93 | 0.95 | 0.97 | 0.98 | 0.99 | 0.99 | 1.00 | 1.00 | 1.00 | 1.00 | 1.00 | 1.00 | 1.00 | 1.00 | 1.00 | 1.00 |
| 120 | 0.24 | 0.28 | 0.34 | 0.41 | 0.48 | 0.55 | 0.63 | 0.71 | 0.77 | 0.82 | 0.88 | 0.91 | 0.95 | 0.97 | 0.98 | 0.99 | 0.99 | 1.00 | 1.00 | 1.00 | 1.00 | 1.00 | 1.00 | 1.00 | 1.00 | 1.00 | 1.00 | 1.00 | 1.00 | 1.00 |
| 140 | 0.21 | 0.26 | 0.33 | 0.43 | 0.53 | 0.64 | 0.74 | 0.83 | 0.88 | 0.92 | 0.96 | 0.97 | 0.98 | 0.99 | 1.00 | 1.00 | 1.00 | 1.00 | 1.00 | 1.00 | 1.00 | 1.00 | 1.00 | 1.00 | 1.00 | 1.00 | 1.00 | 1.00 | 1.00 | 1.00 |
| 160 | 0.21 | 0.28 | 0.38 | 0.50 | 0.61 | 0.72 | 0.81 | 0.88 | 0.93 | 0.97 | 0.98 | 0.99 | 1.00 | 1.00 | 1.00 | 1.00 | 1.00 | 1.00 | 1.00 | 1.00 | 1.00 | 1.00 | 1.00 | 1.00 | 1.00 | 1.00 | 1.00 | 1.00 | 1.00 | 1.00 |
| 180 | 0.23 | 0.33 | 0.44 | 0.57 | 0.71 | 0.82 | 0.91 | 0.95 | 0.98 | 0.99 | 1.00 | 1.00 | 1.00 | 1.00 | 1.00 | 1.00 | 1.00 | 1.00 | 1.00 | 1.00 | 1.00 | 1.00 | 1.00 | 1.00 | 1.00 | 1.00 | 1.00 | 1.00 | 1.00 | 1.00 |
| 200 | 0.20 | 0.30 | 0.45 | 0.62 | 0.76 | 0.88 | 0.94 | 0.97 | 0.99 | 1.00 | 1.00 | 1.00 | 1.00 | 1.00 | 1.00 | 1.00 | 1.00 | 1.00 | 1.00 | 1.00 | 1.00 | 1.00 | 1.00 | 1.00 | 1.00 | 1.00 | 1.00 | 1.00 | 1.00 | 1.00 |
| 220 | 0.20 | 0.31 | 0.50 | 0.70 | 0.84 | 0.92 | 0.97 | 0.99 | 1.00 | 1.00 | 1.00 | 1.00 | 1.00 | 1.00 | 1.00 | 1.00 | 1.00 | 1.00 | 1.00 | 1.00 | 1.00 | 1.00 | 1.00 | 1.00 | 1.00 | 1.00 | 1.00 | 1.00 | 1.00 | 1.00 |
| 240 | 0.20 | 0.37 | 0.57 | 0.77 | 0.90 | 0.96 | 0.99 | 1.00 | 1.00 | 1.00 | 1.00 | 1.00 | 1.00 | 1.00 | 1.00 | 1.00 | 1.00 | 1.00 | 1.00 | 1.00 | 1.00 | 1.00 | 1.00 | 1.00 | 1.00 | 1.00 | 1.00 | 1.00 | 1.00 | 1.00 |
| 260 | 0.24 | 0.43 | 0.66 | 0.85 | 0.95 | 0.99 | 1.00 | 1.00 | 1.00 | 1.00 | 1.00 | 1.00 | 1.00 | 1.00 | 1.00 | 1.00 | 1.00 | 1.00 | 1.00 | 1.00 | 1.00 | 1.00 | 1.00 | 1.00 | 1.00 | 1.00 | 1.00 | 1.00 | 1.00 | 1.00 |
| 280 | 0.25 | 0.46 | 0.72 | 0.88 | 0.97 | 1.00 | 1.00 | 1.00 | 1.00 | 1.00 | 1.00 | 1.00 | 1.00 | 1.00 | 1.00 | 1.00 | 1.00 | 1.00 | 1.00 | 1.00 | 1.00 | 1.00 | 1.00 | 1.00 | 1.00 | 1.00 | 1.00 | 1.00 | 1.00 | 1.00 |
| 300 | 0.24 | 0.50 | 0.78 | 0.93 | 0.98 | 1.00 | 1.00 | 1.00 | 1.00 | 1.00 | 1.00 | 1.00 | 1.00 | 1.00 | 1.00 | 1.00 | 1.00 | 1.00 | 1.00 | 1.00 | 1.00 | 1.00 | 1.00 | 1.00 | 1.00 | 1.00 | 1.00 | 1.00 | 1.00 | 1.00 |
| 320 | 0.27 | 0.56 | 0.82 | 0.96 | 1.00 | 1.00 | 1.00 | 1.00 | 1.00 | 1.00 | 1.00 | 1.00 | 1.00 | 1.00 | 1.00 | 1.00 | 1.00 | 1.00 | 1.00 | 1.00 | 1.00 | 1.00 | 1.00 | 1.00 | 1.00 | 1.00 | 1.00 | 1.00 | 1.00 | 1.00 |
| 340 | 0.28 | 0.61 | 0.88 | 0.97 | 1.00 | 1.00 | 1.00 | 1.00 | 1.00 | 1.00 | 1.00 | 1.00 | 1.00 | 1.00 | 1.00 | 1.00 | 1.00 | 1.00 | 1.00 | 1.00 | 1.00 | 1.00 | 1.00 | 1.00 | 1.00 | 1.00 | 1.00 | 1.00 | 1.00 | 1.00 |
| 360 | 0.27 | 0.63 | 0.91 | 0.99 | 1.00 | 1.00 | 1.00 | 1.00 | 1.00 | 1.00 | 1.00 | 1.00 | 1.00 | 1.00 | 1.00 | 1.00 | 1.00 | 1.00 | 1.00 | 1.00 | 1.00 | 1.00 | 1.00 | 1.00 | 1.00 | 1.00 | 1.00 | 1.00 | 1.00 | 1.00 |
| 380 | 0.34 | 0.73 | 0.95 | 1.00 | 1.00 | 1.00 | 1.00 | 1.00 | 1.00 | 1.00 | 1.00 | 1.00 | 1.00 | 1.00 | 1.00 | 1.00 | 1.00 | 1.00 | 1.00 | 1.00 | 1.00 | 1.00 | 1.00 | 1.00 | 1.00 | 1.00 | 1.00 | 1.00 | 1.00 | 1.00 |
| 400 | 0.35 | 0.75 | 0.98 | 1.00 | 1.00 | 1.00 | 1.00 | 1.00 | 1.00 | 1.00 | 1.00 | 1.00 | 1.00 | 1.00 | 1.00 | 1.00 | 1.00 | 1.00 | 1.00 | 1.00 | 1.00 | 1.00 | 1.00 | 1.00 | 1.00 | 1.00 | 1.00 | 1.00 | 1.00 | 1.00 |

Note: In the simulations it was assumed that there was no underlying trend or covariates which needed to be adjusted for. The intervention was assumed to have occurred at the midpoint of the time series.

Table 4: Power to detect a change in trend as a function of time and size of the change in trend, with the presence of AR= 0.1 autocorrelation and standard deviation of the white noise of 1.

|  | Effect size for the change in trend | | | | | | | | | | | | | | | | | | | | | | | | | | | | | |
| --- | --- | --- | --- | --- | --- | --- | --- | --- | --- | --- | --- | --- | --- | --- | --- | --- | --- | --- | --- | --- | --- | --- | --- | --- | --- | --- | --- | --- | --- | --- |
| Time | 0.01 | 0.02 | 0.03 | 0.04 | 0.05 | 0.06 | 0.07 | 0.08 | 0.09 | 0.1 | 0.11 | 0.12 | 0.13 | 0.14 | 0.15 | 0.16 | 0.17 | 0.18 | 0.19 | 0.2 | 0.21 | 0.22 | 0.23 | 0.24 | 0.25 | 0.26 | 0.27 | 0.28 | 0.29 | 0.3 |
| 40 | 0.09 | 0.11 | 0.13 | 0.16 | 0.20 | 0.25 | 0.28 | 0.33 | 0.38 | 0.45 | 0.51 | 0.57 | 0.64 | 0.70 | 0.76 | 0.82 | 0.85 | 0.88 | 0.92 | 0.94 | 0.96 | 0.97 | 0.98 | 0.99 | 0.99 | 0.99 | 0.99 | 1.00 | 1.00 | 1.00 |
| 60 | 0.09 | 0.13 | 0.20 | 0.28 | 0.38 | 0.51 | 0.63 | 0.74 | 0.82 | 0.90 | 0.94 | 0.96 | 0.98 | 0.99 | 1.00 | 1.00 | 1.00 | 1.00 | 1.00 | 1.00 | 1.00 | 1.00 | 1.00 | 1.00 | 1.00 | 1.00 | 1.00 | 1.00 | 1.00 | 1.00 |
| 80 | 0.11 | 0.20 | 0.34 | 0.53 | 0.69 | 0.83 | 0.93 | 0.97 | 0.99 | 1.00 | 1.00 | 1.00 | 1.00 | 1.00 | 1.00 | 1.00 | 1.00 | 1.00 | 1.00 | 1.00 | 1.00 | 1.00 | 1.00 | 1.00 | 1.00 | 1.00 | 1.00 | 1.00 | 1.00 | 1.00 |
| 100 | 0.11 | 0.31 | 0.55 | 0.76 | 0.92 | 0.98 | 1.00 | 1.00 | 1.00 | 1.00 | 1.00 | 1.00 | 1.00 | 1.00 | 1.00 | 1.00 | 1.00 | 1.00 | 1.00 | 1.00 | 1.00 | 1.00 | 1.00 | 1.00 | 1.00 | 1.00 | 1.00 | 1.00 | 1.00 | 1.00 |
| 120 | 0.14 | 0.43 | 0.74 | 0.94 | 0.99 | 1.00 | 1.00 | 1.00 | 1.00 | 1.00 | 1.00 | 1.00 | 1.00 | 1.00 | 1.00 | 1.00 | 1.00 | 1.00 | 1.00 | 1.00 | 1.00 | 1.00 | 1.00 | 1.00 | 1.00 | 1.00 | 1.00 | 1.00 | 1.00 | 1.00 |
| 140 | 0.24 | 0.63 | 0.91 | 0.99 | 1.00 | 1.00 | 1.00 | 1.00 | 1.00 | 1.00 | 1.00 | 1.00 | 1.00 | 1.00 | 1.00 | 1.00 | 1.00 | 1.00 | 1.00 | 1.00 | 1.00 | 1.00 | 1.00 | 1.00 | 1.00 | 1.00 | 1.00 | 1.00 | 1.00 | 1.00 |
| 160 | 0.29 | 0.76 | 0.98 | 1.00 | 1.00 | 1.00 | 1.00 | 1.00 | 1.00 | 1.00 | 1.00 | 1.00 | 1.00 | 1.00 | 1.00 | 1.00 | 1.00 | 1.00 | 1.00 | 1.00 | 1.00 | 1.00 | 1.00 | 1.00 | 1.00 | 1.00 | 1.00 | 1.00 | 1.00 | 1.00 |
| 180 | 0.34 | 0.89 | 1.00 | 1.00 | 1.00 | 1.00 | 1.00 | 1.00 | 1.00 | 1.00 | 1.00 | 1.00 | 1.00 | 1.00 | 1.00 | 1.00 | 1.00 | 1.00 | 1.00 | 1.00 | 1.00 | 1.00 | 1.00 | 1.00 | 1.00 | 1.00 | 1.00 | 1.00 | 1.00 | 1.00 |
| 200 | 0.48 | 0.95 | 1.00 | 1.00 | 1.00 | 1.00 | 1.00 | 1.00 | 1.00 | 1.00 | 1.00 | 1.00 | 1.00 | 1.00 | 1.00 | 1.00 | 1.00 | 1.00 | 1.00 | 1.00 | 1.00 | 1.00 | 1.00 | 1.00 | 1.00 | 1.00 | 1.00 | 1.00 | 1.00 | 1.00 |
| 220 | 0.59 | 0.99 | 1.00 | 1.00 | 1.00 | 1.00 | 1.00 | 1.00 | 1.00 | 1.00 | 1.00 | 1.00 | 1.00 | 1.00 | 1.00 | 1.00 | 1.00 | 1.00 | 1.00 | 1.00 | 1.00 | 1.00 | 1.00 | 1.00 | 1.00 | 1.00 | 1.00 | 1.00 | 1.00 | 1.00 |
| 240 | 0.67 | 1.00 | 1.00 | 1.00 | 1.00 | 1.00 | 1.00 | 1.00 | 1.00 | 1.00 | 1.00 | 1.00 | 1.00 | 1.00 | 1.00 | 1.00 | 1.00 | 1.00 | 1.00 | 1.00 | 1.00 | 1.00 | 1.00 | 1.00 | 1.00 | 1.00 | 1.00 | 1.00 | 1.00 | 1.00 |
| 260 | 0.81 | 1.00 | 1.00 | 1.00 | 1.00 | 1.00 | 1.00 | 1.00 | 1.00 | 1.00 | 1.00 | 1.00 | 1.00 | 1.00 | 1.00 | 1.00 | 1.00 | 1.00 | 1.00 | 1.00 | 1.00 | 1.00 | 1.00 | 1.00 | 1.00 | 1.00 | 1.00 | 1.00 | 1.00 | 1.00 |
| 280 | 0.86 | 1.00 | 1.00 | 1.00 | 1.00 | 1.00 | 1.00 | 1.00 | 1.00 | 1.00 | 1.00 | 1.00 | 1.00 | 1.00 | 1.00 | 1.00 | 1.00 | 1.00 | 1.00 | 1.00 | 1.00 | 1.00 | 1.00 | 1.00 | 1.00 | 1.00 | 1.00 | 1.00 | 1.00 | 1.00 |
| 300 | 0.92 | 1.00 | 1.00 | 1.00 | 1.00 | 1.00 | 1.00 | 1.00 | 1.00 | 1.00 | 1.00 | 1.00 | 1.00 | 1.00 | 1.00 | 1.00 | 1.00 | 1.00 | 1.00 | 1.00 | 1.00 | 1.00 | 1.00 | 1.00 | 1.00 | 1.00 | 1.00 | 1.00 | 1.00 | 1.00 |
| 320 | 0.98 | 1.00 | 1.00 | 1.00 | 1.00 | 1.00 | 1.00 | 1.00 | 1.00 | 1.00 | 1.00 | 1.00 | 1.00 | 1.00 | 1.00 | 1.00 | 1.00 | 1.00 | 1.00 | 1.00 | 1.00 | 1.00 | 1.00 | 1.00 | 1.00 | 1.00 | 1.00 | 1.00 | 1.00 | 1.00 |
| 340 | 0.98 | 1.00 | 1.00 | 1.00 | 1.00 | 1.00 | 1.00 | 1.00 | 1.00 | 1.00 | 1.00 | 1.00 | 1.00 | 1.00 | 1.00 | 1.00 | 1.00 | 1.00 | 1.00 | 1.00 | 1.00 | 1.00 | 1.00 | 1.00 | 1.00 | 1.00 | 1.00 | 1.00 | 1.00 | 1.00 |
| 360 | 1.00 | 1.00 | 1.00 | 1.00 | 1.00 | 1.00 | 1.00 | 1.00 | 1.00 | 1.00 | 1.00 | 1.00 | 1.00 | 1.00 | 1.00 | 1.00 | 1.00 | 1.00 | 1.00 | 1.00 | 1.00 | 1.00 | 1.00 | 1.00 | 1.00 | 1.00 | 1.00 | 1.00 | 1.00 | 1.00 |
| 380 | 1.00 | 1.00 | 1.00 | 1.00 | 1.00 | 1.00 | 1.00 | 1.00 | 1.00 | 1.00 | 1.00 | 1.00 | 1.00 | 1.00 | 1.00 | 1.00 | 1.00 | 1.00 | 1.00 | 1.00 | 1.00 | 1.00 | 1.00 | 1.00 | 1.00 | 1.00 | 1.00 | 1.00 | 1.00 | 1.00 |
| 400 | 1.00 | 1.00 | 1.00 | 1.00 | 1.00 | 1.00 | 1.00 | 1.00 | 1.00 | 1.00 | 1.00 | 1.00 | 1.00 | 1.00 | 1.00 | 1.00 | 1.00 | 1.00 | 1.00 | 1.00 | 1.00 | 1.00 | 1.00 | 1.00 | 1.00 | 1.00 | 1.00 | 1.00 | 1.00 | 1.00 |

Note: In the simulations it was assumed that there was no underlying trend or covariates which needed to be adjusted for. The intervention was assumed to have occurred at the midpoint of the time series.

Table 5: Power to detect a change in trend as a function of time and size of the change in trend, with the presence of AR= 0.9 autocorrelation and standard deviation of the white noise of 1.

|  | Effect size for the change in trend | | | | | | | | | | | | | | | | | | | | | | | | | | | | | |
| --- | --- | --- | --- | --- | --- | --- | --- | --- | --- | --- | --- | --- | --- | --- | --- | --- | --- | --- | --- | --- | --- | --- | --- | --- | --- | --- | --- | --- | --- | --- |
| Time | 0.01 | 0.02 | 0.03 | 0.04 | 0.05 | 0.06 | 0.07 | 0.08 | 0.09 | 0.1 | 0.11 | 0.12 | 0.13 | 0.14 | 0.15 | 0.16 | 0.17 | 0.18 | 0.19 | 0.2 | 0.21 | 0.22 | 0.23 | 0.24 | 0.25 | 0.26 | 0.27 | 0.28 | 0.29 | 0.3 |
| 40 | 0.39 | 0.40 | 0.41 | 0.41 | 0.41 | 0.41 | 0.42 | 0.42 | 0.43 | 0.43 | 0.43 | 0.42 | 0.42 | 0.43 | 0.44 | 0.45 | 0.45 | 0.46 | 0.47 | 0.48 | 0.49 | 0.49 | 0.51 | 0.51 | 0.52 | 0.54 | 0.55 | 0.56 | 0.57 | 0.58 |
| 60 | 0.28 | 0.28 | 0.29 | 0.30 | 0.30 | 0.31 | 0.32 | 0.33 | 0.34 | 0.35 | 0.38 | 0.39 | 0.40 | 0.41 | 0.43 | 0.44 | 0.47 | 0.49 | 0.51 | 0.54 | 0.56 | 0.57 | 0.59 | 0.62 | 0.64 | 0.66 | 0.68 | 0.71 | 0.72 | 0.73 |
| 80 | 0.28 | 0.29 | 0.30 | 0.30 | 0.32 | 0.33 | 0.35 | 0.37 | 0.39 | 0.41 | 0.43 | 0.45 | 0.46 | 0.49 | 0.51 | 0.54 | 0.57 | 0.60 | 0.62 | 0.65 | 0.68 | 0.69 | 0.71 | 0.75 | 0.76 | 0.79 | 0.81 | 0.82 | 0.84 | 0.86 |
| 100 | 0.24 | 0.24 | 0.25 | 0.27 | 0.29 | 0.32 | 0.35 | 0.36 | 0.39 | 0.42 | 0.44 | 0.48 | 0.52 | 0.56 | 0.59 | 0.63 | 0.66 | 0.69 | 0.72 | 0.75 | 0.78 | 0.80 | 0.82 | 0.85 | 0.87 | 0.89 | 0.91 | 0.93 | 0.94 | 0.95 |
| 120 | 0.20 | 0.22 | 0.23 | 0.26 | 0.30 | 0.32 | 0.36 | 0.40 | 0.43 | 0.47 | 0.50 | 0.56 | 0.60 | 0.64 | 0.68 | 0.72 | 0.76 | 0.80 | 0.82 | 0.85 | 0.87 | 0.90 | 0.93 | 0.94 | 0.95 | 0.96 | 0.97 | 0.98 | 0.98 | 0.99 |
| 140 | 0.19 | 0.20 | 0.21 | 0.24 | 0.28 | 0.33 | 0.39 | 0.44 | 0.50 | 0.54 | 0.60 | 0.66 | 0.71 | 0.75 | 0.81 | 0.85 | 0.88 | 0.90 | 0.92 | 0.94 | 0.96 | 0.97 | 0.98 | 0.99 | 0.99 | 0.99 | 0.99 | 1.00 | 1.00 | 1.00 |
| 160 | 0.19 | 0.20 | 0.23 | 0.27 | 0.32 | 0.39 | 0.43 | 0.50 | 0.56 | 0.63 | 0.70 | 0.73 | 0.77 | 0.83 | 0.88 | 0.91 | 0.93 | 0.95 | 0.97 | 0.98 | 0.99 | 0.99 | 0.99 | 1.00 | 1.00 | 1.00 | 1.00 | 1.00 | 1.00 | 1.00 |
| 180 | 0.20 | 0.22 | 0.26 | 0.33 | 0.39 | 0.46 | 0.52 | 0.58 | 0.65 | 0.72 | 0.79 | 0.84 | 0.89 | 0.92 | 0.95 | 0.96 | 0.97 | 0.98 | 0.99 | 0.99 | 1.00 | 1.00 | 1.00 | 1.00 | 1.00 | 1.00 | 1.00 | 1.00 | 1.00 | 1.00 |
| 200 | 0.17 | 0.19 | 0.24 | 0.29 | 0.37 | 0.45 | 0.55 | 0.64 | 0.72 | 0.79 | 0.84 | 0.89 | 0.93 | 0.95 | 0.97 | 0.98 | 0.99 | 1.00 | 1.00 | 1.00 | 1.00 | 1.00 | 1.00 | 1.00 | 1.00 | 1.00 | 1.00 | 1.00 | 1.00 | 1.00 |
| 220 | 0.17 | 0.19 | 0.25 | 0.32 | 0.40 | 0.50 | 0.61 | 0.71 | 0.79 | 0.86 | 0.91 | 0.94 | 0.97 | 0.98 | 0.99 | 1.00 | 1.00 | 1.00 | 1.00 | 1.00 | 1.00 | 1.00 | 1.00 | 1.00 | 1.00 | 1.00 | 1.00 | 1.00 | 1.00 | 1.00 |
| 240 | 0.15 | 0.18 | 0.27 | 0.37 | 0.47 | 0.58 | 0.68 | 0.79 | 0.87 | 0.91 | 0.95 | 0.98 | 0.99 | 0.99 | 1.00 | 1.00 | 1.00 | 1.00 | 1.00 | 1.00 | 1.00 | 1.00 | 1.00 | 1.00 | 1.00 | 1.00 | 1.00 | 1.00 | 1.00 | 1.00 |
| 260 | 0.17 | 0.22 | 0.32 | 0.44 | 0.56 | 0.68 | 0.79 | 0.86 | 0.92 | 0.96 | 0.98 | 0.99 | 1.00 | 1.00 | 1.00 | 1.00 | 1.00 | 1.00 | 1.00 | 1.00 | 1.00 | 1.00 | 1.00 | 1.00 | 1.00 | 1.00 | 1.00 | 1.00 | 1.00 | 1.00 |
| 280 | 0.16 | 0.24 | 0.34 | 0.48 | 0.61 | 0.75 | 0.84 | 0.90 | 0.95 | 0.98 | 0.99 | 1.00 | 1.00 | 1.00 | 1.00 | 1.00 | 1.00 | 1.00 | 1.00 | 1.00 | 1.00 | 1.00 | 1.00 | 1.00 | 1.00 | 1.00 | 1.00 | 1.00 | 1.00 | 1.00 |
| 300 | 0.15 | 0.23 | 0.36 | 0.50 | 0.66 | 0.80 | 0.88 | 0.95 | 0.97 | 0.99 | 1.00 | 1.00 | 1.00 | 1.00 | 1.00 | 1.00 | 1.00 | 1.00 | 1.00 | 1.00 | 1.00 | 1.00 | 1.00 | 1.00 | 1.00 | 1.00 | 1.00 | 1.00 | 1.00 | 1.00 |
| 320 | 0.15 | 0.27 | 0.39 | 0.56 | 0.72 | 0.84 | 0.93 | 0.97 | 0.99 | 1.00 | 1.00 | 1.00 | 1.00 | 1.00 | 1.00 | 1.00 | 1.00 | 1.00 | 1.00 | 1.00 | 1.00 | 1.00 | 1.00 | 1.00 | 1.00 | 1.00 | 1.00 | 1.00 | 1.00 | 1.00 |
| 340 | 0.16 | 0.27 | 0.42 | 0.62 | 0.79 | 0.89 | 0.95 | 0.98 | 0.99 | 1.00 | 1.00 | 1.00 | 1.00 | 1.00 | 1.00 | 1.00 | 1.00 | 1.00 | 1.00 | 1.00 | 1.00 | 1.00 | 1.00 | 1.00 | 1.00 | 1.00 | 1.00 | 1.00 | 1.00 | 1.00 |
| 360 | 0.16 | 0.27 | 0.45 | 0.65 | 0.82 | 0.93 | 0.97 | 0.99 | 1.00 | 1.00 | 1.00 | 1.00 | 1.00 | 1.00 | 1.00 | 1.00 | 1.00 | 1.00 | 1.00 | 1.00 | 1.00 | 1.00 | 1.00 | 1.00 | 1.00 | 1.00 | 1.00 | 1.00 | 1.00 | 1.00 |
| 380 | 0.18 | 0.35 | 0.56 | 0.74 | 0.88 | 0.97 | 0.99 | 1.00 | 1.00 | 1.00 | 1.00 | 1.00 | 1.00 | 1.00 | 1.00 | 1.00 | 1.00 | 1.00 | 1.00 | 1.00 | 1.00 | 1.00 | 1.00 | 1.00 | 1.00 | 1.00 | 1.00 | 1.00 | 1.00 | 1.00 |
| 400 | 0.17 | 0.36 | 0.57 | 0.78 | 0.90 | 0.98 | 1.00 | 1.00 | 1.00 | 1.00 | 1.00 | 1.00 | 1.00 | 1.00 | 1.00 | 1.00 | 1.00 | 1.00 | 1.00 | 1.00 | 1.00 | 1.00 | 1.00 | 1.00 | 1.00 | 1.00 | 1.00 | 1.00 | 1.00 | 1.00 |

Note: In the simulations it was assumed that there was no underlying trend or covariates which needed to be adjusted for. The intervention was assumed to have occurred at the midpoint of the time series.

Table 6: Power to detect a change in trend as a function of time and size of the change in trend, with the presence of AR= 0.1 autocorrelation and standard deviation of the white noise of 2.

|  | Effect size for the change in trend | | | | | | | | | | | | | | | | | | | | | | | | | | | | | |
| --- | --- | --- | --- | --- | --- | --- | --- | --- | --- | --- | --- | --- | --- | --- | --- | --- | --- | --- | --- | --- | --- | --- | --- | --- | --- | --- | --- | --- | --- | --- |
| Time | 0.01 | 0.02 | 0.03 | 0.04 | 0.05 | 0.06 | 0.07 | 0.08 | 0.09 | 0.1 | 0.11 | 0.12 | 0.13 | 0.14 | 0.15 | 0.16 | 0.17 | 0.18 | 0.19 | 0.2 | 0.21 | 0.22 | 0.23 | 0.24 | 0.25 | 0.26 | 0.27 | 0.28 | 0.29 | 0.3 |
| 40 | 0.10 | 0.09 | 0.09 | 0.11 | 0.12 | 0.13 | 0.15 | 0.16 | 0.18 | 0.20 | 0.23 | 0.25 | 0.26 | 0.28 | 0.31 | 0.33 | 0.35 | 0.38 | 0.42 | 0.45 | 0.48 | 0.51 | 0.54 | 0.57 | 0.61 | 0.64 | 0.67 | 0.70 | 0.73 | 0.76 |
| 60 | 0.08 | 0.09 | 0.11 | 0.13 | 0.16 | 0.20 | 0.23 | 0.28 | 0.32 | 0.38 | 0.44 | 0.51 | 0.57 | 0.63 | 0.69 | 0.74 | 0.78 | 0.82 | 0.87 | 0.90 | 0.92 | 0.94 | 0.96 | 0.96 | 0.98 | 0.98 | 0.99 | 0.99 | 0.99 | 1.00 |
| 80 | 0.09 | 0.11 | 0.15 | 0.20 | 0.27 | 0.34 | 0.42 | 0.53 | 0.61 | 0.69 | 0.77 | 0.83 | 0.89 | 0.93 | 0.95 | 0.97 | 0.98 | 0.99 | 1.00 | 1.00 | 1.00 | 1.00 | 1.00 | 1.00 | 1.00 | 1.00 | 1.00 | 1.00 | 1.00 | 1.00 |
| 100 | 0.07 | 0.11 | 0.19 | 0.31 | 0.42 | 0.55 | 0.67 | 0.76 | 0.85 | 0.92 | 0.96 | 0.98 | 0.99 | 1.00 | 1.00 | 1.00 | 1.00 | 1.00 | 1.00 | 1.00 | 1.00 | 1.00 | 1.00 | 1.00 | 1.00 | 1.00 | 1.00 | 1.00 | 1.00 | 1.00 |
| 120 | 0.09 | 0.14 | 0.28 | 0.43 | 0.58 | 0.74 | 0.86 | 0.94 | 0.98 | 0.99 | 1.00 | 1.00 | 1.00 | 1.00 | 1.00 | 1.00 | 1.00 | 1.00 | 1.00 | 1.00 | 1.00 | 1.00 | 1.00 | 1.00 | 1.00 | 1.00 | 1.00 | 1.00 | 1.00 | 1.00 |
| 140 | 0.12 | 0.24 | 0.41 | 0.63 | 0.80 | 0.91 | 0.97 | 0.99 | 1.00 | 1.00 | 1.00 | 1.00 | 1.00 | 1.00 | 1.00 | 1.00 | 1.00 | 1.00 | 1.00 | 1.00 | 1.00 | 1.00 | 1.00 | 1.00 | 1.00 | 1.00 | 1.00 | 1.00 | 1.00 | 1.00 |
| 160 | 0.12 | 0.29 | 0.55 | 0.76 | 0.93 | 0.98 | 1.00 | 1.00 | 1.00 | 1.00 | 1.00 | 1.00 | 1.00 | 1.00 | 1.00 | 1.00 | 1.00 | 1.00 | 1.00 | 1.00 | 1.00 | 1.00 | 1.00 | 1.00 | 1.00 | 1.00 | 1.00 | 1.00 | 1.00 | 1.00 |
| 180 | 0.13 | 0.34 | 0.67 | 0.89 | 0.99 | 1.00 | 1.00 | 1.00 | 1.00 | 1.00 | 1.00 | 1.00 | 1.00 | 1.00 | 1.00 | 1.00 | 1.00 | 1.00 | 1.00 | 1.00 | 1.00 | 1.00 | 1.00 | 1.00 | 1.00 | 1.00 | 1.00 | 1.00 | 1.00 | 1.00 |
| 200 | 0.15 | 0.48 | 0.79 | 0.95 | 1.00 | 1.00 | 1.00 | 1.00 | 1.00 | 1.00 | 1.00 | 1.00 | 1.00 | 1.00 | 1.00 | 1.00 | 1.00 | 1.00 | 1.00 | 1.00 | 1.00 | 1.00 | 1.00 | 1.00 | 1.00 | 1.00 | 1.00 | 1.00 | 1.00 | 1.00 |
| 220 | 0.20 | 0.59 | 0.90 | 0.99 | 1.00 | 1.00 | 1.00 | 1.00 | 1.00 | 1.00 | 1.00 | 1.00 | 1.00 | 1.00 | 1.00 | 1.00 | 1.00 | 1.00 | 1.00 | 1.00 | 1.00 | 1.00 | 1.00 | 1.00 | 1.00 | 1.00 | 1.00 | 1.00 | 1.00 | 1.00 |
| 240 | 0.23 | 0.67 | 0.95 | 1.00 | 1.00 | 1.00 | 1.00 | 1.00 | 1.00 | 1.00 | 1.00 | 1.00 | 1.00 | 1.00 | 1.00 | 1.00 | 1.00 | 1.00 | 1.00 | 1.00 | 1.00 | 1.00 | 1.00 | 1.00 | 1.00 | 1.00 | 1.00 | 1.00 | 1.00 | 1.00 |
| 260 | 0.30 | 0.81 | 0.98 | 1.00 | 1.00 | 1.00 | 1.00 | 1.00 | 1.00 | 1.00 | 1.00 | 1.00 | 1.00 | 1.00 | 1.00 | 1.00 | 1.00 | 1.00 | 1.00 | 1.00 | 1.00 | 1.00 | 1.00 | 1.00 | 1.00 | 1.00 | 1.00 | 1.00 | 1.00 | 1.00 |
| 280 | 0.32 | 0.86 | 1.00 | 1.00 | 1.00 | 1.00 | 1.00 | 1.00 | 1.00 | 1.00 | 1.00 | 1.00 | 1.00 | 1.00 | 1.00 | 1.00 | 1.00 | 1.00 | 1.00 | 1.00 | 1.00 | 1.00 | 1.00 | 1.00 | 1.00 | 1.00 | 1.00 | 1.00 | 1.00 | 1.00 |
| 300 | 0.43 | 0.92 | 1.00 | 1.00 | 1.00 | 1.00 | 1.00 | 1.00 | 1.00 | 1.00 | 1.00 | 1.00 | 1.00 | 1.00 | 1.00 | 1.00 | 1.00 | 1.00 | 1.00 | 1.00 | 1.00 | 1.00 | 1.00 | 1.00 | 1.00 | 1.00 | 1.00 | 1.00 | 1.00 | 1.00 |
| 320 | 0.52 | 0.98 | 1.00 | 1.00 | 1.00 | 1.00 | 1.00 | 1.00 | 1.00 | 1.00 | 1.00 | 1.00 | 1.00 | 1.00 | 1.00 | 1.00 | 1.00 | 1.00 | 1.00 | 1.00 | 1.00 | 1.00 | 1.00 | 1.00 | 1.00 | 1.00 | 1.00 | 1.00 | 1.00 | 1.00 |
| 340 | 0.51 | 0.98 | 1.00 | 1.00 | 1.00 | 1.00 | 1.00 | 1.00 | 1.00 | 1.00 | 1.00 | 1.00 | 1.00 | 1.00 | 1.00 | 1.00 | 1.00 | 1.00 | 1.00 | 1.00 | 1.00 | 1.00 | 1.00 | 1.00 | 1.00 | 1.00 | 1.00 | 1.00 | 1.00 | 1.00 |
| 360 | 0.63 | 1.00 | 1.00 | 1.00 | 1.00 | 1.00 | 1.00 | 1.00 | 1.00 | 1.00 | 1.00 | 1.00 | 1.00 | 1.00 | 1.00 | 1.00 | 1.00 | 1.00 | 1.00 | 1.00 | 1.00 | 1.00 | 1.00 | 1.00 | 1.00 | 1.00 | 1.00 | 1.00 | 1.00 | 1.00 |
| 380 | 0.68 | 1.00 | 1.00 | 1.00 | 1.00 | 1.00 | 1.00 | 1.00 | 1.00 | 1.00 | 1.00 | 1.00 | 1.00 | 1.00 | 1.00 | 1.00 | 1.00 | 1.00 | 1.00 | 1.00 | 1.00 | 1.00 | 1.00 | 1.00 | 1.00 | 1.00 | 1.00 | 1.00 | 1.00 | 1.00 |
| 400 | 0.73 | 1.00 | 1.00 | 1.00 | 1.00 | 1.00 | 1.00 | 1.00 | 1.00 | 1.00 | 1.00 | 1.00 | 1.00 | 1.00 | 1.00 | 1.00 | 1.00 | 1.00 | 1.00 | 1.00 | 1.00 | 1.00 | 1.00 | 1.00 | 1.00 | 1.00 | 1.00 | 1.00 | 1.00 | 1.00 |

Note: In the simulations it was assumed that there was no underlying trend or covariates which needed to be adjusted for. The intervention was assumed to have occurred at the midpoint of the time series.

Table 7: Power to detect a change in trend as a function of time and size of the change in trend, with the presence of AR= 0.5 autocorrelation and standard deviation of the white noise of 2.

|  | Effect size for the change in trend | | | | | | | | | | | | | | | | | | | | | | | | | | | | | |
| --- | --- | --- | --- | --- | --- | --- | --- | --- | --- | --- | --- | --- | --- | --- | --- | --- | --- | --- | --- | --- | --- | --- | --- | --- | --- | --- | --- | --- | --- | --- |
| Time | 0.01 | 0.02 | 0.03 | 0.04 | 0.05 | 0.06 | 0.07 | 0.08 | 0.09 | 0.1 | 0.11 | 0.12 | 0.13 | 0.14 | 0.15 | 0.16 | 0.17 | 0.18 | 0.19 | 0.2 | 0.21 | 0.22 | 0.23 | 0.24 | 0.25 | 0.26 | 0.27 | 0.28 | 0.29 | 0.3 |
| 40 | 0.14 | 0.14 | 0.14 | 0.15 | 0.15 | 0.16 | 0.16 | 0.17 | 0.18 | 0.20 | 0.20 | 0.22 | 0.23 | 0.24 | 0.25 | 0.26 | 0.28 | 0.30 | 0.31 | 0.33 | 0.35 | 0.36 | 0.38 | 0.40 | 0.42 | 0.44 | 0.46 | 0.47 | 0.49 | 0.50 |
| 60 | 0.13 | 0.13 | 0.13 | 0.13 | 0.13 | 0.14 | 0.16 | 0.18 | 0.20 | 0.23 | 0.24 | 0.27 | 0.30 | 0.32 | 0.34 | 0.39 | 0.42 | 0.45 | 0.49 | 0.52 | 0.56 | 0.59 | 0.62 | 0.65 | 0.68 | 0.71 | 0.73 | 0.76 | 0.79 | 0.82 |
| 80 | 0.08 | 0.10 | 0.12 | 0.13 | 0.16 | 0.20 | 0.22 | 0.26 | 0.31 | 0.35 | 0.39 | 0.45 | 0.51 | 0.56 | 0.61 | 0.66 | 0.71 | 0.75 | 0.78 | 0.83 | 0.86 | 0.88 | 0.90 | 0.93 | 0.95 | 0.96 | 0.97 | 0.98 | 0.98 | 0.99 |
| 100 | 0.09 | 0.10 | 0.13 | 0.17 | 0.22 | 0.28 | 0.33 | 0.41 | 0.47 | 0.55 | 0.61 | 0.68 | 0.73 | 0.79 | 0.85 | 0.88 | 0.91 | 0.93 | 0.96 | 0.97 | 0.98 | 0.99 | 0.99 | 1.00 | 1.00 | 1.00 | 1.00 | 1.00 | 1.00 | 1.00 |
| 120 | 0.09 | 0.12 | 0.16 | 0.21 | 0.28 | 0.37 | 0.45 | 0.54 | 0.64 | 0.73 | 0.80 | 0.85 | 0.90 | 0.94 | 0.97 | 0.98 | 0.99 | 1.00 | 1.00 | 1.00 | 1.00 | 1.00 | 1.00 | 1.00 | 1.00 | 1.00 | 1.00 | 1.00 | 1.00 | 1.00 |
| 140 | 0.07 | 0.11 | 0.17 | 0.26 | 0.38 | 0.51 | 0.62 | 0.72 | 0.81 | 0.88 | 0.93 | 0.96 | 0.98 | 1.00 | 1.00 | 1.00 | 1.00 | 1.00 | 1.00 | 1.00 | 1.00 | 1.00 | 1.00 | 1.00 | 1.00 | 1.00 | 1.00 | 1.00 | 1.00 | 1.00 |
| 160 | 0.10 | 0.16 | 0.26 | 0.38 | 0.51 | 0.66 | 0.78 | 0.87 | 0.94 | 0.98 | 0.99 | 1.00 | 1.00 | 1.00 | 1.00 | 1.00 | 1.00 | 1.00 | 1.00 | 1.00 | 1.00 | 1.00 | 1.00 | 1.00 | 1.00 | 1.00 | 1.00 | 1.00 | 1.00 | 1.00 |
| 180 | 0.10 | 0.20 | 0.33 | 0.49 | 0.65 | 0.80 | 0.90 | 0.96 | 0.99 | 1.00 | 1.00 | 1.00 | 1.00 | 1.00 | 1.00 | 1.00 | 1.00 | 1.00 | 1.00 | 1.00 | 1.00 | 1.00 | 1.00 | 1.00 | 1.00 | 1.00 | 1.00 | 1.00 | 1.00 | 1.00 |
| 200 | 0.11 | 0.21 | 0.36 | 0.58 | 0.77 | 0.90 | 0.96 | 0.99 | 1.00 | 1.00 | 1.00 | 1.00 | 1.00 | 1.00 | 1.00 | 1.00 | 1.00 | 1.00 | 1.00 | 1.00 | 1.00 | 1.00 | 1.00 | 1.00 | 1.00 | 1.00 | 1.00 | 1.00 | 1.00 | 1.00 |
| 220 | 0.12 | 0.25 | 0.47 | 0.69 | 0.85 | 0.95 | 0.98 | 1.00 | 1.00 | 1.00 | 1.00 | 1.00 | 1.00 | 1.00 | 1.00 | 1.00 | 1.00 | 1.00 | 1.00 | 1.00 | 1.00 | 1.00 | 1.00 | 1.00 | 1.00 | 1.00 | 1.00 | 1.00 | 1.00 | 1.00 |
| 240 | 0.11 | 0.29 | 0.54 | 0.78 | 0.93 | 0.99 | 1.00 | 1.00 | 1.00 | 1.00 | 1.00 | 1.00 | 1.00 | 1.00 | 1.00 | 1.00 | 1.00 | 1.00 | 1.00 | 1.00 | 1.00 | 1.00 | 1.00 | 1.00 | 1.00 | 1.00 | 1.00 | 1.00 | 1.00 | 1.00 |
| 260 | 0.14 | 0.38 | 0.67 | 0.88 | 0.98 | 1.00 | 1.00 | 1.00 | 1.00 | 1.00 | 1.00 | 1.00 | 1.00 | 1.00 | 1.00 | 1.00 | 1.00 | 1.00 | 1.00 | 1.00 | 1.00 | 1.00 | 1.00 | 1.00 | 1.00 | 1.00 | 1.00 | 1.00 | 1.00 | 1.00 |
| 280 | 0.16 | 0.43 | 0.73 | 0.93 | 0.99 | 1.00 | 1.00 | 1.00 | 1.00 | 1.00 | 1.00 | 1.00 | 1.00 | 1.00 | 1.00 | 1.00 | 1.00 | 1.00 | 1.00 | 1.00 | 1.00 | 1.00 | 1.00 | 1.00 | 1.00 | 1.00 | 1.00 | 1.00 | 1.00 | 1.00 |
| 300 | 0.16 | 0.48 | 0.79 | 0.96 | 1.00 | 1.00 | 1.00 | 1.00 | 1.00 | 1.00 | 1.00 | 1.00 | 1.00 | 1.00 | 1.00 | 1.00 | 1.00 | 1.00 | 1.00 | 1.00 | 1.00 | 1.00 | 1.00 | 1.00 | 1.00 | 1.00 | 1.00 | 1.00 | 1.00 | 1.00 |
| 320 | 0.18 | 0.55 | 0.88 | 0.99 | 1.00 | 1.00 | 1.00 | 1.00 | 1.00 | 1.00 | 1.00 | 1.00 | 1.00 | 1.00 | 1.00 | 1.00 | 1.00 | 1.00 | 1.00 | 1.00 | 1.00 | 1.00 | 1.00 | 1.00 | 1.00 | 1.00 | 1.00 | 1.00 | 1.00 | 1.00 |
| 340 | 0.23 | 0.64 | 0.93 | 1.00 | 1.00 | 1.00 | 1.00 | 1.00 | 1.00 | 1.00 | 1.00 | 1.00 | 1.00 | 1.00 | 1.00 | 1.00 | 1.00 | 1.00 | 1.00 | 1.00 | 1.00 | 1.00 | 1.00 | 1.00 | 1.00 | 1.00 | 1.00 | 1.00 | 1.00 | 1.00 |
| 360 | 0.26 | 0.73 | 0.96 | 1.00 | 1.00 | 1.00 | 1.00 | 1.00 | 1.00 | 1.00 | 1.00 | 1.00 | 1.00 | 1.00 | 1.00 | 1.00 | 1.00 | 1.00 | 1.00 | 1.00 | 1.00 | 1.00 | 1.00 | 1.00 | 1.00 | 1.00 | 1.00 | 1.00 | 1.00 | 1.00 |
| 380 | 0.30 | 0.79 | 0.98 | 1.00 | 1.00 | 1.00 | 1.00 | 1.00 | 1.00 | 1.00 | 1.00 | 1.00 | 1.00 | 1.00 | 1.00 | 1.00 | 1.00 | 1.00 | 1.00 | 1.00 | 1.00 | 1.00 | 1.00 | 1.00 | 1.00 | 1.00 | 1.00 | 1.00 | 1.00 | 1.00 |
| 400 | 0.33 | 0.83 | 0.99 | 1.00 | 1.00 | 1.00 | 1.00 | 1.00 | 1.00 | 1.00 | 1.00 | 1.00 | 1.00 | 1.00 | 1.00 | 1.00 | 1.00 | 1.00 | 1.00 | 1.00 | 1.00 | 1.00 | 1.00 | 1.00 | 1.00 | 1.00 | 1.00 | 1.00 | 1.00 | 1.00 |

Note: In the simulations it was assumed that there was no underlying trend or covariates which needed to be adjusted for. The intervention was assumed to have occurred at the midpoint of the time series.

Table 8: Power to detect a change in trend as a function of time and size of the change in trend, with the presence of AR= 0.9 autocorrelation and standard deviation of the white noise of 2.

|  | Effect size for the change in trend | | | | | | | | | | | | | | | | | | | | | | | | | | | | | |
| --- | --- | --- | --- | --- | --- | --- | --- | --- | --- | --- | --- | --- | --- | --- | --- | --- | --- | --- | --- | --- | --- | --- | --- | --- | --- | --- | --- | --- | --- | --- |
| Time | 0.01 | 0.02 | 0.03 | 0.04 | 0.05 | 0.06 | 0.07 | 0.08 | 0.09 | 0.1 | 0.11 | 0.12 | 0.13 | 0.14 | 0.15 | 0.16 | 0.17 | 0.18 | 0.19 | 0.2 | 0.21 | 0.22 | 0.23 | 0.24 | 0.25 | 0.26 | 0.27 | 0.28 | 0.29 | 0.3 |
| 40 | 0.38 | 0.39 | 0.39 | 0.39 | 0.39 | 0.39 | 0.40 | 0.40 | 0.41 | 0.41 | 0.41 | 0.41 | 0.41 | 0.41 | 0.41 | 0.41 | 0.42 | 0.42 | 0.42 | 0.42 | 0.41 | 0.41 | 0.42 | 0.42 | 0.42 | 0.42 | 0.42 | 0.43 | 0.44 | 0.44 |
| 60 | 0.28 | 0.28 | 0.29 | 0.29 | 0.29 | 0.29 | 0.29 | 0.29 | 0.30 | 0.30 | 0.30 | 0.30 | 0.30 | 0.31 | 0.31 | 0.32 | 0.32 | 0.33 | 0.34 | 0.35 | 0.36 | 0.36 | 0.37 | 0.38 | 0.39 | 0.40 | 0.40 | 0.40 | 0.41 | 0.42 |
| 80 | 0.27 | 0.27 | 0.28 | 0.28 | 0.28 | 0.28 | 0.29 | 0.30 | 0.31 | 0.31 | 0.31 | 0.33 | 0.34 | 0.34 | 0.36 | 0.37 | 0.37 | 0.39 | 0.40 | 0.40 | 0.41 | 0.42 | 0.43 | 0.44 | 0.45 | 0.46 | 0.48 | 0.50 | 0.50 | 0.52 |
| 100 | 0.22 | 0.22 | 0.23 | 0.23 | 0.24 | 0.24 | 0.25 | 0.26 | 0.28 | 0.29 | 0.30 | 0.31 | 0.32 | 0.33 | 0.34 | 0.36 | 0.38 | 0.39 | 0.41 | 0.42 | 0.43 | 0.46 | 0.47 | 0.49 | 0.51 | 0.52 | 0.54 | 0.56 | 0.58 | 0.60 |
| 120 | 0.19 | 0.19 | 0.19 | 0.20 | 0.22 | 0.23 | 0.25 | 0.27 | 0.27 | 0.29 | 0.30 | 0.32 | 0.33 | 0.34 | 0.37 | 0.39 | 0.41 | 0.44 | 0.45 | 0.47 | 0.49 | 0.51 | 0.53 | 0.56 | 0.58 | 0.60 | 0.63 | 0.65 | 0.67 | 0.69 |
| 140 | 0.18 | 0.18 | 0.19 | 0.20 | 0.20 | 0.21 | 0.22 | 0.24 | 0.26 | 0.28 | 0.31 | 0.33 | 0.36 | 0.39 | 0.42 | 0.44 | 0.47 | 0.49 | 0.52 | 0.54 | 0.57 | 0.60 | 0.62 | 0.66 | 0.69 | 0.71 | 0.73 | 0.76 | 0.78 | 0.81 |
| 160 | 0.17 | 0.18 | 0.19 | 0.20 | 0.21 | 0.23 | 0.25 | 0.27 | 0.30 | 0.33 | 0.35 | 0.38 | 0.40 | 0.43 | 0.47 | 0.50 | 0.54 | 0.57 | 0.60 | 0.63 | 0.67 | 0.70 | 0.72 | 0.74 | 0.76 | 0.79 | 0.82 | 0.84 | 0.86 | 0.88 |
| 180 | 0.18 | 0.19 | 0.20 | 0.21 | 0.23 | 0.27 | 0.30 | 0.32 | 0.35 | 0.38 | 0.42 | 0.46 | 0.49 | 0.53 | 0.56 | 0.59 | 0.62 | 0.66 | 0.69 | 0.72 | 0.76 | 0.79 | 0.82 | 0.85 | 0.88 | 0.90 | 0.91 | 0.92 | 0.94 | 0.95 |
| 200 | 0.16 | 0.17 | 0.17 | 0.18 | 0.21 | 0.25 | 0.27 | 0.30 | 0.34 | 0.38 | 0.41 | 0.46 | 0.50 | 0.56 | 0.60 | 0.65 | 0.69 | 0.72 | 0.76 | 0.80 | 0.82 | 0.84 | 0.88 | 0.90 | 0.92 | 0.94 | 0.95 | 0.96 | 0.97 | 0.98 |
| 220 | 0.16 | 0.16 | 0.17 | 0.19 | 0.22 | 0.25 | 0.28 | 0.31 | 0.36 | 0.40 | 0.45 | 0.50 | 0.56 | 0.61 | 0.67 | 0.72 | 0.76 | 0.80 | 0.83 | 0.86 | 0.88 | 0.91 | 0.92 | 0.94 | 0.96 | 0.96 | 0.97 | 0.98 | 0.99 | 0.99 |
| 240 | 0.14 | 0.15 | 0.16 | 0.18 | 0.22 | 0.27 | 0.33 | 0.38 | 0.42 | 0.48 | 0.52 | 0.59 | 0.64 | 0.69 | 0.74 | 0.78 | 0.83 | 0.87 | 0.90 | 0.92 | 0.94 | 0.96 | 0.97 | 0.98 | 0.99 | 0.99 | 0.99 | 0.99 | 0.99 | 1.00 |
| 260 | 0.14 | 0.16 | 0.18 | 0.22 | 0.26 | 0.32 | 0.38 | 0.45 | 0.51 | 0.56 | 0.63 | 0.69 | 0.74 | 0.80 | 0.83 | 0.86 | 0.89 | 0.93 | 0.94 | 0.96 | 0.97 | 0.98 | 0.99 | 1.00 | 1.00 | 1.00 | 1.00 | 1.00 | 1.00 | 1.00 |
| 280 | 0.14 | 0.16 | 0.18 | 0.23 | 0.28 | 0.34 | 0.42 | 0.49 | 0.55 | 0.63 | 0.69 | 0.75 | 0.81 | 0.85 | 0.88 | 0.90 | 0.93 | 0.96 | 0.98 | 0.98 | 0.99 | 0.99 | 1.00 | 1.00 | 1.00 | 1.00 | 1.00 | 1.00 | 1.00 | 1.00 |
| 300 | 0.14 | 0.15 | 0.17 | 0.23 | 0.29 | 0.35 | 0.43 | 0.50 | 0.58 | 0.67 | 0.74 | 0.81 | 0.86 | 0.90 | 0.93 | 0.96 | 0.97 | 0.97 | 0.98 | 0.99 | 0.99 | 1.00 | 1.00 | 1.00 | 1.00 | 1.00 | 1.00 | 1.00 | 1.00 | 1.00 |
| 320 | 0.11 | 0.14 | 0.19 | 0.26 | 0.33 | 0.40 | 0.47 | 0.57 | 0.66 | 0.72 | 0.79 | 0.85 | 0.90 | 0.93 | 0.96 | 0.97 | 0.98 | 0.99 | 1.00 | 1.00 | 1.00 | 1.00 | 1.00 | 1.00 | 1.00 | 1.00 | 1.00 | 1.00 | 1.00 | 1.00 |
| 340 | 0.11 | 0.15 | 0.21 | 0.26 | 0.35 | 0.43 | 0.52 | 0.62 | 0.71 | 0.78 | 0.85 | 0.90 | 0.93 | 0.95 | 0.97 | 0.98 | 0.99 | 0.99 | 1.00 | 1.00 | 1.00 | 1.00 | 1.00 | 1.00 | 1.00 | 1.00 | 1.00 | 1.00 | 1.00 | 1.00 |
| 360 | 0.12 | 0.15 | 0.20 | 0.27 | 0.37 | 0.45 | 0.56 | 0.64 | 0.73 | 0.82 | 0.88 | 0.93 | 0.95 | 0.98 | 0.99 | 0.99 | 1.00 | 1.00 | 1.00 | 1.00 | 1.00 | 1.00 | 1.00 | 1.00 | 1.00 | 1.00 | 1.00 | 1.00 | 1.00 | 1.00 |
| 380 | 0.13 | 0.18 | 0.26 | 0.35 | 0.45 | 0.56 | 0.66 | 0.74 | 0.82 | 0.88 | 0.94 | 0.97 | 0.98 | 0.99 | 1.00 | 1.00 | 1.00 | 1.00 | 1.00 | 1.00 | 1.00 | 1.00 | 1.00 | 1.00 | 1.00 | 1.00 | 1.00 | 1.00 | 1.00 | 1.00 |
| 400 | 0.12 | 0.17 | 0.25 | 0.36 | 0.46 | 0.57 | 0.68 | 0.77 | 0.84 | 0.90 | 0.94 | 0.97 | 0.99 | 1.00 | 1.00 | 1.00 | 1.00 | 1.00 | 1.00 | 1.00 | 1.00 | 1.00 | 1.00 | 1.00 | 1.00 | 1.00 | 1.00 | 1.00 | 1.00 | 1.00 |

Note: In the simulations it was assumed that there was no underlying trend or covariates which needed to be adjusted for. The intervention was assumed to have occurred at the midpoint of the time series.

Table 9: Power to detect a change in trend as a function of time and size of the change in trend, with the presence of AR= 0.1 autocorrelation and standard deviation of the white noise of 3.

|  | Effect size for the change in trend | | | | | | | | | | | | | | | | | | | | | | | | | | | | | |
| --- | --- | --- | --- | --- | --- | --- | --- | --- | --- | --- | --- | --- | --- | --- | --- | --- | --- | --- | --- | --- | --- | --- | --- | --- | --- | --- | --- | --- | --- | --- |
| Time | 0.01 | 0.02 | 0.03 | 0.04 | 0.05 | 0.06 | 0.07 | 0.08 | 0.09 | 0.1 | 0.11 | 0.12 | 0.13 | 0.14 | 0.15 | 0.16 | 0.17 | 0.18 | 0.19 | 0.2 | 0.21 | 0.22 | 0.23 | 0.24 | 0.25 | 0.26 | 0.27 | 0.28 | 0.29 | 0.3 |
| 40 | 0.10 | 0.10 | 0.09 | 0.09 | 0.10 | 0.11 | 0.11 | 0.13 | 0.13 | 0.14 | 0.15 | 0.16 | 0.17 | 0.19 | 0.20 | 0.22 | 0.23 | 0.25 | 0.26 | 0.27 | 0.28 | 0.30 | 0.32 | 0.33 | 0.35 | 0.37 | 0.38 | 0.41 | 0.43 | 0.45 |
| 60 | 0.08 | 0.08 | 0.09 | 0.10 | 0.11 | 0.13 | 0.15 | 0.18 | 0.20 | 0.22 | 0.25 | 0.28 | 0.30 | 0.33 | 0.38 | 0.42 | 0.46 | 0.51 | 0.55 | 0.59 | 0.63 | 0.68 | 0.71 | 0.74 | 0.77 | 0.80 | 0.82 | 0.86 | 0.88 | 0.90 |
| 80 | 0.09 | 0.09 | 0.11 | 0.14 | 0.17 | 0.20 | 0.25 | 0.29 | 0.34 | 0.39 | 0.45 | 0.53 | 0.58 | 0.64 | 0.69 | 0.75 | 0.78 | 0.83 | 0.87 | 0.90 | 0.93 | 0.94 | 0.95 | 0.97 | 0.98 | 0.99 | 0.99 | 1.00 | 1.00 | 1.00 |
| 100 | 0.07 | 0.08 | 0.11 | 0.16 | 0.22 | 0.31 | 0.39 | 0.47 | 0.55 | 0.63 | 0.70 | 0.76 | 0.82 | 0.88 | 0.92 | 0.95 | 0.97 | 0.98 | 0.99 | 1.00 | 1.00 | 1.00 | 1.00 | 1.00 | 1.00 | 1.00 | 1.00 | 1.00 | 1.00 | 1.00 |
| 120 | 0.07 | 0.10 | 0.14 | 0.22 | 0.33 | 0.43 | 0.53 | 0.65 | 0.74 | 0.83 | 0.89 | 0.94 | 0.97 | 0.98 | 0.99 | 1.00 | 1.00 | 1.00 | 1.00 | 1.00 | 1.00 | 1.00 | 1.00 | 1.00 | 1.00 | 1.00 | 1.00 | 1.00 | 1.00 | 1.00 |
| 140 | 0.08 | 0.15 | 0.24 | 0.35 | 0.49 | 0.63 | 0.74 | 0.85 | 0.91 | 0.96 | 0.98 | 0.99 | 1.00 | 1.00 | 1.00 | 1.00 | 1.00 | 1.00 | 1.00 | 1.00 | 1.00 | 1.00 | 1.00 | 1.00 | 1.00 | 1.00 | 1.00 | 1.00 | 1.00 | 1.00 |
| 160 | 0.09 | 0.17 | 0.29 | 0.46 | 0.62 | 0.76 | 0.89 | 0.95 | 0.98 | 0.99 | 1.00 | 1.00 | 1.00 | 1.00 | 1.00 | 1.00 | 1.00 | 1.00 | 1.00 | 1.00 | 1.00 | 1.00 | 1.00 | 1.00 | 1.00 | 1.00 | 1.00 | 1.00 | 1.00 | 1.00 |
| 180 | 0.08 | 0.19 | 0.34 | 0.58 | 0.77 | 0.89 | 0.96 | 0.99 | 1.00 | 1.00 | 1.00 | 1.00 | 1.00 | 1.00 | 1.00 | 1.00 | 1.00 | 1.00 | 1.00 | 1.00 | 1.00 | 1.00 | 1.00 | 1.00 | 1.00 | 1.00 | 1.00 | 1.00 | 1.00 | 1.00 |
| 200 | 0.10 | 0.25 | 0.48 | 0.71 | 0.87 | 0.95 | 0.99 | 1.00 | 1.00 | 1.00 | 1.00 | 1.00 | 1.00 | 1.00 | 1.00 | 1.00 | 1.00 | 1.00 | 1.00 | 1.00 | 1.00 | 1.00 | 1.00 | 1.00 | 1.00 | 1.00 | 1.00 | 1.00 | 1.00 | 1.00 |
| 220 | 0.13 | 0.32 | 0.59 | 0.83 | 0.95 | 0.99 | 1.00 | 1.00 | 1.00 | 1.00 | 1.00 | 1.00 | 1.00 | 1.00 | 1.00 | 1.00 | 1.00 | 1.00 | 1.00 | 1.00 | 1.00 | 1.00 | 1.00 | 1.00 | 1.00 | 1.00 | 1.00 | 1.00 | 1.00 | 1.00 |
| 240 | 0.14 | 0.38 | 0.67 | 0.90 | 0.98 | 1.00 | 1.00 | 1.00 | 1.00 | 1.00 | 1.00 | 1.00 | 1.00 | 1.00 | 1.00 | 1.00 | 1.00 | 1.00 | 1.00 | 1.00 | 1.00 | 1.00 | 1.00 | 1.00 | 1.00 | 1.00 | 1.00 | 1.00 | 1.00 | 1.00 |
| 260 | 0.16 | 0.48 | 0.81 | 0.96 | 1.00 | 1.00 | 1.00 | 1.00 | 1.00 | 1.00 | 1.00 | 1.00 | 1.00 | 1.00 | 1.00 | 1.00 | 1.00 | 1.00 | 1.00 | 1.00 | 1.00 | 1.00 | 1.00 | 1.00 | 1.00 | 1.00 | 1.00 | 1.00 | 1.00 | 1.00 |
| 280 | 0.18 | 0.52 | 0.86 | 0.98 | 1.00 | 1.00 | 1.00 | 1.00 | 1.00 | 1.00 | 1.00 | 1.00 | 1.00 | 1.00 | 1.00 | 1.00 | 1.00 | 1.00 | 1.00 | 1.00 | 1.00 | 1.00 | 1.00 | 1.00 | 1.00 | 1.00 | 1.00 | 1.00 | 1.00 | 1.00 |
| 300 | 0.23 | 0.63 | 0.92 | 0.99 | 1.00 | 1.00 | 1.00 | 1.00 | 1.00 | 1.00 | 1.00 | 1.00 | 1.00 | 1.00 | 1.00 | 1.00 | 1.00 | 1.00 | 1.00 | 1.00 | 1.00 | 1.00 | 1.00 | 1.00 | 1.00 | 1.00 | 1.00 | 1.00 | 1.00 | 1.00 |
| 320 | 0.26 | 0.74 | 0.98 | 1.00 | 1.00 | 1.00 | 1.00 | 1.00 | 1.00 | 1.00 | 1.00 | 1.00 | 1.00 | 1.00 | 1.00 | 1.00 | 1.00 | 1.00 | 1.00 | 1.00 | 1.00 | 1.00 | 1.00 | 1.00 | 1.00 | 1.00 | 1.00 | 1.00 | 1.00 | 1.00 |
| 340 | 0.27 | 0.78 | 0.98 | 1.00 | 1.00 | 1.00 | 1.00 | 1.00 | 1.00 | 1.00 | 1.00 | 1.00 | 1.00 | 1.00 | 1.00 | 1.00 | 1.00 | 1.00 | 1.00 | 1.00 | 1.00 | 1.00 | 1.00 | 1.00 | 1.00 | 1.00 | 1.00 | 1.00 | 1.00 | 1.00 |
| 360 | 0.35 | 0.85 | 1.00 | 1.00 | 1.00 | 1.00 | 1.00 | 1.00 | 1.00 | 1.00 | 1.00 | 1.00 | 1.00 | 1.00 | 1.00 | 1.00 | 1.00 | 1.00 | 1.00 | 1.00 | 1.00 | 1.00 | 1.00 | 1.00 | 1.00 | 1.00 | 1.00 | 1.00 | 1.00 | 1.00 |
| 380 | 0.40 | 0.90 | 1.00 | 1.00 | 1.00 | 1.00 | 1.00 | 1.00 | 1.00 | 1.00 | 1.00 | 1.00 | 1.00 | 1.00 | 1.00 | 1.00 | 1.00 | 1.00 | 1.00 | 1.00 | 1.00 | 1.00 | 1.00 | 1.00 | 1.00 | 1.00 | 1.00 | 1.00 | 1.00 | 1.00 |
| 400 | 0.41 | 0.94 | 1.00 | 1.00 | 1.00 | 1.00 | 1.00 | 1.00 | 1.00 | 1.00 | 1.00 | 1.00 | 1.00 | 1.00 | 1.00 | 1.00 | 1.00 | 1.00 | 1.00 | 1.00 | 1.00 | 1.00 | 1.00 | 1.00 | 1.00 | 1.00 | 1.00 | 1.00 | 1.00 | 1.00 |

Note: In the simulations it was assumed that there was no underlying trend or covariates which needed to be adjusted for. The intervention was assumed to have occurred at the midpoint of the time series.

Table 10: Power to detect a change in trend as a function of time and size of the change in trend, with the presence of AR= 0.5 autocorrelation and standard deviation of the white noise of 3.

|  | Effect size for the change in trend | | | | | | | | | | | | | | | | | | | | | | | | | | | | | |
| --- | --- | --- | --- | --- | --- | --- | --- | --- | --- | --- | --- | --- | --- | --- | --- | --- | --- | --- | --- | --- | --- | --- | --- | --- | --- | --- | --- | --- | --- | --- |
| Time | 0.01 | 0.02 | 0.03 | 0.04 | 0.05 | 0.06 | 0.07 | 0.08 | 0.09 | 0.1 | 0.11 | 0.12 | 0.13 | 0.14 | 0.15 | 0.16 | 0.17 | 0.18 | 0.19 | 0.2 | 0.21 | 0.22 | 0.23 | 0.24 | 0.25 | 0.26 | 0.27 | 0.28 | 0.29 | 0.3 |
| 40 |  |  |  |  |  |  |  |  |  |  |  |  |  |  |  |  |  |  |  |  |  |  |  |  |  |  |  |  |  |  |
| 60 | 0.13 | 0.14 | 0.14 | 0.14 | 0.14 | 0.15 | 0.15 | 0.16 | 0.16 | 0.16 | 0.16 | 0.17 | 0.18 | 0.19 | 0.20 | 0.20 | 0.21 | 0.22 | 0.22 | 0.23 | 0.24 | 0.25 | 0.26 | 0.27 | 0.28 | 0.29 | 0.30 | 0.31 | 0.32 | 0.33 |
| 80 | 0.13 | 0.13 | 0.13 | 0.13 | 0.13 | 0.13 | 0.13 | 0.14 | 0.14 | 0.15 | 0.16 | 0.18 | 0.19 | 0.21 | 0.22 | 0.23 | 0.25 | 0.27 | 0.29 | 0.31 | 0.32 | 0.34 | 0.36 | 0.39 | 0.42 | 0.43 | 0.45 | 0.48 | 0.50 | 0.52 |
| 100 | 0.08 | 0.09 | 0.10 | 0.11 | 0.12 | 0.13 | 0.15 | 0.17 | 0.20 | 0.22 | 0.23 | 0.26 | 0.29 | 0.32 | 0.35 | 0.38 | 0.40 | 0.45 | 0.48 | 0.52 | 0.56 | 0.59 | 0.63 | 0.66 | 0.69 | 0.72 | 0.75 | 0.77 | 0.80 | 0.83 |
| 120 | 0.09 | 0.10 | 0.10 | 0.12 | 0.14 | 0.17 | 0.21 | 0.24 | 0.28 | 0.32 | 0.36 | 0.41 | 0.44 | 0.50 | 0.55 | 0.59 | 0.64 | 0.68 | 0.71 | 0.75 | 0.79 | 0.83 | 0.86 | 0.88 | 0.90 | 0.92 | 0.93 | 0.95 | 0.96 | 0.97 |
| 140 | 0.08 | 0.10 | 0.12 | 0.14 | 0.17 | 0.21 | 0.25 | 0.30 | 0.37 | 0.42 | 0.48 | 0.54 | 0.60 | 0.67 | 0.73 | 0.77 | 0.82 | 0.85 | 0.89 | 0.91 | 0.94 | 0.96 | 0.97 | 0.98 | 0.99 | 0.99 | 1.00 | 1.00 | 1.00 | 1.00 |
| 160 | 0.06 | 0.09 | 0.11 | 0.15 | 0.19 | 0.26 | 0.34 | 0.43 | 0.51 | 0.59 | 0.65 | 0.72 | 0.78 | 0.84 | 0.88 | 0.92 | 0.94 | 0.96 | 0.98 | 0.99 | 1.00 | 1.00 | 1.00 | 1.00 | 1.00 | 1.00 | 1.00 | 1.00 | 1.00 | 1.00 |
| 180 | 0.09 | 0.12 | 0.16 | 0.22 | 0.29 | 0.38 | 0.45 | 0.55 | 0.66 | 0.75 | 0.82 | 0.87 | 0.93 | 0.96 | 0.98 | 0.99 | 0.99 | 1.00 | 1.00 | 1.00 | 1.00 | 1.00 | 1.00 | 1.00 | 1.00 | 1.00 | 1.00 | 1.00 | 1.00 | 1.00 |
| 200 | 0.08 | 0.12 | 0.20 | 0.28 | 0.38 | 0.49 | 0.60 | 0.70 | 0.80 | 0.88 | 0.93 | 0.96 | 0.98 | 0.99 | 1.00 | 1.00 | 1.00 | 1.00 | 1.00 | 1.00 | 1.00 | 1.00 | 1.00 | 1.00 | 1.00 | 1.00 | 1.00 | 1.00 | 1.00 | 1.00 |
| 220 | 0.08 | 0.13 | 0.21 | 0.30 | 0.43 | 0.58 | 0.69 | 0.81 | 0.90 | 0.95 | 0.98 | 0.99 | 1.00 | 1.00 | 1.00 | 1.00 | 1.00 | 1.00 | 1.00 | 1.00 | 1.00 | 1.00 | 1.00 | 1.00 | 1.00 | 1.00 | 1.00 | 1.00 | 1.00 | 1.00 |
| 240 | 0.11 | 0.15 | 0.25 | 0.40 | 0.54 | 0.69 | 0.81 | 0.91 | 0.95 | 0.98 | 0.99 | 1.00 | 1.00 | 1.00 | 1.00 | 1.00 | 1.00 | 1.00 | 1.00 | 1.00 | 1.00 | 1.00 | 1.00 | 1.00 | 1.00 | 1.00 | 1.00 | 1.00 | 1.00 | 1.00 |
| 260 | 0.09 | 0.16 | 0.29 | 0.45 | 0.64 | 0.78 | 0.90 | 0.96 | 0.99 | 1.00 | 1.00 | 1.00 | 1.00 | 1.00 | 1.00 | 1.00 | 1.00 | 1.00 | 1.00 | 1.00 | 1.00 | 1.00 | 1.00 | 1.00 | 1.00 | 1.00 | 1.00 | 1.00 | 1.00 | 1.00 |
| 280 | 0.11 | 0.22 | 0.38 | 0.57 | 0.75 | 0.88 | 0.96 | 0.99 | 1.00 | 1.00 | 1.00 | 1.00 | 1.00 | 1.00 | 1.00 | 1.00 | 1.00 | 1.00 | 1.00 | 1.00 | 1.00 | 1.00 | 1.00 | 1.00 | 1.00 | 1.00 | 1.00 | 1.00 | 1.00 | 1.00 |
| 300 | 0.11 | 0.23 | 0.44 | 0.65 | 0.81 | 0.93 | 0.98 | 1.00 | 1.00 | 1.00 | 1.00 | 1.00 | 1.00 | 1.00 | 1.00 | 1.00 | 1.00 | 1.00 | 1.00 | 1.00 | 1.00 | 1.00 | 1.00 | 1.00 | 1.00 | 1.00 | 1.00 | 1.00 | 1.00 | 1.00 |
| 320 | 0.11 | 0.24 | 0.48 | 0.71 | 0.87 | 0.96 | 0.99 | 1.00 | 1.00 | 1.00 | 1.00 | 1.00 | 1.00 | 1.00 | 1.00 | 1.00 | 1.00 | 1.00 | 1.00 | 1.00 | 1.00 | 1.00 | 1.00 | 1.00 | 1.00 | 1.00 | 1.00 | 1.00 | 1.00 | 1.00 |
| 340 | 0.11 | 0.30 | 0.55 | 0.80 | 0.94 | 0.99 | 1.00 | 1.00 | 1.00 | 1.00 | 1.00 | 1.00 | 1.00 | 1.00 | 1.00 | 1.00 | 1.00 | 1.00 | 1.00 | 1.00 | 1.00 | 1.00 | 1.00 | 1.00 | 1.00 | 1.00 | 1.00 | 1.00 | 1.00 | 1.00 |
| 360 | 0.13 | 0.34 | 0.64 | 0.85 | 0.96 | 1.00 | 1.00 | 1.00 | 1.00 | 1.00 | 1.00 | 1.00 | 1.00 | 1.00 | 1.00 | 1.00 | 1.00 | 1.00 | 1.00 | 1.00 | 1.00 | 1.00 | 1.00 | 1.00 | 1.00 | 1.00 | 1.00 | 1.00 | 1.00 | 1.00 |
| 380 | 0.14 | 0.40 | 0.73 | 0.92 | 0.99 | 1.00 | 1.00 | 1.00 | 1.00 | 1.00 | 1.00 | 1.00 | 1.00 | 1.00 | 1.00 | 1.00 | 1.00 | 1.00 | 1.00 | 1.00 | 1.00 | 1.00 | 1.00 | 1.00 | 1.00 | 1.00 | 1.00 | 1.00 | 1.00 | 1.00 |
| 400 | 0.15 | 0.47 | 0.79 | 0.95 | 1.00 | 1.00 | 1.00 | 1.00 | 1.00 | 1.00 | 1.00 | 1.00 | 1.00 | 1.00 | 1.00 | 1.00 | 1.00 | 1.00 | 1.00 | 1.00 | 1.00 | 1.00 | 1.00 | 1.00 | 1.00 | 1.00 | 1.00 | 1.00 | 1.00 | 1.00 |

Note: In the simulations it was assumed that there was no underlying trend or covariates which needed to be adjusted for. The intervention was assumed to have occurred at the midpoint of the time series.

Table 11: Power to detect a change in trend as a function of time and size of the change in trend, with the presence of AR= 0.9 autocorrelation and standard deviation of the white noise of 3.

|  | Effect size for the change in trend | | | | | | | | | | | | | | | | | | | | | | | | | | | | | |
| --- | --- | --- | --- | --- | --- | --- | --- | --- | --- | --- | --- | --- | --- | --- | --- | --- | --- | --- | --- | --- | --- | --- | --- | --- | --- | --- | --- | --- | --- | --- |
| Time | 0.01 | 0.02 | 0.03 | 0.04 | 0.05 | 0.06 | 0.07 | 0.08 | 0.09 | 0.1 | 0.11 | 0.12 | 0.13 | 0.14 | 0.15 | 0.16 | 0.17 | 0.18 | 0.19 | 0.2 | 0.21 | 0.22 | 0.23 | 0.24 | 0.25 | 0.26 | 0.27 | 0.28 | 0.29 | 0.3 |
| 40 | 0.39 | 0.38 | 0.39 | 0.39 | 0.39 | 0.40 | 0.39 | 0.39 | 0.39 | 0.39 | 0.40 | 0.40 | 0.40 | 0.40 | 0.40 | 0.40 | 0.40 | 0.40 | 0.41 | 0.40 | 0.41 | 0.41 | 0.41 | 0.41 | 0.41 | 0.41 | 0.42 | 0.42 | 0.41 | 0.41 |
| 60 | 0.29 | 0.29 | 0.29 | 0.29 | 0.29 | 0.29 | 0.29 | 0.29 | 0.29 | 0.29 | 0.29 | 0.29 | 0.29 | 0.29 | 0.30 | 0.30 | 0.30 | 0.30 | 0.31 | 0.31 | 0.31 | 0.31 | 0.32 | 0.32 | 0.32 | 0.33 | 0.33 | 0.33 | 0.34 | 0.34 |
| 80 | 0.26 | 0.27 | 0.27 | 0.27 | 0.27 | 0.27 | 0.28 | 0.28 | 0.28 | 0.28 | 0.29 | 0.29 | 0.30 | 0.30 | 0.31 | 0.31 | 0.32 | 0.33 | 0.33 | 0.33 | 0.34 | 0.35 | 0.36 | 0.36 | 0.37 | 0.38 | 0.38 | 0.39 | 0.40 | 0.41 |
| 100 | 0.22 | 0.22 | 0.22 | 0.22 | 0.23 | 0.23 | 0.24 | 0.25 | 0.24 | 0.25 | 0.25 | 0.25 | 0.26 | 0.28 | 0.28 | 0.29 | 0.30 | 0.31 | 0.31 | 0.32 | 0.33 | 0.34 | 0.35 | 0.36 | 0.37 | 0.38 | 0.39 | 0.40 | 0.41 | 0.42 |
| 120 | 0.19 | 0.19 | 0.19 | 0.19 | 0.20 | 0.20 | 0.21 | 0.22 | 0.23 | 0.24 | 0.25 | 0.26 | 0.27 | 0.27 | 0.28 | 0.29 | 0.30 | 0.31 | 0.32 | 0.33 | 0.34 | 0.36 | 0.37 | 0.38 | 0.40 | 0.42 | 0.43 | 0.45 | 0.46 | 0.47 |
| 140 | 0.17 | 0.18 | 0.18 | 0.18 | 0.19 | 0.20 | 0.20 | 0.20 | 0.21 | 0.22 | 0.23 | 0.24 | 0.25 | 0.27 | 0.28 | 0.30 | 0.31 | 0.33 | 0.34 | 0.37 | 0.39 | 0.40 | 0.42 | 0.44 | 0.46 | 0.48 | 0.49 | 0.51 | 0.52 | 0.54 |
| 160 | 0.17 | 0.17 | 0.18 | 0.18 | 0.19 | 0.20 | 0.21 | 0.22 | 0.23 | 0.25 | 0.26 | 0.27 | 0.30 | 0.32 | 0.33 | 0.34 | 0.36 | 0.37 | 0.39 | 0.41 | 0.42 | 0.46 | 0.48 | 0.50 | 0.52 | 0.55 | 0.57 | 0.59 | 0.62 | 0.63 |
| 180 | 0.18 | 0.18 | 0.19 | 0.19 | 0.20 | 0.21 | 0.23 | 0.24 | 0.27 | 0.29 | 0.30 | 0.31 | 0.34 | 0.36 | 0.38 | 0.41 | 0.44 | 0.46 | 0.48 | 0.51 | 0.53 | 0.55 | 0.58 | 0.59 | 0.61 | 0.64 | 0.66 | 0.68 | 0.70 | 0.72 |
| 200 | 0.16 | 0.17 | 0.17 | 0.17 | 0.17 | 0.18 | 0.20 | 0.22 | 0.25 | 0.26 | 0.27 | 0.30 | 0.32 | 0.35 | 0.37 | 0.40 | 0.42 | 0.46 | 0.49 | 0.52 | 0.56 | 0.58 | 0.62 | 0.65 | 0.68 | 0.70 | 0.73 | 0.75 | 0.77 | 0.80 |
| 220 | 0.16 | 0.16 | 0.16 | 0.16 | 0.17 | 0.19 | 0.21 | 0.22 | 0.25 | 0.27 | 0.29 | 0.31 | 0.35 | 0.37 | 0.40 | 0.43 | 0.47 | 0.51 | 0.55 | 0.58 | 0.61 | 0.66 | 0.69 | 0.72 | 0.75 | 0.76 | 0.80 | 0.82 | 0.85 | 0.86 |
| 240 | 0.14 | 0.15 | 0.15 | 0.16 | 0.17 | 0.18 | 0.20 | 0.23 | 0.27 | 0.31 | 0.35 | 0.38 | 0.41 | 0.44 | 0.48 | 0.51 | 0.55 | 0.59 | 0.62 | 0.66 | 0.69 | 0.72 | 0.76 | 0.79 | 0.82 | 0.85 | 0.87 | 0.89 | 0.91 | 0.92 |
| 260 | 0.14 | 0.14 | 0.16 | 0.17 | 0.20 | 0.22 | 0.25 | 0.29 | 0.32 | 0.36 | 0.40 | 0.45 | 0.48 | 0.52 | 0.56 | 0.61 | 0.65 | 0.69 | 0.72 | 0.77 | 0.80 | 0.82 | 0.84 | 0.86 | 0.88 | 0.91 | 0.93 | 0.94 | 0.95 | 0.96 |
| 280 | 0.14 | 0.14 | 0.16 | 0.18 | 0.20 | 0.22 | 0.26 | 0.29 | 0.34 | 0.39 | 0.45 | 0.49 | 0.53 | 0.58 | 0.63 | 0.67 | 0.71 | 0.75 | 0.79 | 0.82 | 0.85 | 0.87 | 0.89 | 0.90 | 0.93 | 0.95 | 0.96 | 0.97 | 0.98 | 0.98 |
| 300 | 0.13 | 0.14 | 0.15 | 0.16 | 0.20 | 0.23 | 0.26 | 0.30 | 0.35 | 0.41 | 0.45 | 0.51 | 0.56 | 0.61 | 0.67 | 0.72 | 0.76 | 0.80 | 0.84 | 0.87 | 0.90 | 0.93 | 0.94 | 0.96 | 0.96 | 0.97 | 0.97 | 0.98 | 0.99 | 0.99 |
| 320 | 0.11 | 0.12 | 0.14 | 0.17 | 0.21 | 0.26 | 0.31 | 0.35 | 0.40 | 0.44 | 0.51 | 0.57 | 0.63 | 0.68 | 0.72 | 0.77 | 0.80 | 0.85 | 0.88 | 0.91 | 0.93 | 0.95 | 0.97 | 0.97 | 0.98 | 0.99 | 0.99 | 1.00 | 1.00 | 1.00 |
| 340 | 0.12 | 0.12 | 0.15 | 0.19 | 0.22 | 0.26 | 0.32 | 0.37 | 0.43 | 0.49 | 0.56 | 0.62 | 0.69 | 0.73 | 0.79 | 0.83 | 0.87 | 0.90 | 0.92 | 0.94 | 0.95 | 0.97 | 0.98 | 0.98 | 0.99 | 0.99 | 0.99 | 1.00 | 1.00 | 1.00 |
| 360 | 0.11 | 0.13 | 0.16 | 0.19 | 0.22 | 0.27 | 0.32 | 0.39 | 0.45 | 0.52 | 0.59 | 0.64 | 0.71 | 0.77 | 0.82 | 0.87 | 0.90 | 0.93 | 0.95 | 0.96 | 0.98 | 0.98 | 0.99 | 0.99 | 1.00 | 1.00 | 1.00 | 1.00 | 1.00 | 1.00 |
| 380 | 0.12 | 0.15 | 0.18 | 0.22 | 0.29 | 0.35 | 0.41 | 0.48 | 0.56 | 0.63 | 0.68 | 0.74 | 0.80 | 0.84 | 0.88 | 0.93 | 0.95 | 0.97 | 0.98 | 0.99 | 0.99 | 0.99 | 1.00 | 1.00 | 1.00 | 1.00 | 1.00 | 1.00 | 1.00 | 1.00 |
| 400 | 0.12 | 0.13 | 0.17 | 0.23 | 0.29 | 0.36 | 0.42 | 0.50 | 0.58 | 0.64 | 0.72 | 0.78 | 0.82 | 0.87 | 0.90 | 0.93 | 0.95 | 0.97 | 0.99 | 0.99 | 1.00 | 1.00 | 1.00 | 1.00 | 1.00 | 1.00 | 1.00 | 1.00 | 1.00 | 1.00 |

Note: In the simulations it was assumed that there was no underlying trend or covariates which needed to be adjusted for. The intervention was assumed to have occurred at the midpoint of the time series.

Table 12: Power to detect a change in trend as a function of time and size of the change in trend, with the presence of AR= 0.1 autocorrelation and standard deviation of the white noise of 4.

|  | Effect size for the change in trend | | | | | | | | | | | | | | | | | | | | | | | | | | | | | |
| --- | --- | --- | --- | --- | --- | --- | --- | --- | --- | --- | --- | --- | --- | --- | --- | --- | --- | --- | --- | --- | --- | --- | --- | --- | --- | --- | --- | --- | --- | --- |
| Time | 0.01 | 0.02 | 0.03 | 0.04 | 0.05 | 0.06 | 0.07 | 0.08 | 0.09 | 0.1 | 0.11 | 0.12 | 0.13 | 0.14 | 0.15 | 0.16 | 0.17 | 0.18 | 0.19 | 0.2 | 0.21 | 0.22 | 0.23 | 0.24 | 0.25 | 0.26 | 0.27 | 0.28 | 0.29 | 0.3 |
| 40 | 0.10 | 0.10 | 0.09 | 0.09 | 0.09 | 0.09 | 0.10 | 0.11 | 0.11 | 0.12 | 0.13 | 0.13 | 0.14 | 0.15 | 0.16 | 0.16 | 0.17 | 0.18 | 0.19 | 0.20 | 0.21 | 0.23 | 0.24 | 0.25 | 0.25 | 0.26 | 0.27 | 0.28 | 0.29 | 0.31 |
| 60 | 0.08 | 0.08 | 0.09 | 0.09 | 0.10 | 0.11 | 0.12 | 0.13 | 0.15 | 0.16 | 0.18 | 0.20 | 0.21 | 0.23 | 0.25 | 0.28 | 0.30 | 0.32 | 0.34 | 0.38 | 0.42 | 0.44 | 0.47 | 0.51 | 0.54 | 0.57 | 0.60 | 0.63 | 0.66 | 0.69 |
| 80 | 0.09 | 0.09 | 0.10 | 0.11 | 0.13 | 0.15 | 0.17 | 0.20 | 0.24 | 0.27 | 0.30 | 0.34 | 0.38 | 0.42 | 0.47 | 0.53 | 0.57 | 0.61 | 0.65 | 0.69 | 0.73 | 0.77 | 0.79 | 0.83 | 0.86 | 0.89 | 0.90 | 0.93 | 0.93 | 0.95 |
| 100 | 0.07 | 0.07 | 0.09 | 0.11 | 0.15 | 0.19 | 0.25 | 0.31 | 0.37 | 0.42 | 0.48 | 0.55 | 0.62 | 0.67 | 0.72 | 0.76 | 0.81 | 0.85 | 0.89 | 0.92 | 0.94 | 0.96 | 0.97 | 0.98 | 0.99 | 0.99 | 1.00 | 1.00 | 1.00 | 1.00 |
| 120 | 0.07 | 0.09 | 0.11 | 0.14 | 0.20 | 0.28 | 0.36 | 0.43 | 0.51 | 0.58 | 0.67 | 0.74 | 0.82 | 0.86 | 0.90 | 0.94 | 0.96 | 0.98 | 0.98 | 0.99 | 1.00 | 1.00 | 1.00 | 1.00 | 1.00 | 1.00 | 1.00 | 1.00 | 1.00 | 1.00 |
| 140 | 0.08 | 0.12 | 0.16 | 0.24 | 0.31 | 0.41 | 0.53 | 0.63 | 0.72 | 0.80 | 0.87 | 0.91 | 0.95 | 0.97 | 0.98 | 0.99 | 1.00 | 1.00 | 1.00 | 1.00 | 1.00 | 1.00 | 1.00 | 1.00 | 1.00 | 1.00 | 1.00 | 1.00 | 1.00 | 1.00 |
| 160 | 0.08 | 0.12 | 0.19 | 0.29 | 0.42 | 0.55 | 0.66 | 0.76 | 0.86 | 0.93 | 0.96 | 0.98 | 0.99 | 1.00 | 1.00 | 1.00 | 1.00 | 1.00 | 1.00 | 1.00 | 1.00 | 1.00 | 1.00 | 1.00 | 1.00 | 1.00 | 1.00 | 1.00 | 1.00 | 1.00 |
| 180 | 0.07 | 0.13 | 0.24 | 0.34 | 0.52 | 0.67 | 0.80 | 0.89 | 0.95 | 0.99 | 0.99 | 1.00 | 1.00 | 1.00 | 1.00 | 1.00 | 1.00 | 1.00 | 1.00 | 1.00 | 1.00 | 1.00 | 1.00 | 1.00 | 1.00 | 1.00 | 1.00 | 1.00 | 1.00 | 1.00 |
| 200 | 0.09 | 0.15 | 0.31 | 0.48 | 0.67 | 0.79 | 0.90 | 0.95 | 0.98 | 1.00 | 1.00 | 1.00 | 1.00 | 1.00 | 1.00 | 1.00 | 1.00 | 1.00 | 1.00 | 1.00 | 1.00 | 1.00 | 1.00 | 1.00 | 1.00 | 1.00 | 1.00 | 1.00 | 1.00 | 1.00 |
| 220 | 0.10 | 0.20 | 0.39 | 0.59 | 0.78 | 0.90 | 0.96 | 0.99 | 1.00 | 1.00 | 1.00 | 1.00 | 1.00 | 1.00 | 1.00 | 1.00 | 1.00 | 1.00 | 1.00 | 1.00 | 1.00 | 1.00 | 1.00 | 1.00 | 1.00 | 1.00 | 1.00 | 1.00 | 1.00 | 1.00 |
| 240 | 0.11 | 0.23 | 0.46 | 0.67 | 0.86 | 0.95 | 0.99 | 1.00 | 1.00 | 1.00 | 1.00 | 1.00 | 1.00 | 1.00 | 1.00 | 1.00 | 1.00 | 1.00 | 1.00 | 1.00 | 1.00 | 1.00 | 1.00 | 1.00 | 1.00 | 1.00 | 1.00 | 1.00 | 1.00 | 1.00 |
| 260 | 0.11 | 0.30 | 0.57 | 0.81 | 0.94 | 0.98 | 1.00 | 1.00 | 1.00 | 1.00 | 1.00 | 1.00 | 1.00 | 1.00 | 1.00 | 1.00 | 1.00 | 1.00 | 1.00 | 1.00 | 1.00 | 1.00 | 1.00 | 1.00 | 1.00 | 1.00 | 1.00 | 1.00 | 1.00 | 1.00 |
| 280 | 0.12 | 0.32 | 0.62 | 0.86 | 0.96 | 1.00 | 1.00 | 1.00 | 1.00 | 1.00 | 1.00 | 1.00 | 1.00 | 1.00 | 1.00 | 1.00 | 1.00 | 1.00 | 1.00 | 1.00 | 1.00 | 1.00 | 1.00 | 1.00 | 1.00 | 1.00 | 1.00 | 1.00 | 1.00 | 1.00 |
| 300 | 0.17 | 0.43 | 0.73 | 0.92 | 0.99 | 1.00 | 1.00 | 1.00 | 1.00 | 1.00 | 1.00 | 1.00 | 1.00 | 1.00 | 1.00 | 1.00 | 1.00 | 1.00 | 1.00 | 1.00 | 1.00 | 1.00 | 1.00 | 1.00 | 1.00 | 1.00 | 1.00 | 1.00 | 1.00 | 1.00 |
| 320 | 0.18 | 0.52 | 0.83 | 0.98 | 1.00 | 1.00 | 1.00 | 1.00 | 1.00 | 1.00 | 1.00 | 1.00 | 1.00 | 1.00 | 1.00 | 1.00 | 1.00 | 1.00 | 1.00 | 1.00 | 1.00 | 1.00 | 1.00 | 1.00 | 1.00 | 1.00 | 1.00 | 1.00 | 1.00 | 1.00 |
| 340 | 0.17 | 0.51 | 0.88 | 0.98 | 1.00 | 1.00 | 1.00 | 1.00 | 1.00 | 1.00 | 1.00 | 1.00 | 1.00 | 1.00 | 1.00 | 1.00 | 1.00 | 1.00 | 1.00 | 1.00 | 1.00 | 1.00 | 1.00 | 1.00 | 1.00 | 1.00 | 1.00 | 1.00 | 1.00 | 1.00 |
| 360 | 0.21 | 0.63 | 0.92 | 1.00 | 1.00 | 1.00 | 1.00 | 1.00 | 1.00 | 1.00 | 1.00 | 1.00 | 1.00 | 1.00 | 1.00 | 1.00 | 1.00 | 1.00 | 1.00 | 1.00 | 1.00 | 1.00 | 1.00 | 1.00 | 1.00 | 1.00 | 1.00 | 1.00 | 1.00 | 1.00 |
| 380 | 0.26 | 0.68 | 0.94 | 1.00 | 1.00 | 1.00 | 1.00 | 1.00 | 1.00 | 1.00 | 1.00 | 1.00 | 1.00 | 1.00 | 1.00 | 1.00 | 1.00 | 1.00 | 1.00 | 1.00 | 1.00 | 1.00 | 1.00 | 1.00 | 1.00 | 1.00 | 1.00 | 1.00 | 1.00 | 1.00 |
| 400 | 0.26 | 0.73 | 0.98 | 1.00 | 1.00 | 1.00 | 1.00 | 1.00 | 1.00 | 1.00 | 1.00 | 1.00 | 1.00 | 1.00 | 1.00 | 1.00 | 1.00 | 1.00 | 1.00 | 1.00 | 1.00 | 1.00 | 1.00 | 1.00 | 1.00 | 1.00 | 1.00 | 1.00 | 1.00 | 1.00 |

Note: In the simulations it was assumed that there was no underlying trend or covariates which needed to be adjusted for. The intervention was assumed to have occurred at the midpoint of the time series.

Table 13: Power to detect a change in trend as a function of time and size of the change in trend, with the presence of AR= 0.5 autocorrelation and standard deviation of the white noise of 4.

|  | Effect size for the change in trend | | | | | | | | | | | | | | | | | | | | | | | | | | | | | |
| --- | --- | --- | --- | --- | --- | --- | --- | --- | --- | --- | --- | --- | --- | --- | --- | --- | --- | --- | --- | --- | --- | --- | --- | --- | --- | --- | --- | --- | --- | --- |
| Time | 0.01 | 0.02 | 0.03 | 0.04 | 0.05 | 0.06 | 0.07 | 0.08 | 0.09 | 0.1 | 0.11 | 0.12 | 0.13 | 0.14 | 0.15 | 0.16 | 0.17 | 0.18 | 0.19 | 0.2 | 0.21 | 0.22 | 0.23 | 0.24 | 0.25 | 0.26 | 0.27 | 0.28 | 0.29 | 0.3 |
| 40 | 0.13 | 0.14 | 0.14 | 0.14 | 0.14 | 0.14 | 0.15 | 0.15 | 0.15 | 0.15 | 0.16 | 0.16 | 0.16 | 0.16 | 0.17 | 0.17 | 0.18 | 0.18 | 0.19 | 0.20 | 0.20 | 0.20 | 0.21 | 0.22 | 0.22 | 0.23 | 0.23 | 0.24 | 0.25 | 0.25 |
| 60 | 0.13 | 0.13 | 0.13 | 0.13 | 0.13 | 0.13 | 0.13 | 0.13 | 0.13 | 0.13 | 0.14 | 0.14 | 0.14 | 0.16 | 0.16 | 0.18 | 0.19 | 0.20 | 0.21 | 0.22 | 0.23 | 0.24 | 0.26 | 0.27 | 0.28 | 0.30 | 0.31 | 0.32 | 0.33 | 0.35 |
| 80 | 0.08 | 0.08 | 0.09 | 0.10 | 0.11 | 0.12 | 0.13 | 0.13 | 0.14 | 0.16 | 0.18 | 0.20 | 0.21 | 0.22 | 0.24 | 0.26 | 0.28 | 0.31 | 0.33 | 0.35 | 0.38 | 0.39 | 0.41 | 0.45 | 0.47 | 0.51 | 0.53 | 0.56 | 0.58 | 0.61 |
| 100 | 0.08 | 0.09 | 0.10 | 0.10 | 0.11 | 0.13 | 0.14 | 0.17 | 0.20 | 0.22 | 0.26 | 0.28 | 0.30 | 0.33 | 0.37 | 0.41 | 0.43 | 0.47 | 0.51 | 0.55 | 0.58 | 0.61 | 0.65 | 0.68 | 0.71 | 0.73 | 0.76 | 0.79 | 0.82 | 0.85 |
| 120 | 0.08 | 0.09 | 0.11 | 0.12 | 0.13 | 0.16 | 0.18 | 0.21 | 0.24 | 0.28 | 0.32 | 0.37 | 0.40 | 0.45 | 0.50 | 0.54 | 0.59 | 0.64 | 0.69 | 0.73 | 0.77 | 0.80 | 0.83 | 0.85 | 0.88 | 0.90 | 0.92 | 0.94 | 0.95 | 0.97 |
| 140 | 0.06 | 0.07 | 0.09 | 0.11 | 0.14 | 0.17 | 0.21 | 0.26 | 0.33 | 0.38 | 0.44 | 0.51 | 0.57 | 0.62 | 0.67 | 0.72 | 0.76 | 0.81 | 0.85 | 0.88 | 0.91 | 0.93 | 0.94 | 0.96 | 0.97 | 0.98 | 0.99 | 1.00 | 1.00 | 1.00 |
| 160 | 0.08 | 0.10 | 0.13 | 0.16 | 0.21 | 0.26 | 0.31 | 0.38 | 0.43 | 0.51 | 0.58 | 0.66 | 0.73 | 0.78 | 0.84 | 0.87 | 0.91 | 0.94 | 0.96 | 0.98 | 0.98 | 0.99 | 0.99 | 1.00 | 1.00 | 1.00 | 1.00 | 1.00 | 1.00 | 1.00 |
| 180 | 0.07 | 0.10 | 0.13 | 0.20 | 0.26 | 0.33 | 0.39 | 0.49 | 0.57 | 0.65 | 0.73 | 0.80 | 0.87 | 0.90 | 0.94 | 0.96 | 0.98 | 0.99 | 0.99 | 1.00 | 1.00 | 1.00 | 1.00 | 1.00 | 1.00 | 1.00 | 1.00 | 1.00 | 1.00 | 1.00 |
| 200 | 0.08 | 0.11 | 0.15 | 0.21 | 0.27 | 0.36 | 0.45 | 0.58 | 0.67 | 0.77 | 0.85 | 0.90 | 0.94 | 0.96 | 0.99 | 0.99 | 1.00 | 1.00 | 1.00 | 1.00 | 1.00 | 1.00 | 1.00 | 1.00 | 1.00 | 1.00 | 1.00 | 1.00 | 1.00 | 1.00 |
| 220 | 0.09 | 0.12 | 0.18 | 0.25 | 0.37 | 0.47 | 0.58 | 0.69 | 0.79 | 0.85 | 0.92 | 0.95 | 0.97 | 0.98 | 0.99 | 1.00 | 1.00 | 1.00 | 1.00 | 1.00 | 1.00 | 1.00 | 1.00 | 1.00 | 1.00 | 1.00 | 1.00 | 1.00 | 1.00 | 1.00 |
| 240 | 0.08 | 0.11 | 0.20 | 0.29 | 0.41 | 0.54 | 0.68 | 0.78 | 0.87 | 0.93 | 0.97 | 0.99 | 1.00 | 1.00 | 1.00 | 1.00 | 1.00 | 1.00 | 1.00 | 1.00 | 1.00 | 1.00 | 1.00 | 1.00 | 1.00 | 1.00 | 1.00 | 1.00 | 1.00 | 1.00 |
| 260 | 0.09 | 0.14 | 0.25 | 0.38 | 0.52 | 0.67 | 0.79 | 0.88 | 0.95 | 0.98 | 0.99 | 1.00 | 1.00 | 1.00 | 1.00 | 1.00 | 1.00 | 1.00 | 1.00 | 1.00 | 1.00 | 1.00 | 1.00 | 1.00 | 1.00 | 1.00 | 1.00 | 1.00 | 1.00 | 1.00 |
| 280 | 0.10 | 0.16 | 0.27 | 0.44 | 0.59 | 0.73 | 0.85 | 0.93 | 0.97 | 0.99 | 1.00 | 1.00 | 1.00 | 1.00 | 1.00 | 1.00 | 1.00 | 1.00 | 1.00 | 1.00 | 1.00 | 1.00 | 1.00 | 1.00 | 1.00 | 1.00 | 1.00 | 1.00 | 1.00 | 1.00 |
| 300 | 0.09 | 0.16 | 0.29 | 0.48 | 0.64 | 0.79 | 0.90 | 0.96 | 0.99 | 1.00 | 1.00 | 1.00 | 1.00 | 1.00 | 1.00 | 1.00 | 1.00 | 1.00 | 1.00 | 1.00 | 1.00 | 1.00 | 1.00 | 1.00 | 1.00 | 1.00 | 1.00 | 1.00 | 1.00 | 1.00 |
| 320 | 0.09 | 0.18 | 0.35 | 0.55 | 0.75 | 0.88 | 0.96 | 0.99 | 1.00 | 1.00 | 1.00 | 1.00 | 1.00 | 1.00 | 1.00 | 1.00 | 1.00 | 1.00 | 1.00 | 1.00 | 1.00 | 1.00 | 1.00 | 1.00 | 1.00 | 1.00 | 1.00 | 1.00 | 1.00 | 1.00 |
| 340 | 0.09 | 0.23 | 0.42 | 0.64 | 0.81 | 0.93 | 0.98 | 1.00 | 1.00 | 1.00 | 1.00 | 1.00 | 1.00 | 1.00 | 1.00 | 1.00 | 1.00 | 1.00 | 1.00 | 1.00 | 1.00 | 1.00 | 1.00 | 1.00 | 1.00 | 1.00 | 1.00 | 1.00 | 1.00 | 1.00 |
| 360 | 0.11 | 0.26 | 0.48 | 0.73 | 0.88 | 0.96 | 0.99 | 1.00 | 1.00 | 1.00 | 1.00 | 1.00 | 1.00 | 1.00 | 1.00 | 1.00 | 1.00 | 1.00 | 1.00 | 1.00 | 1.00 | 1.00 | 1.00 | 1.00 | 1.00 | 1.00 | 1.00 | 1.00 | 1.00 | 1.00 |
| 380 | 0.11 | 0.30 | 0.56 | 0.79 | 0.93 | 0.98 | 1.00 | 1.00 | 1.00 | 1.00 | 1.00 | 1.00 | 1.00 | 1.00 | 1.00 | 1.00 | 1.00 | 1.00 | 1.00 | 1.00 | 1.00 | 1.00 | 1.00 | 1.00 | 1.00 | 1.00 | 1.00 | 1.00 | 1.00 | 1.00 |
| 400 | 0.12 | 0.33 | 0.61 | 0.83 | 0.94 | 0.99 | 1.00 | 1.00 | 1.00 | 1.00 | 1.00 | 1.00 | 1.00 | 1.00 | 1.00 | 1.00 | 1.00 | 1.00 | 1.00 | 1.00 | 1.00 | 1.00 | 1.00 | 1.00 | 1.00 | 1.00 | 1.00 | 1.00 | 1.00 | 1.00 |

Note: In the simulations it was assumed that there was no underlying trend or covariates which needed to be adjusted for. The intervention was assumed to have occurred at the midpoint of the time series.

Table 14: Power to detect a change in trend as a function of time and size of the change in trend, with the presence of AR= 0.9 autocorrelation and standard deviation of the white noise of 4.

|  | Effect size for the change in trend | | | | | | | | | | | | | | | | | | | | | | | | | | | | | |
| --- | --- | --- | --- | --- | --- | --- | --- | --- | --- | --- | --- | --- | --- | --- | --- | --- | --- | --- | --- | --- | --- | --- | --- | --- | --- | --- | --- | --- | --- | --- |
| Time | 0.01 | 0.02 | 0.03 | 0.04 | 0.05 | 0.06 | 0.07 | 0.08 | 0.09 | 0.1 | 0.11 | 0.12 | 0.13 | 0.14 | 0.15 | 0.16 | 0.17 | 0.18 | 0.19 | 0.2 | 0.21 | 0.22 | 0.23 | 0.24 | 0.25 | 0.26 | 0.27 | 0.28 | 0.29 | 0.3 |
| 40 | 0.38 | 0.39 | 0.39 | 0.39 | 0.39 | 0.39 | 0.39 | 0.39 | 0.39 | 0.39 | 0.39 | 0.39 | 0.39 | 0.39 | 0.39 | 0.40 | 0.40 | 0.40 | 0.40 | 0.40 | 0.40 | 0.40 | 0.40 | 0.40 | 0.41 | 0.41 | 0.40 | 0.41 | 0.41 | 0.41 |
| 60 | 0.29 | 0.29 | 0.29 | 0.29 | 0.29 | 0.29 | 0.29 | 0.29 | 0.29 | 0.29 | 0.29 | 0.29 | 0.29 | 0.29 | 0.29 | 0.29 | 0.29 | 0.30 | 0.29 | 0.30 | 0.30 | 0.30 | 0.30 | 0.30 | 0.31 | 0.31 | 0.31 | 0.31 | 0.31 | 0.31 |
| 80 | 0.26 | 0.26 | 0.26 | 0.27 | 0.27 | 0.27 | 0.27 | 0.27 | 0.28 | 0.28 | 0.28 | 0.28 | 0.28 | 0.29 | 0.29 | 0.29 | 0.29 | 0.30 | 0.29 | 0.30 | 0.31 | 0.32 | 0.32 | 0.33 | 0.33 | 0.33 | 0.34 | 0.34 | 0.35 | 0.36 |
| 100 | 0.21 | 0.22 | 0.21 | 0.22 | 0.22 | 0.22 | 0.23 | 0.23 | 0.24 | 0.24 | 0.24 | 0.24 | 0.24 | 0.24 | 0.25 | 0.25 | 0.26 | 0.27 | 0.27 | 0.28 | 0.29 | 0.29 | 0.30 | 0.31 | 0.31 | 0.32 | 0.32 | 0.33 | 0.33 | 0.34 |
| 120 | 0.19 | 0.19 | 0.19 | 0.18 | 0.19 | 0.19 | 0.19 | 0.20 | 0.21 | 0.21 | 0.22 | 0.23 | 0.24 | 0.25 | 0.26 | 0.26 | 0.27 | 0.27 | 0.28 | 0.28 | 0.29 | 0.29 | 0.30 | 0.31 | 0.32 | 0.32 | 0.33 | 0.34 | 0.36 | 0.36 |
| 140 | 0.17 | 0.17 | 0.18 | 0.18 | 0.18 | 0.19 | 0.19 | 0.19 | 0.20 | 0.20 | 0.20 | 0.21 | 0.22 | 0.22 | 0.23 | 0.24 | 0.25 | 0.26 | 0.27 | 0.28 | 0.30 | 0.30 | 0.31 | 0.33 | 0.35 | 0.36 | 0.37 | 0.39 | 0.40 | 0.41 |
| 160 | 0.17 | 0.17 | 0.18 | 0.18 | 0.18 | 0.18 | 0.20 | 0.20 | 0.20 | 0.21 | 0.22 | 0.24 | 0.25 | 0.26 | 0.26 | 0.27 | 0.29 | 0.31 | 0.32 | 0.32 | 0.34 | 0.35 | 0.36 | 0.37 | 0.39 | 0.40 | 0.41 | 0.43 | 0.45 | 0.47 |
| 180 | 0.18 | 0.18 | 0.19 | 0.19 | 0.19 | 0.20 | 0.20 | 0.21 | 0.22 | 0.23 | 0.25 | 0.27 | 0.28 | 0.29 | 0.30 | 0.32 | 0.34 | 0.35 | 0.37 | 0.38 | 0.40 | 0.42 | 0.44 | 0.46 | 0.47 | 0.50 | 0.51 | 0.53 | 0.54 | 0.56 |
| 200 | 0.15 | 0.16 | 0.17 | 0.17 | 0.17 | 0.18 | 0.17 | 0.18 | 0.20 | 0.22 | 0.23 | 0.25 | 0.26 | 0.27 | 0.28 | 0.30 | 0.32 | 0.34 | 0.35 | 0.37 | 0.39 | 0.41 | 0.43 | 0.46 | 0.48 | 0.51 | 0.53 | 0.56 | 0.58 | 0.60 |
| 220 | 0.16 | 0.16 | 0.16 | 0.16 | 0.16 | 0.17 | 0.18 | 0.19 | 0.20 | 0.22 | 0.23 | 0.25 | 0.26 | 0.28 | 0.30 | 0.31 | 0.34 | 0.36 | 0.38 | 0.40 | 0.43 | 0.45 | 0.48 | 0.51 | 0.54 | 0.56 | 0.58 | 0.61 | 0.64 | 0.67 |
| 240 | 0.14 | 0.14 | 0.15 | 0.15 | 0.16 | 0.16 | 0.17 | 0.19 | 0.20 | 0.22 | 0.24 | 0.27 | 0.29 | 0.33 | 0.36 | 0.38 | 0.40 | 0.43 | 0.45 | 0.48 | 0.50 | 0.53 | 0.56 | 0.59 | 0.61 | 0.64 | 0.66 | 0.69 | 0.71 | 0.74 |
| 260 | 0.13 | 0.14 | 0.14 | 0.16 | 0.17 | 0.18 | 0.20 | 0.22 | 0.24 | 0.27 | 0.30 | 0.32 | 0.35 | 0.38 | 0.41 | 0.45 | 0.47 | 0.50 | 0.53 | 0.56 | 0.60 | 0.63 | 0.66 | 0.69 | 0.71 | 0.75 | 0.78 | 0.80 | 0.82 | 0.83 |
| 280 | 0.13 | 0.14 | 0.14 | 0.15 | 0.17 | 0.19 | 0.21 | 0.22 | 0.25 | 0.28 | 0.30 | 0.34 | 0.38 | 0.42 | 0.46 | 0.49 | 0.53 | 0.55 | 0.59 | 0.64 | 0.66 | 0.69 | 0.73 | 0.75 | 0.78 | 0.80 | 0.83 | 0.85 | 0.87 | 0.88 |
| 300 | 0.13 | 0.14 | 0.14 | 0.14 | 0.16 | 0.18 | 0.20 | 0.23 | 0.25 | 0.29 | 0.31 | 0.35 | 0.40 | 0.43 | 0.46 | 0.51 | 0.55 | 0.58 | 0.63 | 0.68 | 0.72 | 0.74 | 0.78 | 0.80 | 0.83 | 0.86 | 0.88 | 0.90 | 0.92 | 0.93 |
| 320 | 0.11 | 0.11 | 0.12 | 0.15 | 0.16 | 0.19 | 0.22 | 0.26 | 0.29 | 0.33 | 0.37 | 0.40 | 0.43 | 0.48 | 0.52 | 0.57 | 0.62 | 0.66 | 0.69 | 0.71 | 0.75 | 0.79 | 0.82 | 0.85 | 0.87 | 0.90 | 0.92 | 0.93 | 0.95 | 0.96 |
| 340 | 0.11 | 0.11 | 0.13 | 0.15 | 0.18 | 0.21 | 0.23 | 0.26 | 0.30 | 0.35 | 0.39 | 0.43 | 0.48 | 0.52 | 0.57 | 0.62 | 0.67 | 0.71 | 0.74 | 0.79 | 0.82 | 0.85 | 0.88 | 0.90 | 0.92 | 0.93 | 0.95 | 0.95 | 0.96 | 0.97 |
| 360 | 0.11 | 0.12 | 0.13 | 0.15 | 0.18 | 0.21 | 0.23 | 0.27 | 0.31 | 0.36 | 0.41 | 0.45 | 0.50 | 0.56 | 0.61 | 0.64 | 0.69 | 0.73 | 0.78 | 0.82 | 0.85 | 0.89 | 0.91 | 0.93 | 0.94 | 0.95 | 0.97 | 0.98 | 0.98 | 0.99 |
| 380 | 0.12 | 0.13 | 0.15 | 0.18 | 0.21 | 0.26 | 0.30 | 0.35 | 0.39 | 0.45 | 0.50 | 0.56 | 0.61 | 0.66 | 0.70 | 0.74 | 0.78 | 0.82 | 0.85 | 0.88 | 0.91 | 0.94 | 0.96 | 0.97 | 0.98 | 0.98 | 0.99 | 0.99 | 0.99 | 1.00 |
| 400 | 0.11 | 0.12 | 0.14 | 0.17 | 0.22 | 0.25 | 0.31 | 0.36 | 0.42 | 0.46 | 0.53 | 0.58 | 0.63 | 0.68 | 0.73 | 0.77 | 0.81 | 0.85 | 0.88 | 0.90 | 0.92 | 0.95 | 0.96 | 0.97 | 0.99 | 0.99 | 0.99 | 1.00 | 1.00 | 1.00 |

Note: In the simulations it was assumed that there was no underlying trend or covariates which needed to be adjusted for. The intervention was assumed to have occurred at the midpoint of the time series.

Table 15: Power to detect a change in trend as a function of time and size of the change in trend, with the presence of AR= 0.1 autocorrelation and standard deviation of the white noise of 5.

|  | Effect size for the change in trend | | | | | | | | | | | | | | | | | | | | | | | | | | | | | |
| --- | --- | --- | --- | --- | --- | --- | --- | --- | --- | --- | --- | --- | --- | --- | --- | --- | --- | --- | --- | --- | --- | --- | --- | --- | --- | --- | --- | --- | --- | --- |
| Time | 0.01 | 0.02 | 0.03 | 0.04 | 0.05 | 0.06 | 0.07 | 0.08 | 0.09 | 0.1 | 0.11 | 0.12 | 0.13 | 0.14 | 0.15 | 0.16 | 0.17 | 0.18 | 0.19 | 0.2 | 0.21 | 0.22 | 0.23 | 0.24 | 0.25 | 0.26 | 0.27 | 0.28 | 0.29 | 0.3 |
| 40 |  |  |  |  |  |  |  |  |  |  |  |  |  |  |  |  |  |  |  |  |  |  |  |  |  |  |  |  |  |  |
| 60 | 0.10 | 0.10 | 0.10 | 0.09 | 0.09 | 0.09 | 0.09 | 0.10 | 0.10 | 0.11 | 0.11 | 0.12 | 0.12 | 0.13 | 0.13 | 0.13 | 0.14 | 0.15 | 0.16 | 0.16 | 0.17 | 0.18 | 0.19 | 0.19 | 0.20 | 0.21 | 0.22 | 0.23 | 0.24 | 0.25 |
| 80 | 0.08 | 0.08 | 0.09 | 0.09 | 0.09 | 0.10 | 0.11 | 0.11 | 0.12 | 0.13 | 0.15 | 0.16 | 0.17 | 0.18 | 0.20 | 0.21 | 0.23 | 0.24 | 0.26 | 0.28 | 0.29 | 0.31 | 0.33 | 0.35 | 0.38 | 0.41 | 0.43 | 0.45 | 0.48 | 0.51 |
| 100 | 0.09 | 0.09 | 0.09 | 0.10 | 0.11 | 0.13 | 0.14 | 0.16 | 0.18 | 0.20 | 0.23 | 0.26 | 0.29 | 0.31 | 0.34 | 0.37 | 0.40 | 0.44 | 0.48 | 0.53 | 0.56 | 0.60 | 0.63 | 0.66 | 0.69 | 0.71 | 0.75 | 0.77 | 0.80 | 0.83 |
| 120 | 0.07 | 0.07 | 0.08 | 0.09 | 0.11 | 0.14 | 0.17 | 0.21 | 0.26 | 0.31 | 0.36 | 0.40 | 0.45 | 0.49 | 0.55 | 0.60 | 0.65 | 0.69 | 0.73 | 0.76 | 0.80 | 0.84 | 0.87 | 0.90 | 0.92 | 0.94 | 0.96 | 0.97 | 0.98 | 0.98 |
| 140 | 0.07 | 0.08 | 0.09 | 0.11 | 0.14 | 0.19 | 0.24 | 0.30 | 0.37 | 0.43 | 0.49 | 0.55 | 0.62 | 0.69 | 0.74 | 0.80 | 0.85 | 0.88 | 0.91 | 0.94 | 0.96 | 0.97 | 0.98 | 0.98 | 0.99 | 1.00 | 1.00 | 1.00 | 1.00 | 1.00 |
| 160 | 0.07 | 0.09 | 0.13 | 0.18 | 0.24 | 0.29 | 0.38 | 0.46 | 0.55 | 0.63 | 0.71 | 0.77 | 0.84 | 0.88 | 0.91 | 0.94 | 0.97 | 0.98 | 0.99 | 0.99 | 1.00 | 1.00 | 1.00 | 1.00 | 1.00 | 1.00 | 1.00 | 1.00 | 1.00 | 1.00 |
| 180 | 0.07 | 0.10 | 0.15 | 0.21 | 0.29 | 0.39 | 0.50 | 0.59 | 0.68 | 0.76 | 0.84 | 0.90 | 0.94 | 0.96 | 0.98 | 0.99 | 1.00 | 1.00 | 1.00 | 1.00 | 1.00 | 1.00 | 1.00 | 1.00 | 1.00 | 1.00 | 1.00 | 1.00 | 1.00 | 1.00 |
| 200 | 0.07 | 0.10 | 0.17 | 0.26 | 0.34 | 0.48 | 0.61 | 0.73 | 0.83 | 0.89 | 0.94 | 0.97 | 0.99 | 0.99 | 1.00 | 1.00 | 1.00 | 1.00 | 1.00 | 1.00 | 1.00 | 1.00 | 1.00 | 1.00 | 1.00 | 1.00 | 1.00 | 1.00 | 1.00 | 1.00 |
| 220 | 0.08 | 0.11 | 0.21 | 0.35 | 0.48 | 0.64 | 0.73 | 0.83 | 0.91 | 0.95 | 0.98 | 0.99 | 1.00 | 1.00 | 1.00 | 1.00 | 1.00 | 1.00 | 1.00 | 1.00 | 1.00 | 1.00 | 1.00 | 1.00 | 1.00 | 1.00 | 1.00 | 1.00 | 1.00 | 1.00 |
| 240 | 0.08 | 0.16 | 0.26 | 0.43 | 0.59 | 0.75 | 0.86 | 0.93 | 0.97 | 0.99 | 1.00 | 1.00 | 1.00 | 1.00 | 1.00 | 1.00 | 1.00 | 1.00 | 1.00 | 1.00 | 1.00 | 1.00 | 1.00 | 1.00 | 1.00 | 1.00 | 1.00 | 1.00 | 1.00 | 1.00 |
| 260 | 0.09 | 0.17 | 0.33 | 0.52 | 0.67 | 0.81 | 0.92 | 0.97 | 0.99 | 1.00 | 1.00 | 1.00 | 1.00 | 1.00 | 1.00 | 1.00 | 1.00 | 1.00 | 1.00 | 1.00 | 1.00 | 1.00 | 1.00 | 1.00 | 1.00 | 1.00 | 1.00 | 1.00 | 1.00 | 1.00 |
| 280 | 0.08 | 0.21 | 0.42 | 0.62 | 0.81 | 0.91 | 0.97 | 0.99 | 1.00 | 1.00 | 1.00 | 1.00 | 1.00 | 1.00 | 1.00 | 1.00 | 1.00 | 1.00 | 1.00 | 1.00 | 1.00 | 1.00 | 1.00 | 1.00 | 1.00 | 1.00 | 1.00 | 1.00 | 1.00 | 1.00 |
| 300 | 0.10 | 0.23 | 0.45 | 0.67 | 0.86 | 0.95 | 0.99 | 1.00 | 1.00 | 1.00 | 1.00 | 1.00 | 1.00 | 1.00 | 1.00 | 1.00 | 1.00 | 1.00 | 1.00 | 1.00 | 1.00 | 1.00 | 1.00 | 1.00 | 1.00 | 1.00 | 1.00 | 1.00 | 1.00 | 1.00 |
| 320 | 0.14 | 0.30 | 0.56 | 0.78 | 0.92 | 0.98 | 1.00 | 1.00 | 1.00 | 1.00 | 1.00 | 1.00 | 1.00 | 1.00 | 1.00 | 1.00 | 1.00 | 1.00 | 1.00 | 1.00 | 1.00 | 1.00 | 1.00 | 1.00 | 1.00 | 1.00 | 1.00 | 1.00 | 1.00 | 1.00 |
| 340 | 0.14 | 0.36 | 0.65 | 0.88 | 0.98 | 1.00 | 1.00 | 1.00 | 1.00 | 1.00 | 1.00 | 1.00 | 1.00 | 1.00 | 1.00 | 1.00 | 1.00 | 1.00 | 1.00 | 1.00 | 1.00 | 1.00 | 1.00 | 1.00 | 1.00 | 1.00 | 1.00 | 1.00 | 1.00 | 1.00 |
| 360 | 0.12 | 0.36 | 0.66 | 0.91 | 0.98 | 1.00 | 1.00 | 1.00 | 1.00 | 1.00 | 1.00 | 1.00 | 1.00 | 1.00 | 1.00 | 1.00 | 1.00 | 1.00 | 1.00 | 1.00 | 1.00 | 1.00 | 1.00 | 1.00 | 1.00 | 1.00 | 1.00 | 1.00 | 1.00 | 1.00 |
| 380 | 0.15 | 0.46 | 0.78 | 0.95 | 1.00 | 1.00 | 1.00 | 1.00 | 1.00 | 1.00 | 1.00 | 1.00 | 1.00 | 1.00 | 1.00 | 1.00 | 1.00 | 1.00 | 1.00 | 1.00 | 1.00 | 1.00 | 1.00 | 1.00 | 1.00 | 1.00 | 1.00 | 1.00 | 1.00 | 1.00 |
| 400 | 0.18 | 0.51 | 0.84 | 0.97 | 1.00 | 1.00 | 1.00 | 1.00 | 1.00 | 1.00 | 1.00 | 1.00 | 1.00 | 1.00 | 1.00 | 1.00 | 1.00 | 1.00 | 1.00 | 1.00 | 1.00 | 1.00 | 1.00 | 1.00 | 1.00 | 1.00 | 1.00 | 1.00 | 1.00 | 1.00 |

Note: In the simulations it was assumed that there was no underlying trend or covariates which needed to be adjusted for. The intervention was assumed to have occurred at the midpoint of the time series.

Table 16: Power to detect a change in trend as a function of time and size of the change in trend, with the presence of AR= 0.5 autocorrelation and standard deviation of the white noise of 5.

|  | Effect size for the change in trend | | | | | | | | | | | | | | | | | | | | | | | | | | | | | |
| --- | --- | --- | --- | --- | --- | --- | --- | --- | --- | --- | --- | --- | --- | --- | --- | --- | --- | --- | --- | --- | --- | --- | --- | --- | --- | --- | --- | --- | --- | --- |
| Time | 0.01 | 0.02 | 0.03 | 0.04 | 0.05 | 0.06 | 0.07 | 0.08 | 0.09 | 0.1 | 0.11 | 0.12 | 0.13 | 0.14 | 0.15 | 0.16 | 0.17 | 0.18 | 0.19 | 0.2 | 0.21 | 0.22 | 0.23 | 0.24 | 0.25 | 0.26 | 0.27 | 0.28 | 0.29 | 0.3 |
| 40 | 0.13 | 0.13 | 0.14 | 0.14 | 0.14 | 0.14 | 0.14 | 0.14 | 0.15 | 0.15 | 0.15 | 0.15 | 0.15 | 0.15 | 0.16 | 0.16 | 0.16 | 0.16 | 0.17 | 0.17 | 0.18 | 0.18 | 0.18 | 0.19 | 0.20 | 0.20 | 0.20 | 0.21 | 0.21 | 0.22 |
| 60 | 0.13 | 0.13 | 0.13 | 0.13 | 0.13 | 0.13 | 0.13 | 0.13 | 0.13 | 0.13 | 0.13 | 0.13 | 0.13 | 0.14 | 0.14 | 0.14 | 0.15 | 0.16 | 0.17 | 0.18 | 0.19 | 0.19 | 0.21 | 0.22 | 0.22 | 0.23 | 0.24 | 0.25 | 0.26 | 0.27 |
| 80 | 0.08 | 0.08 | 0.09 | 0.09 | 0.10 | 0.10 | 0.11 | 0.12 | 0.13 | 0.13 | 0.14 | 0.16 | 0.17 | 0.18 | 0.20 | 0.21 | 0.22 | 0.23 | 0.24 | 0.26 | 0.28 | 0.29 | 0.32 | 0.33 | 0.35 | 0.37 | 0.39 | 0.40 | 0.42 | 0.45 |
| 100 | 0.08 | 0.09 | 0.10 | 0.10 | 0.10 | 0.11 | 0.12 | 0.13 | 0.15 | 0.17 | 0.19 | 0.21 | 0.23 | 0.26 | 0.28 | 0.30 | 0.32 | 0.35 | 0.38 | 0.41 | 0.42 | 0.45 | 0.49 | 0.52 | 0.55 | 0.57 | 0.60 | 0.63 | 0.66 | 0.68 |
| 120 | 0.09 | 0.08 | 0.10 | 0.11 | 0.12 | 0.13 | 0.14 | 0.17 | 0.19 | 0.21 | 0.23 | 0.26 | 0.30 | 0.33 | 0.37 | 0.39 | 0.43 | 0.47 | 0.51 | 0.54 | 0.57 | 0.62 | 0.66 | 0.70 | 0.73 | 0.76 | 0.78 | 0.81 | 0.83 | 0.85 |
| 140 | 0.06 | 0.06 | 0.08 | 0.10 | 0.11 | 0.13 | 0.16 | 0.19 | 0.22 | 0.26 | 0.31 | 0.36 | 0.40 | 0.45 | 0.51 | 0.56 | 0.60 | 0.64 | 0.69 | 0.72 | 0.75 | 0.80 | 0.83 | 0.86 | 0.88 | 0.90 | 0.93 | 0.94 | 0.95 | 0.96 |
| 160 | 0.08 | 0.09 | 0.12 | 0.14 | 0.16 | 0.20 | 0.24 | 0.28 | 0.33 | 0.38 | 0.42 | 0.49 | 0.53 | 0.59 | 0.66 | 0.72 | 0.76 | 0.81 | 0.84 | 0.87 | 0.90 | 0.93 | 0.95 | 0.97 | 0.98 | 0.98 | 0.99 | 0.99 | 0.99 | 1.00 |
| 180 | 0.06 | 0.08 | 0.11 | 0.14 | 0.20 | 0.24 | 0.30 | 0.36 | 0.42 | 0.49 | 0.56 | 0.62 | 0.68 | 0.75 | 0.80 | 0.85 | 0.89 | 0.92 | 0.95 | 0.96 | 0.98 | 0.99 | 0.99 | 0.99 | 1.00 | 1.00 | 1.00 | 1.00 | 1.00 | 1.00 |
| 200 | 0.07 | 0.09 | 0.12 | 0.16 | 0.21 | 0.26 | 0.33 | 0.40 | 0.48 | 0.58 | 0.65 | 0.72 | 0.80 | 0.86 | 0.90 | 0.93 | 0.95 | 0.97 | 0.99 | 0.99 | 1.00 | 1.00 | 1.00 | 1.00 | 1.00 | 1.00 | 1.00 | 1.00 | 1.00 | 1.00 |
| 220 | 0.09 | 0.11 | 0.14 | 0.19 | 0.25 | 0.34 | 0.43 | 0.51 | 0.60 | 0.69 | 0.77 | 0.83 | 0.88 | 0.92 | 0.95 | 0.97 | 0.98 | 0.99 | 1.00 | 1.00 | 1.00 | 1.00 | 1.00 | 1.00 | 1.00 | 1.00 | 1.00 | 1.00 | 1.00 | 1.00 |
| 240 | 0.08 | 0.10 | 0.14 | 0.21 | 0.29 | 0.39 | 0.49 | 0.60 | 0.70 | 0.78 | 0.86 | 0.91 | 0.95 | 0.97 | 0.99 | 0.99 | 1.00 | 1.00 | 1.00 | 1.00 | 1.00 | 1.00 | 1.00 | 1.00 | 1.00 | 1.00 | 1.00 | 1.00 | 1.00 | 1.00 |
| 260 | 0.07 | 0.12 | 0.19 | 0.28 | 0.38 | 0.49 | 0.62 | 0.72 | 0.81 | 0.88 | 0.94 | 0.97 | 0.98 | 0.99 | 1.00 | 1.00 | 1.00 | 1.00 | 1.00 | 1.00 | 1.00 | 1.00 | 1.00 | 1.00 | 1.00 | 1.00 | 1.00 | 1.00 | 1.00 | 1.00 |
| 280 | 0.09 | 0.13 | 0.20 | 0.30 | 0.44 | 0.56 | 0.69 | 0.79 | 0.87 | 0.93 | 0.96 | 0.99 | 1.00 | 1.00 | 1.00 | 1.00 | 1.00 | 1.00 | 1.00 | 1.00 | 1.00 | 1.00 | 1.00 | 1.00 | 1.00 | 1.00 | 1.00 | 1.00 | 1.00 | 1.00 |
| 300 | 0.08 | 0.12 | 0.20 | 0.32 | 0.48 | 0.61 | 0.75 | 0.84 | 0.92 | 0.96 | 0.98 | 1.00 | 1.00 | 1.00 | 1.00 | 1.00 | 1.00 | 1.00 | 1.00 | 1.00 | 1.00 | 1.00 | 1.00 | 1.00 | 1.00 | 1.00 | 1.00 | 1.00 | 1.00 | 1.00 |
| 320 | 0.08 | 0.13 | 0.25 | 0.40 | 0.55 | 0.71 | 0.84 | 0.91 | 0.97 | 0.99 | 1.00 | 1.00 | 1.00 | 1.00 | 1.00 | 1.00 | 1.00 | 1.00 | 1.00 | 1.00 | 1.00 | 1.00 | 1.00 | 1.00 | 1.00 | 1.00 | 1.00 | 1.00 | 1.00 | 1.00 |
| 340 | 0.08 | 0.17 | 0.30 | 0.46 | 0.64 | 0.79 | 0.89 | 0.95 | 0.99 | 1.00 | 1.00 | 1.00 | 1.00 | 1.00 | 1.00 | 1.00 | 1.00 | 1.00 | 1.00 | 1.00 | 1.00 | 1.00 | 1.00 | 1.00 | 1.00 | 1.00 | 1.00 | 1.00 | 1.00 | 1.00 |
| 360 | 0.09 | 0.18 | 0.34 | 0.53 | 0.73 | 0.86 | 0.93 | 0.98 | 0.99 | 1.00 | 1.00 | 1.00 | 1.00 | 1.00 | 1.00 | 1.00 | 1.00 | 1.00 | 1.00 | 1.00 | 1.00 | 1.00 | 1.00 | 1.00 | 1.00 | 1.00 | 1.00 | 1.00 | 1.00 | 1.00 |
| 380 | 0.10 | 0.20 | 0.40 | 0.61 | 0.79 | 0.91 | 0.96 | 0.99 | 1.00 | 1.00 | 1.00 | 1.00 | 1.00 | 1.00 | 1.00 | 1.00 | 1.00 | 1.00 | 1.00 | 1.00 | 1.00 | 1.00 | 1.00 | 1.00 | 1.00 | 1.00 | 1.00 | 1.00 | 1.00 | 1.00 |
| 400 | 0.09 | 0.22 | 0.44 | 0.66 | 0.83 | 0.93 | 0.98 | 1.00 | 1.00 | 1.00 | 1.00 | 1.00 | 1.00 | 1.00 | 1.00 | 1.00 | 1.00 | 1.00 | 1.00 | 1.00 | 1.00 | 1.00 | 1.00 | 1.00 | 1.00 | 1.00 | 1.00 | 1.00 | 1.00 | 1.00 |

Note: In the simulations it was assumed that there was no underlying trend or covariates which needed to be adjusted for. The intervention was assumed to have occurred at the midpoint of the time series.

Table 17: Power to detect a change in trend as a function of time and size of the change in trend, with the presence of AR= 0.9 autocorrelation and standard deviation of the white noise of 5.

|  | Effect size for the change in trend | | | | | | | | | | | | | | | | | | | | | | | | | | | | | |
| --- | --- | --- | --- | --- | --- | --- | --- | --- | --- | --- | --- | --- | --- | --- | --- | --- | --- | --- | --- | --- | --- | --- | --- | --- | --- | --- | --- | --- | --- | --- |
| Time | 0.01 | 0.02 | 0.03 | 0.04 | 0.05 | 0.06 | 0.07 | 0.08 | 0.09 | 0.1 | 0.11 | 0.12 | 0.13 | 0.14 | 0.15 | 0.16 | 0.17 | 0.18 | 0.19 | 0.2 | 0.21 | 0.22 | 0.23 | 0.24 | 0.25 | 0.26 | 0.27 | 0.28 | 0.29 | 0.3 |
| 40 | 0.39 | 0.39 | 0.39 | 0.39 | 0.39 | 0.39 | 0.39 | 0.39 | 0.39 | 0.39 | 0.39 | 0.39 | 0.39 | 0.39 | 0.39 | 0.39 | 0.39 | 0.39 | 0.39 | 0.39 | 0.40 | 0.40 | 0.40 | 0.40 | 0.40 | 0.40 | 0.40 | 0.40 | 0.40 | 0.40 |
| 60 | 0.29 | 0.29 | 0.29 | 0.29 | 0.29 | 0.29 | 0.29 | 0.29 | 0.29 | 0.28 | 0.29 | 0.29 | 0.29 | 0.29 | 0.29 | 0.29 | 0.29 | 0.29 | 0.29 | 0.29 | 0.29 | 0.29 | 0.30 | 0.30 | 0.30 | 0.30 | 0.30 | 0.30 | 0.30 | 0.30 |
| 80 | 0.26 | 0.25 | 0.26 | 0.26 | 0.27 | 0.27 | 0.27 | 0.27 | 0.27 | 0.27 | 0.28 | 0.28 | 0.28 | 0.28 | 0.28 | 0.29 | 0.29 | 0.29 | 0.29 | 0.29 | 0.29 | 0.30 | 0.29 | 0.30 | 0.30 | 0.31 | 0.31 | 0.32 | 0.32 | 0.33 |
| 100 | 0.21 | 0.21 | 0.22 | 0.22 | 0.22 | 0.22 | 0.22 | 0.22 | 0.23 | 0.23 | 0.23 | 0.24 | 0.24 | 0.24 | 0.24 | 0.24 | 0.25 | 0.25 | 0.25 | 0.25 | 0.26 | 0.26 | 0.27 | 0.28 | 0.28 | 0.28 | 0.29 | 0.29 | 0.30 | 0.30 |
| 120 | 0.19 | 0.19 | 0.19 | 0.19 | 0.18 | 0.19 | 0.19 | 0.19 | 0.19 | 0.20 | 0.20 | 0.21 | 0.21 | 0.22 | 0.23 | 0.24 | 0.25 | 0.25 | 0.26 | 0.26 | 0.26 | 0.27 | 0.27 | 0.28 | 0.28 | 0.29 | 0.29 | 0.29 | 0.30 | 0.31 |
| 140 | 0.17 | 0.17 | 0.18 | 0.18 | 0.18 | 0.18 | 0.18 | 0.19 | 0.19 | 0.19 | 0.20 | 0.20 | 0.20 | 0.21 | 0.22 | 0.22 | 0.22 | 0.23 | 0.23 | 0.24 | 0.25 | 0.26 | 0.26 | 0.27 | 0.29 | 0.29 | 0.30 | 0.31 | 0.31 | 0.33 |
| 160 | 0.16 | 0.17 | 0.17 | 0.18 | 0.18 | 0.18 | 0.19 | 0.19 | 0.20 | 0.19 | 0.20 | 0.21 | 0.22 | 0.23 | 0.24 | 0.25 | 0.25 | 0.26 | 0.27 | 0.28 | 0.29 | 0.30 | 0.31 | 0.32 | 0.32 | 0.33 | 0.34 | 0.35 | 0.36 | 0.38 |
| 180 | 0.18 | 0.18 | 0.18 | 0.19 | 0.19 | 0.19 | 0.20 | 0.20 | 0.20 | 0.21 | 0.22 | 0.23 | 0.24 | 0.25 | 0.27 | 0.28 | 0.29 | 0.30 | 0.31 | 0.32 | 0.33 | 0.35 | 0.36 | 0.37 | 0.39 | 0.40 | 0.42 | 0.43 | 0.44 | 0.46 |
| 200 | 0.15 | 0.16 | 0.17 | 0.17 | 0.17 | 0.17 | 0.18 | 0.17 | 0.17 | 0.18 | 0.19 | 0.21 | 0.22 | 0.23 | 0.25 | 0.26 | 0.27 | 0.27 | 0.28 | 0.30 | 0.31 | 0.33 | 0.34 | 0.36 | 0.37 | 0.39 | 0.40 | 0.42 | 0.44 | 0.46 |
| 220 | 0.16 | 0.16 | 0.16 | 0.16 | 0.16 | 0.16 | 0.17 | 0.17 | 0.18 | 0.19 | 0.20 | 0.21 | 0.22 | 0.23 | 0.25 | 0.26 | 0.27 | 0.28 | 0.30 | 0.31 | 0.34 | 0.36 | 0.37 | 0.39 | 0.40 | 0.42 | 0.44 | 0.46 | 0.49 | 0.52 |
| 240 | 0.14 | 0.14 | 0.15 | 0.15 | 0.15 | 0.15 | 0.16 | 0.16 | 0.17 | 0.19 | 0.19 | 0.21 | 0.22 | 0.24 | 0.27 | 0.29 | 0.31 | 0.34 | 0.36 | 0.38 | 0.39 | 0.42 | 0.44 | 0.46 | 0.48 | 0.50 | 0.51 | 0.54 | 0.56 | 0.59 |
| 260 | 0.13 | 0.14 | 0.14 | 0.14 | 0.15 | 0.17 | 0.17 | 0.19 | 0.20 | 0.22 | 0.24 | 0.25 | 0.27 | 0.30 | 0.32 | 0.34 | 0.37 | 0.40 | 0.43 | 0.45 | 0.46 | 0.49 | 0.52 | 0.54 | 0.56 | 0.59 | 0.62 | 0.64 | 0.66 | 0.69 |
| 280 | 0.13 | 0.14 | 0.14 | 0.14 | 0.15 | 0.17 | 0.18 | 0.19 | 0.21 | 0.22 | 0.24 | 0.26 | 0.28 | 0.31 | 0.34 | 0.37 | 0.41 | 0.44 | 0.47 | 0.49 | 0.52 | 0.54 | 0.56 | 0.60 | 0.64 | 0.65 | 0.67 | 0.70 | 0.73 | 0.75 |
| 300 | 0.13 | 0.13 | 0.14 | 0.14 | 0.14 | 0.15 | 0.17 | 0.19 | 0.21 | 0.23 | 0.25 | 0.27 | 0.30 | 0.31 | 0.35 | 0.38 | 0.41 | 0.44 | 0.46 | 0.51 | 0.54 | 0.57 | 0.60 | 0.64 | 0.68 | 0.71 | 0.73 | 0.75 | 0.78 | 0.80 |
| 320 | 0.11 | 0.11 | 0.11 | 0.13 | 0.14 | 0.16 | 0.18 | 0.20 | 0.22 | 0.25 | 0.29 | 0.31 | 0.35 | 0.37 | 0.40 | 0.43 | 0.45 | 0.49 | 0.54 | 0.57 | 0.61 | 0.64 | 0.67 | 0.70 | 0.71 | 0.74 | 0.78 | 0.80 | 0.82 | 0.85 |
| 340 | 0.11 | 0.12 | 0.12 | 0.14 | 0.15 | 0.17 | 0.20 | 0.22 | 0.24 | 0.26 | 0.29 | 0.33 | 0.36 | 0.40 | 0.43 | 0.47 | 0.50 | 0.53 | 0.58 | 0.62 | 0.66 | 0.70 | 0.72 | 0.75 | 0.79 | 0.82 | 0.84 | 0.87 | 0.88 | 0.90 |
| 360 | 0.11 | 0.12 | 0.12 | 0.14 | 0.15 | 0.17 | 0.20 | 0.21 | 0.24 | 0.27 | 0.31 | 0.33 | 0.37 | 0.42 | 0.45 | 0.49 | 0.53 | 0.58 | 0.61 | 0.64 | 0.69 | 0.71 | 0.75 | 0.79 | 0.82 | 0.85 | 0.87 | 0.90 | 0.91 | 0.93 |
| 380 | 0.11 | 0.12 | 0.14 | 0.15 | 0.18 | 0.20 | 0.24 | 0.28 | 0.31 | 0.35 | 0.38 | 0.43 | 0.47 | 0.52 | 0.56 | 0.61 | 0.64 | 0.68 | 0.71 | 0.74 | 0.77 | 0.81 | 0.84 | 0.86 | 0.88 | 0.90 | 0.93 | 0.95 | 0.96 | 0.97 |
| 400 | 0.12 | 0.12 | 0.13 | 0.15 | 0.17 | 0.21 | 0.24 | 0.27 | 0.32 | 0.36 | 0.40 | 0.44 | 0.49 | 0.54 | 0.58 | 0.62 | 0.66 | 0.71 | 0.75 | 0.77 | 0.80 | 0.83 | 0.86 | 0.88 | 0.90 | 0.92 | 0.94 | 0.95 | 0.96 | 0.97 |

Note: In the simulations it was assumed that there was no underlying trend or covariates which needed to be adjusted for. The intervention was assumed to have occurred at the midpoint of the time series.
