# Supplementary material for: Sample size requirements to evaluate policies in addiction research using interrupted time series analysis (ITS): Tools and guidance
Source: Addiction. 2025 Nov 11;121(3):695–712. doi: 10.1111/add.70220 (PMC12887929; doi:10.1111/add.70220)
Supplement: Supplementary file 5 — Appendix S5. Examples of Shiny app configurations. [file ADD-121-695-s004.docx]

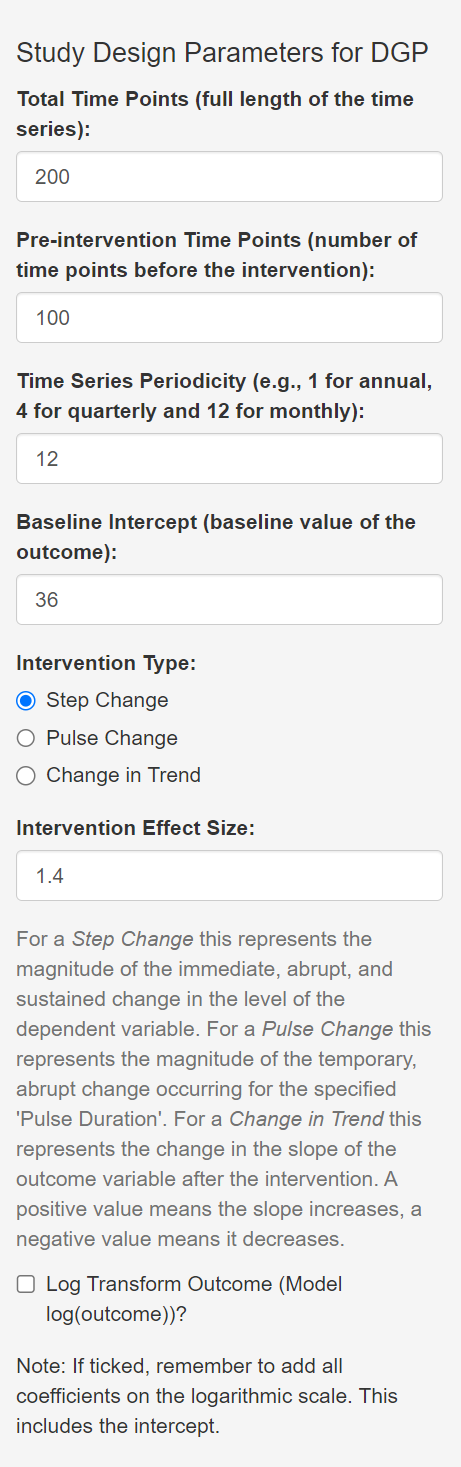
 **
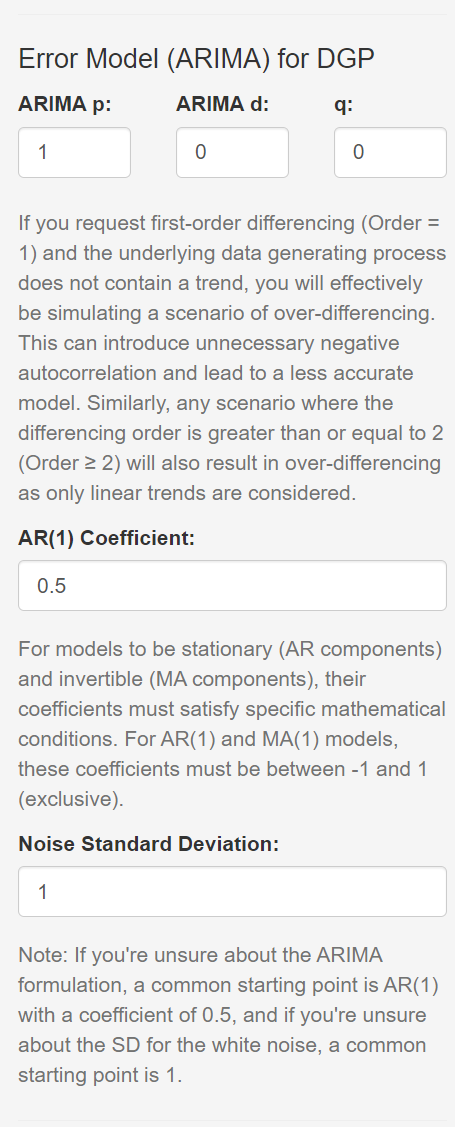
**
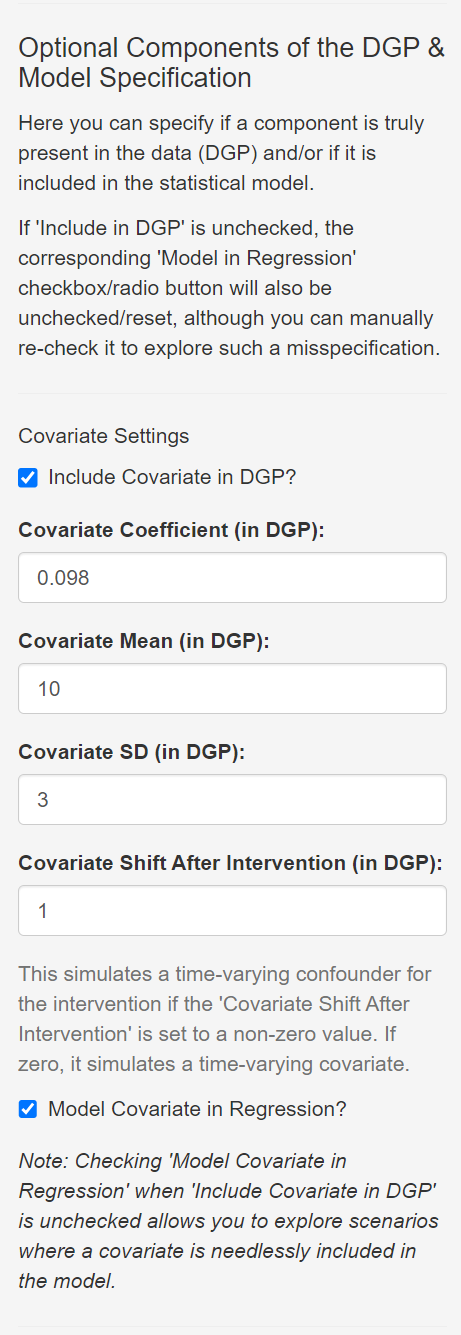

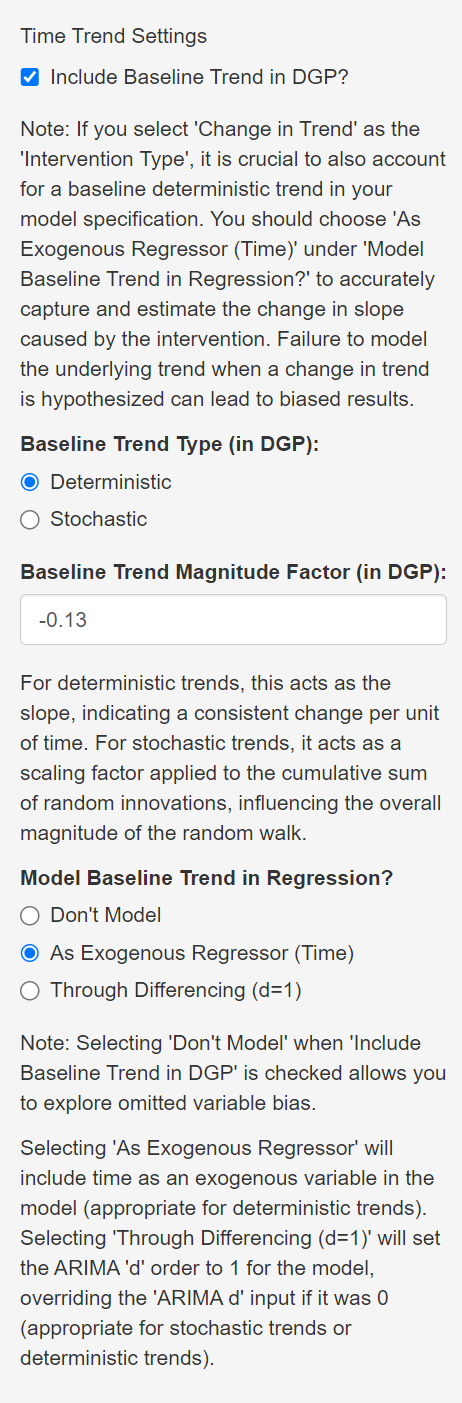

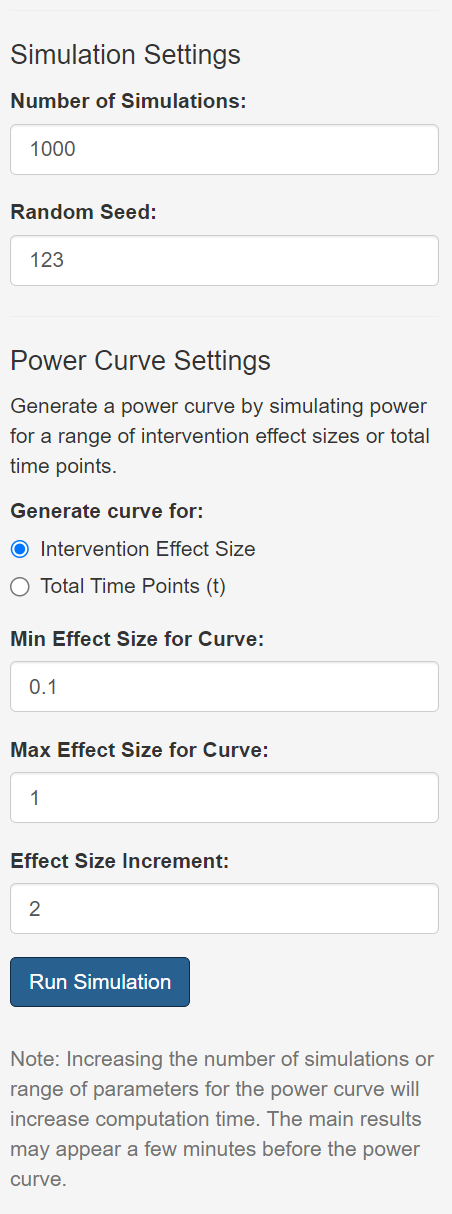


**Figure 1:** Configuration of the Shiny App for the illustrative example of the introduction of plain packaging of tobacco products in England


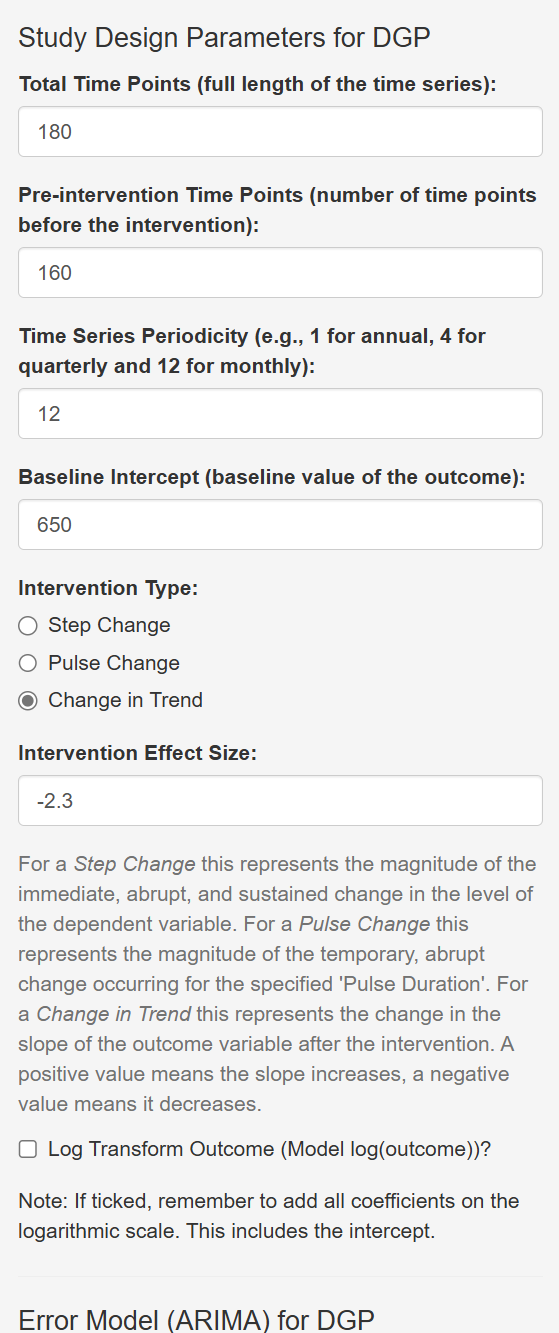
 **
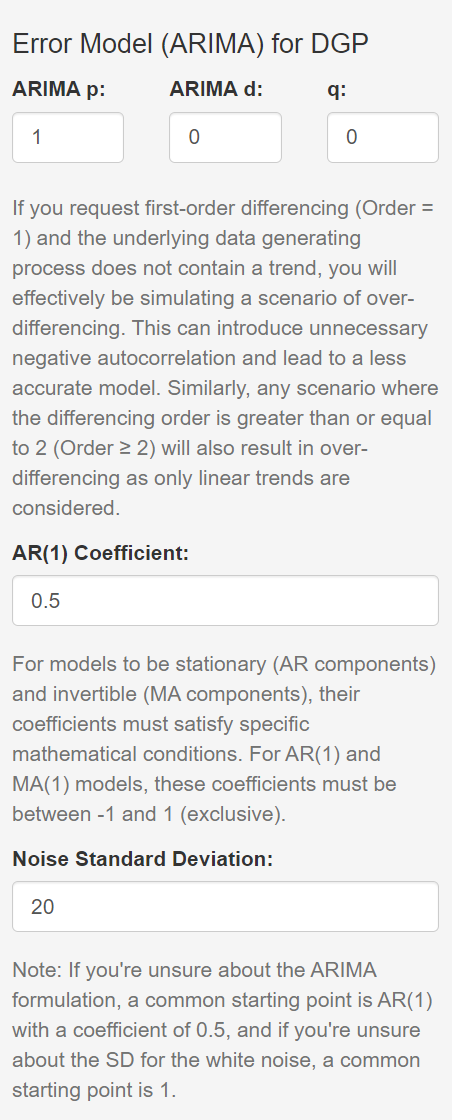
**
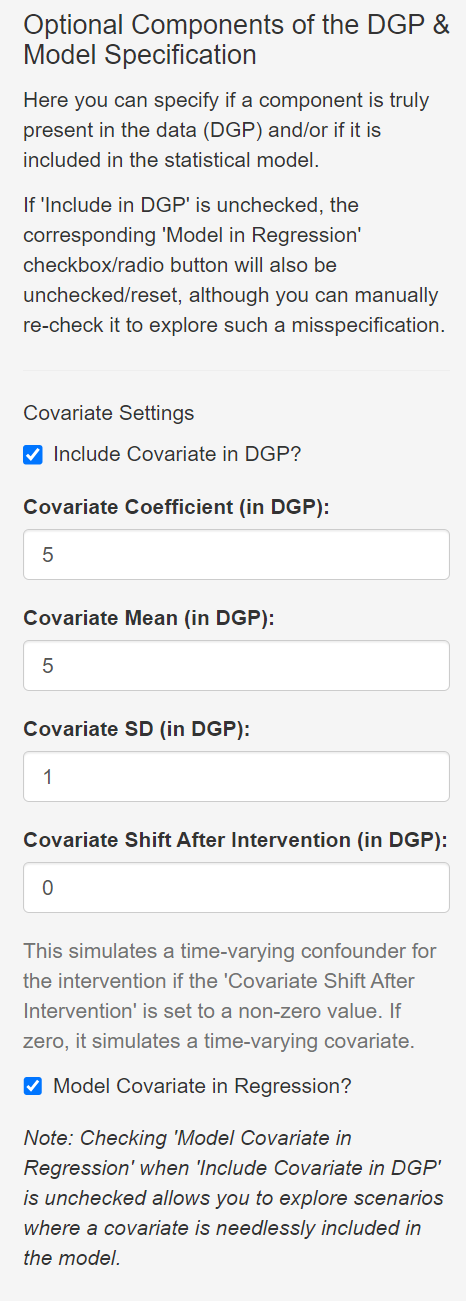

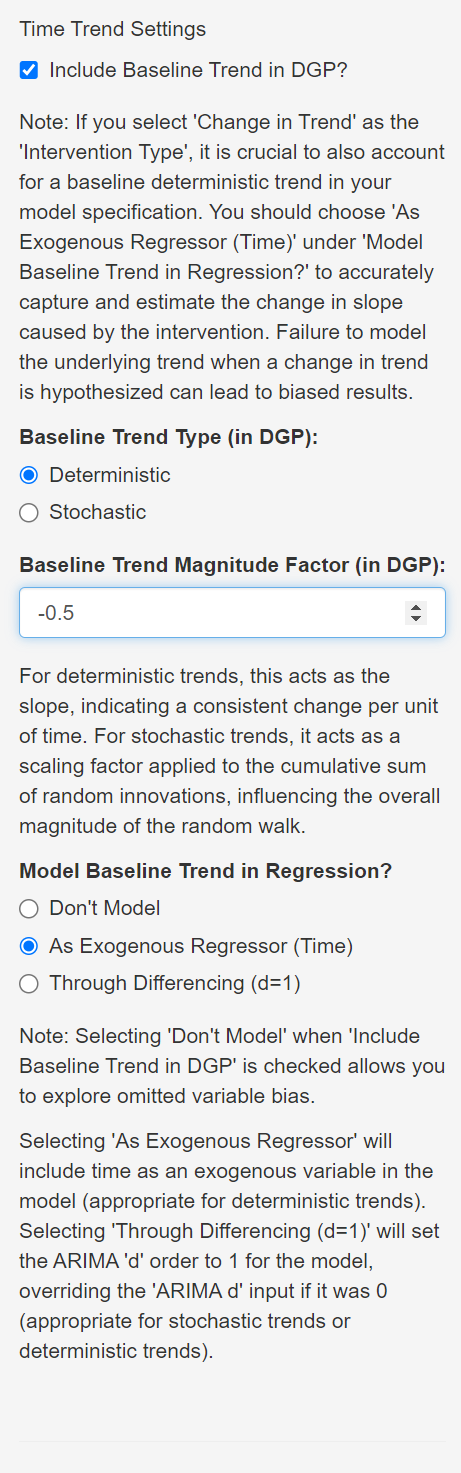

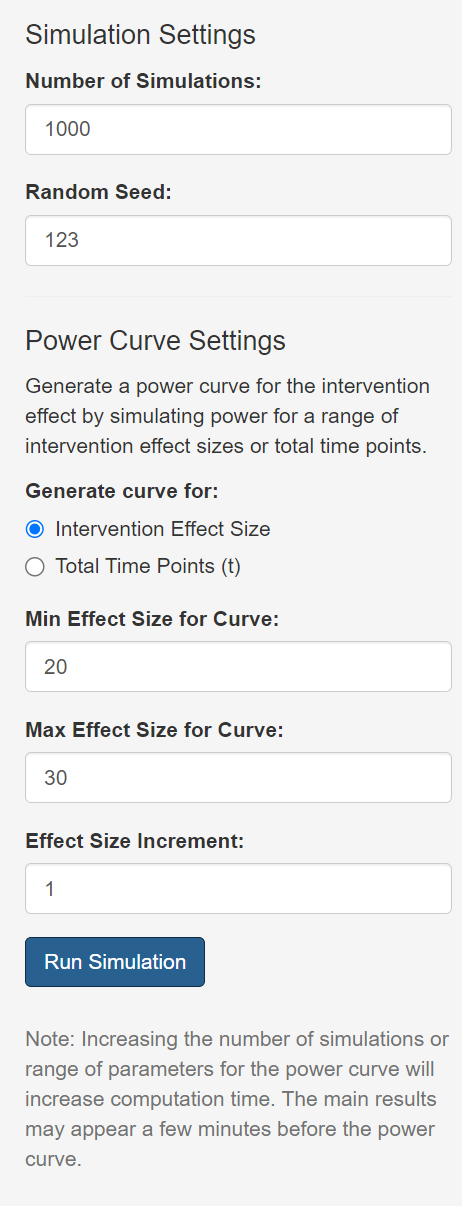


**Figure 2:** Configuration of the Shiny App for the illustrative example of MUP in Scotland

**
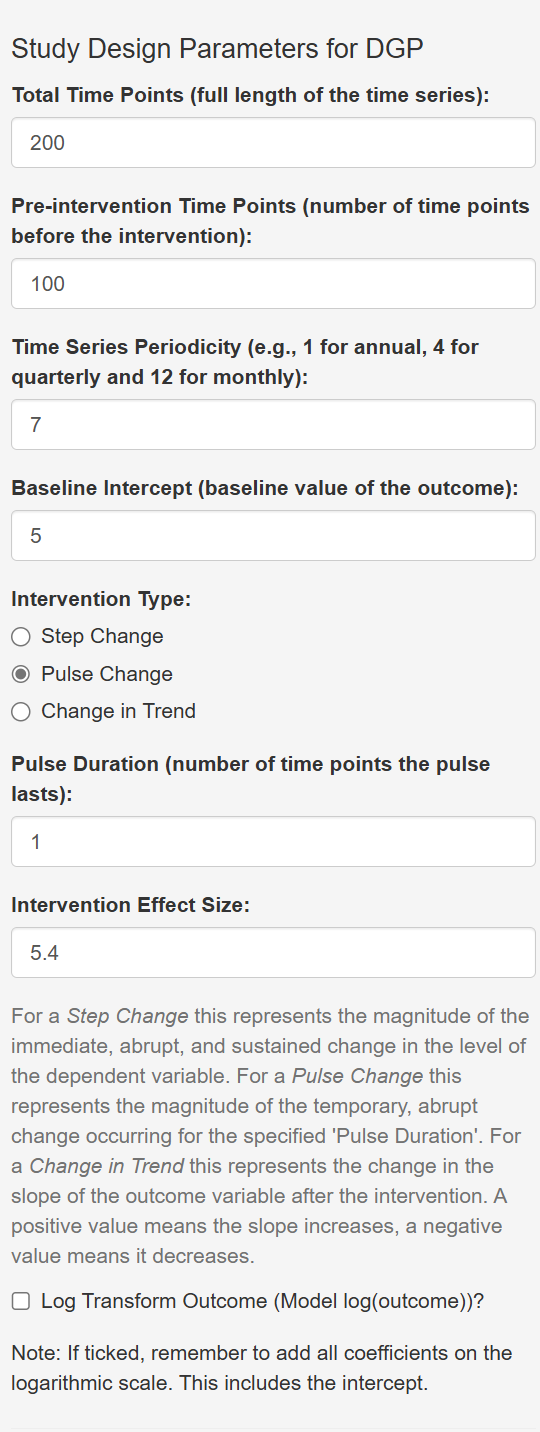
** **
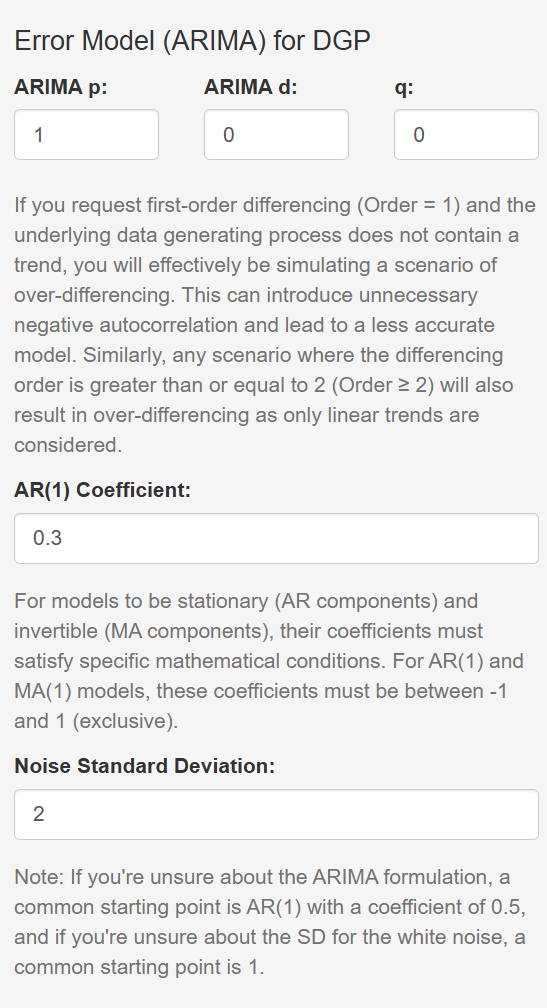
**
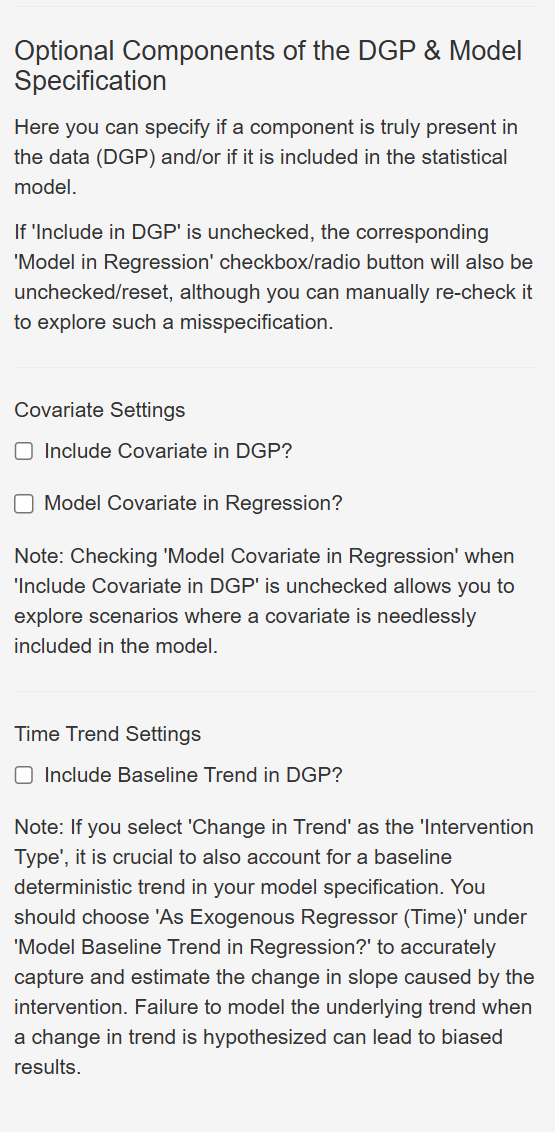

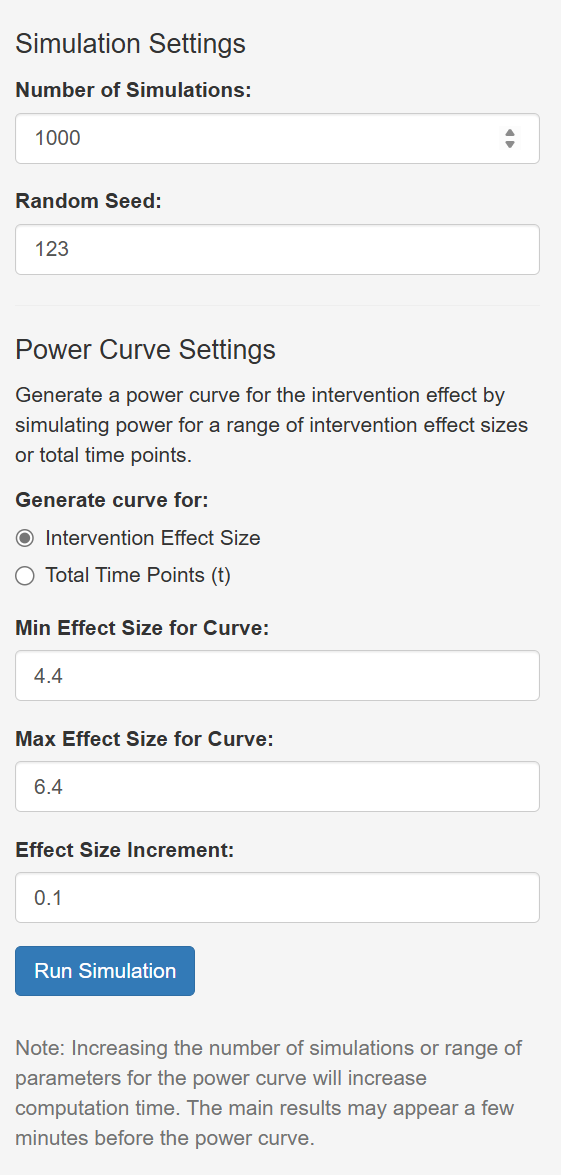


**Figure 3:** Configuration of the Shiny App for the illustrative example of the National prescription drug take-back day
